# Aloe Emodin Alleviates Radiation‐Induced Heart Disease via Blocking P4HB Lactylation and Mitigating Kynurenine Metabolic Disruption

**DOI:** 10.1002/advs.202406026

**Published:** 2024-11-04

**Authors:** Fan Ouyang, Yaling Li, Haoming Wang, Xiangyang Liu, Xiaoli Tan, Genyuan Xie, Junfa Zeng, Gaofeng Zeng, Qiong Luo, Hong Zhou, Siming Chen, Kai Hou, Jinren Fang, Xia Zhang, Linlin Zhou, Yukun Li, Anbo Gao

**Affiliations:** ^1^ Department of Cardiovascular Medicine Zhuzhou Hospital Affiliated to Xiangya School of Medicine Central South University Zhuzhou Hunan 412000 P. R. China; ^2^ Zhuzhou Clinical College Jishou University Jishou Hunan 416000 P. R. China; ^3^ Department of Critical Care Medicine Hengyang Medical School The Second Affiliated Hospital University of South China Hengyang Hunan 421001 P. R. China; ^4^ Clinical Research Institute The Second Affiliated Hospital Hengyang Medical School University of South China Hengyang Hunan 421001 P. R. China; ^5^ Department of Assisted Reproductive Centre Zhuzhou Hospital Affiliated to Xiangya School of Medicine Central South University Zhuzhou Hunan 412000 P. R. China; ^6^ Clinical Research Center for Arteriosclerotic Disease in Hunan Province Hengyang Hunan 421001 P. R. China; ^7^ Department of Radiology Hengyang Medical School The First Affiliated Hospital University of South China Hengyang Hunan 421001 P. R. China; ^8^ Department of Cardiovascular Medicine The Second Affiliated Hospital Hengyang Medical School University of South China Hengyang Hunan 421001 P. R. China; ^9^ Department of Ultrasound Medicine Hengyang Medical School The Second Affiliated Hospital University of South China Hengyang Hunan 421001 P. R. China; ^10^ Hunan Provincial Key Laboratory of Basic and Clinical Pharmacological Research of Gastrointestinal Cancer Hengyang Hunan 421001 P. R. China

**Keywords:** aloe emodin, kynurenine metabolism, lactylation, mitophagy, radiation‐induced heart damage

## Abstract

Aloe emodin is an anthraquinone of traditional Chinese medicine monomer, which plays a protective action in cardiovascular diseases. However, the regulatory mechanisms of aloe emodin in the protection of radiation‐induced heart damage (RIHD) are unclear. As a novel post‐translational modification, lactylation is considered as a critical mediator in inflammatory cascade and cardiac injury. Here, using a cross of differential omics and 4D label‐free lactylation omics, protein disulfide‐isomerase (P4HB) is identified as a novel target for lactylation, and aloe emodin inhibits the binding of lactate to the K311 site of P4HB. Aloe emodin stabilizes kynurenine metabolism through inhibition of aspartate aminotransferase (GOT2) accumulation on damaged mitochondria. Mechanistically, aloe emodin inhibits phosphorylated glycogen synthase kinase 3B (p‐GSK3B) transcription in the nucleus to repress the interaction of prostaglandin G/H synthase 2 (PTGS2) with SH3 domain of SH3 domain‐containing GRB2‐like protein B1 (SH3GLB1), thereby disrupting the functions of mitochondrial complexes and reducing SH3GLB1‐mediated mitoROS accumulation, eventually suppressing calcium‐binding and coiled‐coil domain‐containing protein 2 (NDP52)‐induced mitophagy. This study unveils the regulatory role of aloe emodin in RIHD alleviation through PTGS2/SH3GLB1/NDP52 axis, indicates aloe emodin stabilizes GOT2‐mediated kynurenine metabolism through P4HB lactylation. Collectively, this study provides novel insights into the regulatory mechanisms underlying the protective role of aloe emodin in cardiac injury, and opens new avenues for therapeutic strategies of aloe emodin in preventing RIHD by regulating lactylation.

## Graphical abstract

The study reveals that aloe emodin alleviated radiation‐induced heart disease (RIHD) through mitophagy inhibition. The underlying mechanism involves K311 site‐linked lactylation of protein disulfide‐isomerase (P4HB) and stabilization of aspartate aminotransferase (GOT2)‐mediated disrupted kynurenine metabolism, which provides a new therapeutic strategy that aloe emodin stabilizes GOT2‐mediated kynurenine metabolism to reduce inflammatory cascade through PTGS2/SH3GLB1/NDP52 axis via regulating K311‐linked P4HB lactylation, eventually ameliorate RIHD.

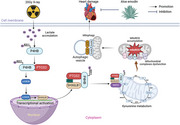

## 1 Introduction

With the demand of cancer patients for the improvement of their survival rate and quality of life after radiotherapy treatment, the protection of radiotherapy‐related cardiovascular diseases has led to increased awareness and recognition.^[^ ^1^ ^]^ Cardiovascular diseases induced by radiation include coronary artery, valvular disease, pericardial disease, cardiac fibrosis, and heart damage caused by autonomic dysfunction.^[^ ^2^, ^3^ ^]^ Radiation‐induced heart damage (RIHD) is an inevitable serious non‐carcinogenic side effect during radiotherapy treatment for thoracic tumors including Hodgkin's lymphoma, lung cancer, and breast cancer.^[^ ^4^ ^]^ Radiation exhibits an exceeding role in provoking dysfunctions and disrupted structure of the heart tissue, mainly involving pathological alterations in the myocardium, with cardiac fibrosis being the most observed manifestation.^[^ ^4^ ^]^ Although accumulating evidence suggests inflammatory cascade plays a critical role in the induction of cardiac fibrosis, the specific mechanism of radiation in regulating the inflammatory response in cardiomyocytes is unclear.

Although mitophagy is identified as a “double‐edged sword” in pathophysiological processes, the inflammatory response may be a mediator for the promotion of mitophagy activation in provoking RIHD.^[^ ^5^, ^6^ ^]^ Recent study has reported that Runt‐related transcription factor 1 (RUNX1) upregulated BCL2/adenovirus E1B 19 kDa protein‐interacting protein 3‐like (BNIP3L) expression and promoted autophagic influx for mitophagy activation, resulting in the alleviation of pulmonary inflammation in acute lung injury.^[^ ^7^ ^]^ Conversely, Danielle et al. have indicated that PINK1/Parkin‐mediated mitophagy inhibited the accumulation of mitochondrial DNA mutation, which reduced stimulator of interferon genes protein (STING)‐induced inflammation.^[^ ^8^ ^]^ However, Tsai‐Chun et al. have revealed that PM_2.5_ and high glucose were simultaneously harmful to endothelial cells by inducing ROS accumulation and mitophagy activation.^[^ ^9^ ^]^ Therefore, the precise protein target that determines the exact pathophysiological role of mitophagy is a key important step to explore the relationship between mitophagy and RIHD. Calcium‐binding and coiled‐coil domain‐containing protein 2 (NDP52) is identified as a crucial mediator for mitophagy activation through PINK1/Parkin signaling pathway.^[^ ^10^ ^]^ Our previous study has indicated that low‐dose X‐ray upregulated NDP52 expression to induce the fusion of damaged mitochondria and lysosomes for degradation via activity of PINK1 and Parkin.^[^ ^11^ ^]^ Therefore, seeking targeted new drugs for NDP52‐induced mitophagy activation may be an important opportunity to alleviate the progression of RIHD, and further exploration is needed to determine which targeted drugs are involved in the promotion of mitophagy in RIHD.

Aloe emodin, found in Aloe vera or *Rheum palmatum* L., is a natural anthraquinone derivative.^[^ ^12^ ^]^ Aloe emodin has been used as a raw material for multiple pharmaceuticals and was identified as an effective agent for anti‐inflammation and inhibition of ROS accumulation. It is a traditional Chinese medicine monomer for protecting against heart diseases.^[^ ^13^ ^]^ Yu et al. have demonstrated that aloe emodin protects against myocardial infarction (MI) via inhibition of ROS production.^[^ ^14^ ^]^ In the present study, we found that aloe emodin alleviated cardiac fibrosis through inhibition of NDP52‐induced mitophagy via repressing SH3 domain‐containing GRB2‐like protein B1 (SH3GLB1)‐mediated accumulation of mitoROS. However, the regulatory mechanism of aloe emodin in mitophagy via NDP52/SH3GLB1 needs further investigation. As a novel posttranslational modification (PTM), lactylation was demonstrated to be an epigenetic regulator for the modulation of substrate proteins at the lysine residues.^[^ ^15^ ^]^ Lactate accumulation was regarded as a simple energy source for cells' metabolic waste, and could regulate gene expression through lactylation, which has linked cell metabolism with the regulation of protein modification.^[^ ^16^ ^]^ Research has indicated a positive association between high levels of lactate and cardiac fibrosis, in which lactate promoted exacerbated cardiac dysfunction by promoting endothelial‐to‐mesenchymal transition (EndoMT) through activation of TGF‐β/Smad2 pathway. Mechanistically, lactate induced interaction of CBP/p300 and Snail1, leading to lactylation of Snail1, and further upregulated cardiac fibrosis after MI.^[^ ^17^ ^]^ Emerging evidence has elucidated the potential relationship between lactylation and radiation‐induced cardiac fibrosis, which is facilitated through the accumulation of mitoROS to induce mitophagy activation. However, it is not yet clear what metabolic pathways or mechanisms are regulated by lactylation to cause mitochondrial dysfunction and mitoROS accumulation. Moreover, further exploration is needed to determine whether aloe emodin can inhibit lactylation and alleviate cardiac fibrosis for the protection of RIHD.

In this study, we indicated that aloe emodin alleviated RIHD through inhibition of NDP52‐induced mitophagy via repressing SH3GLB1‐mediated mitoROS accumulation. Mechanically, aloe emodin inhibited phosphorylated glycogen synthase kinase 3B (p‐GSK3B) transcription into the nucleus to repress the interaction of prostaglandin G/H synthase 2 (PTGS2) with the SH3 domain of SH3GLB1, thereby reducing the dysfunction of mitochondrial complexes. Also, we confirmed that protein disulfide‐isomerase (P4HB) was a novel target for lactylation at K311 amino acid, the inhibition of P4HB lactylation by aloe emodin could ameliorate cardiac fibrosis of RIHD by stabilizing aspartate aminotransferase (GOT2)‐mediated kynurenine metabolism.

## 2 Results

### 2.1 Aloe Emodin Alleviates Radiation‐Induced Heart Damage by Inhibiting Inflammatory Cascade

**Figure 1:**
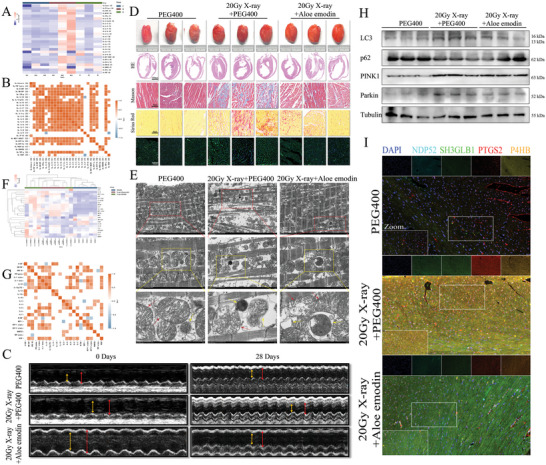
Aloe emodin alleviates radiation‐induced heart damage by inhibiting inflammatory cascade. A) Heatmap assay for 27 inflammatory factors in human serum samples. B) Correlation analysis among biological factors for 27 inflammatory factors in human serum samples. C) Echocardiography detection of rats in 0‐day or 28‐day after X‐ray or aloe emodin treatment. D) Morphological detection and staining evaluation of rat heart, including HE staining, Masson staining, Sirius red staining, and WGA fluorescence. Scale bar, 1000 µm and 40 µm. E) TEM analysis of cardiac tissue of rat heart, red arrow for mitochondria, yellow arrow for autophagic vesicle or autophagosome. Scale bar, 5 µm, 2 µm, and 500nm. F) Heatmap assay for 23 inflammatory factors in rat serum samples. G) Correlation analysis among biological factors for 23 inflammatory factors in rat serum samples. H) Mitophagy‐related protein levels in rat heart tissues, including LC3, p62, PINK1, and Parkin, were determined using Western blotting. I) Co‐localization among NDP52, SH3GLB1, PTGS2, and P4HB was detected by multi‐color immunofluorescence labeling. Scale bar, 20 µm and 10 µm.

We previously found that mitochondrial dysfunction was association with cardiac injury induced by low‐dose X‐ray radiation.^[^ ^18^ ^]^ To further clarify the intrinsic mechanism of radiation in the damage of mitochondrial function and promotion of mitophagy, we analyzed multiple inflammatory factors in serum samples from three different types of clinical patients. LabExLuminex assay detected that partial factors from 27 inflammatory chemokines, including interleukin‐9 (IL‐9), IL‐10, C‐C motif chemokine 5 (RANTES), and platelet‐derived growth factor subunit B (PDGF‐bb), were upregulated in human serum samples (**Figure** **1**A). However, most inflammatory chemokines in radiotherapy patients for thoracic tumors were lower than non‐radiotherapy patients (Figure 1A). Next, we used single‐factor detection by enzyme‐linked immunosorbent assay (ELISA) assay, which showed that concentrations of cardiac troponin T (cTnT), creatine kinase isoenzymes MB (CK‐MB), and L‐lactate dehydrogenase (LDH) were elevated in radiotherapy patients for thoracic tumors, but significantly decreased in non‐radiotherapy patients, suggesting the damaged role of radiation such as X‐ray in cardiac functions and structure. Furthermore, the upregulation of inflammatory factors, including IL‐1β, IL‐6, and TNF‐α, determined the association between inflammatory response caused by radiotherapy and cardiac injury (Figure S1, Supporting Information). Correlation analysis among biological factors in human serum samples further suggested the linear relationship of inflammatory response in RIHD (Figure 1B; Figure S2A, Supporting Information). Considering this, whether a suitable chemical compound can be found to alleviate RIHD by inhibiting inflammatory response, and potential substrate targets and regulatory mechanisms of the chemical compound should be investigated.

Randomly selected rats were divided into three groups with distinct treatments for 28 days, including PEG400, 20 Gy X‐ray+PEG400, and 20 Gy X‐ray+aloe emodin (Figure S3, Supporting Information). Although on the 22nd day, the weight of all rats instantly decreased, there was no significant difference among the three groups, suggesting that it may be due to the animals’ collective social behavior or poor tolerance to Alzet Ostomic pumps (Figure S2B, Supporting Information). Both of heart weight (HW)/body weight (BW) ratio and HW/tibia length (TL) ration were increased after management with 20 Gy X‐ray, but reduction in aloe emodin pretreatment. Echocardiography detection found that aloe emodin treatment significantly improved cardiac functions to 20 Gy X‐ray, including LVIDs, LVIDd, LVPWs, LVPWd, and LVPW% (Figure 1C; Figure S4A, Supporting Information). Moreover, the downregulation of LVPW%, EF%, and FS% induced by radiation was partially functionality restored by aloe emodin, as well as to IVS%, suggesting the protective role of aloe emodin in RIHD (Figure S4B, Supporting Information). Similarly, we used LabExLuminex assay and found that downregulation of multiple factors from 23 inflammatory chemokines indicated the negative association between aloe emodin with RIHD, indicating the strongest association between inflammatory cascade and cardiac injury induced by X‐ray (Figure 1F,G; Figure S5, Supporting Information). ELISA assay detected that high serum concentrations of CK‐MB and LDH induced by 20 Gy X‐ray were downregulated, and also inflammatory factors, such as IL‐1β, IL‐6, and TNFα, were reduced with aloe emodin treatment (Figure S6, Supporting Information). These results provide a preliminary explanation that the main cause of radiation‐induced damage to the structure and function of heart may be induced by the severe inflammatory response within cardiomyocytes.

A morphology study demonstrated the protective effect of aloe emodin on the radiation‐induced cardiac injury. Both left ventricle (LV) wall thickness and cardiomyocyte cross‐sectional area among the three groups were not significantly different by hematoxylin‐eosin (HE) staining and wheat germ agglutimin (WGA) staining, which was contradicted by the previous results in Figure 1C. However, aloe emodin significantly decreased the fibrosis ratio and inhibited the accumulation of collagen in cardiomyocytes, detected by Masson staining and Sirius red assay (Figure 1D; Figure S7A, Supporting Information). Positive expressions of NDP52, SH3GLB1, PTGS2, and P4HB were decreased and also mitophagy‐related protein levels, including LC3, p62, PINK1, and Parkin were downregulated by aloe emodin treatment, suggesting aloe emodin may alleviate RIHD through inhibition of P4HB/PTGS2/SH3GLB1/NDP52 axis‐induced mitophagy (Figure 1Q; Figures S8–S10, Supporting Information). Previous results have reported that NDP52‐induced mitophagy plays a crucial role in low‐dose X‐ray‐mediated cardiac injury.^[^ ^11^ ^]^ Thus, we further found that aloe emodin suppressed mitophagy activation induced by 20 Gy X‐ray by reducing mitochondrial length, mitochondrial area, and area ratio of mitochondrial to nuclear (Figure 1E; Figure S11, Supporting Information). Finally, multiple fluorescence labeling experiments showed that aloe emodin not only downregulated fluorescence intensity of NDP52, SH3GLB1, PTGS2, and P4HB, but inhibited their co‐localization in cardiomyocyte provoked by 20 Gy X‐ray (Figure 1I).

Overall, our results suggest that aloe emodin alleviates RIHD through inhibiting mitophagy activation, in which pathophysiological process may be achieved by reducing the severe inflammatory response induced by the P4HB/PTGS2/SH3GLB1/NDP52 axis.

### 2.2 Aloe Emodin Reduces SH3GLB1‐Mediated mitoROS Accumulation to Inhibit NDP52‐Induced Mitophagy for RIHD Protection

**Figure 2:**
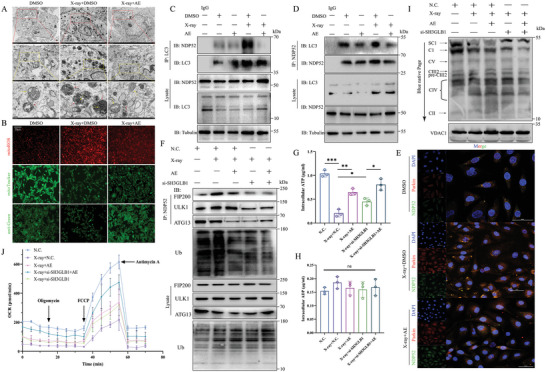
Aloe emodin reduces SH3GLB1‐mediated mitoROS accumulation to inhibit NDP52‐induced mitophagy for RIHD protection. A) TEM analysis of H9C2 cells after X‐ray or aloe emodin treatment, yellow arrow for mitochondria, red arrow for autophagic vesicle or autophagosome. Scale bar, 5 µm, 2 µm, and 500 nm. B) Mitochondrial ROS, mitochondrial morphology, and cardiac cytoskeleton and morphology were detected by mtSOX (DOJinDO, MT14), mitoTracker Green (Beyotime, C1048), and actin‐Tracker Green‐488 (Beyotime, C2201S), respectively. Scale bar, 20 µm. C,D) Co‐immunoprecipitation assay of interaction between LC3 and NDP52. E) Co‐localization between NDP52 and Parkin observing by confocal microscopy. Scale bar, 50 µm. F) Knockdown of SH3GLB1 combined with aloe emodin inhibited the interaction between NDP52 and FIP200, ULK1, or ATG13, detected by Western blotting. G,H) Intracellular concentration of ATP and ADP were analyzed by using ATP assay kit (Beyotime, S0026) and ADP assay kit (Abcam, ab83359). I) Blue native page (Beyotime, P0545S) for Western blotting detection of mitochondrial complexes, including complex I, II, III, IV, and V, using a mixture of five antibodies, after mitochondrial dissociation. J) Partial restoration of mitochondrial oxidative respiratory function under SH3GLB1 deletion combined with aloe emodin, using OCR detection. Quantification data are shown as mean ± SEM. All data were obtained from three independent experiments. *p*‐Values were calculated by unpaired two‐tailed Student's *t*‐test or one‐way ANOVA. *p < 0.05; **p < 0.01; ***p < 0.001; ns: not significant.

Extensive cardiac inflammatory infiltration and structural damage are the main pathological features of RIHD.^[^ ^19^ ^]^ However, multiple studies have revealed that mitophagy, as a “double‐edged sword”, was considered as a defender in inflammatory cascade in cardiac injury.^[^ ^20^ ^]^ To clarify the role of mitophagy in RIDH and elucidate the specific mechanisms between inflammatory response and mitophagy, especially the relationship between SH3GLB1 and NDP52, we first found aloe emodin suppressed mitophagy activation by reducing mitochondrial length, mitochondrial area, and area ratio of mitochondrial to nuclear in vitro (**Figures** **2**A, S12, Supporting Information). Subsequently, we found in the sequence that aloe emodin reduced the fluorescence intensity of mitoROS, but increased in mitoTracker and actin‐Green, suggesting aloe emodin inhibited mitoROS accumulation to restore the mitochondrial functions and structure (Figure 2B; Figure S13, Supporting Information). Furthermore, the protein levels of LC3, PINK1, Parkin, and cardiac injury‐related protein BNP and cTnT were downregulated under aloe emodin treatment. Consistently, both protein levels of NDP52 and SH3GLB1 were reduced in aloe emodin treatment compared to the X‐ray group, whereas p62 expression was significantly elevated, meaning aloe emodin may have a protective role in RIHD through SH3GLB1/NDP52‐mediated mitophagy (Figures S14 and S15, Supporting Information). These results are basically consistent with in vivo experiments, indicating the successful construction of a cell model and confirming that in vitro experiments can provide reliable evidence for the precise mechanism of aloe emodin in protecting RIHD.

To further investigate whether NDP52 was the key target for aloe emodin inhibiting mitophagy, we used co‐immunoprecipitation (co‐IP assay to confirm the interaction of LC3 and NDP52 in the context of X‐ray or aloe emodin pre‐treatment. We found that X‐ray exposure promoted the interaction of NDP52 to LC3, but partially repressed under aloe emodin treatment. Conversely, the interaction of LC3 to NDP52 was inhibited by aloe emodin, suggesting aloe emodin protected against X‐ray‐induced cardiac injury through NDP52‐mediated mitophagy (Figure 2C,D). Research has demonstrated that NDP52 was a key transduction factor for mitophagy activation through Parkin‐mediated ubiquitination.^[^ ^10^, ^21^, ^22^ ^]^ Thus, we used laser‐confocal to find that X‐ray significantly promoted the co‐localization between NDP52 and Parkin, while inhibited by aloe emodin treatment (Figure 2E).

Under physiological conditions, the interaction among NDP52, TAX1BP1, and FIP200 recruits the ULK1 complex, which stabilizes the initiation of autophagosome formation. In other words, the capability of NDP52 to mitophagy activation relies on TBK1‐mediated interaction of FIP200 to ULK1.^[^ ^23^, ^24^ ^]^ To investigate the relationship between SH3GLB1 and NDP52, we should elucidate the interaction of NDP52 with ULK1 or FIP200 in the deletion of SH3GLB1 expression. After knock‐down of the expression of SH3GLB1 in H9C2 cells, we found the binding of NDP52 to FIP200 was significantly decreased, and also the interaction between NDP52 and ULK1 or ATG13 was downregulated in the context of aloe emodin treatment. Surprisingly, SH3GLB1 deficiency combined with aloe emodin remarkably inhibited the binding of NDP52 to ubiquitin, suggesting SH3GLB1 plays a key role in NDP52‐mediated mitophagy activation through the PINK1/Parkin pathway (Figure 2F). These results preliminarily indicated that aloe emodin inhibited NDP52‐induced mitophagy through downregulating PINK1/Parkin signaling via reduction of SH3GLB1‐mediated mitoROS accumulation.

To further elucidate the role of aloe emodin in protecting mitochondrial morphology and restoring mitochondrial functions through inhibition of SH3GLB1‐mediated mitoROS, we detected mitochondrial oxidative respiratory function and energy metabolism function. The intracellular ATP concentration was significantly reduced with X‐ray treatment, whereas partially elevated in aloe emodin pre‐treatment. Additionally, deficiency of SH3GLB1 and aloe emodin restored ATP levels but no significant changes in ADP concentrations (Figure 2G,H). The possible reason for this was detected by Western blotting, the protein levels of mitochondrial complex I, III, and IV/V were partially upregulated, however, the mitochondrial complex II was not significantly altered (Figure 2I; Figure S16, Supporting Information). Moreover, oxygen consumption rate (OCR) detection determined that aloe emodin combined with SH3GLB1 deletion confronted against X‐ray‐induced low utilization rate of oxygen in damaged mitochondrial, suggesting aloe emodin may inhibit mitoROS accumulation to restore the mitochondrial oxidative respiratory function through downregulating SH3GLB1 expression (Figure 2J).

Overall, these results indicate that aloe emodin promotes mitochondrial oxidative respiratory function recovery by downregulating SH3GLB1‐mediated mitoROS accumulation, thereby inhibiting PINK1/Parkin signaling to reduce NDP52‐induced mitophagy, which contributes to protect against RIHD.

### 2.3 Inhibition of PTGS2 Binding to SH3GLB1 is the Key Pathophysiological Process of Aloe Emodin in Repressing NDP52‐Induced Mitophagy

**Figure 3:**
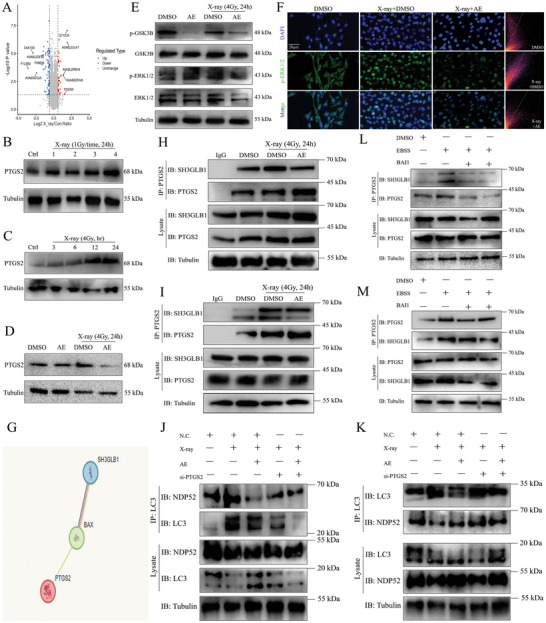
Inhibition of PTGS2 binding to SH3GLB1 is the key pathophysiological process of aloe emodin in repressing NDP52‐induced mitophagy. A) Differential protein omics analysis of upregulation of PTGS2 (P35355) and enrichment of PTGS2 in cardiovascular disease and positive regulation of activation cascade by Volcano plot. B,C) Upregulation of PTGS2 level by X‐ray in a dose‐ and time‐dependent manner was determined using Western blotting. D) Downregulation of PTGS2 level by aloe emodin treatment was determined using Western blotting. E) Downregulation of p‐GSK3B and no significant alteration of t‐GSK3B, p‐ERK1/2, and t‐ERK1/2 by aloe emodin treatment were determined using Western blotting. F) Positive expression and localization of p‐GSK3B by aloe emodin treatment in H9C2 were observed by laser‐confocal microscopy. Scale bar, 20 µm. G) Bioinformation analysis of potential association between PTGS2 AND SH3GLB1 using STRING website. H,I) Co‐immunoprecipitation assay of interaction between PTGS2 and SH3GLB1 after aloe emodin treatment. J,K) Co‐immunoprecipitation assay of interaction between LC3 and NDP52 in the context of PTGS2 deletion combined with aloe emodin. L,M) Co‐immunoprecipitation assay of interaction between PTGS2 and SH3GLB1 after EBSS or BAI1 treatment.

To further investigate the potential mechanism of aloe emodin in regulating SH3GLB1‐mediated mitoROS accumulation, we used the differential proteomic analysis of H9C2 cells treated with X‐ray. PTGS2 (Uniprot ID: P35355) was significantly upregulated by X‐ray treatment compared to the control group, while both KEGG map and GO analysis classified PTGS2 as a key target of acute inflammatory response in cardiovascular disease (**Figure** **3**A; Figure S17, Supporting Information). To further verify whether PTGS2 expression differences in differential omics could be regulated by aloe emodin, results from Western blotting showed that protein level of PTGS2 was upregulated by X‐ray in a time‐ and dose‐dependent manner (Figure 3B,C and Figure S18A,B, Supporting Information). Subsequently, aloe emodin treatment reduced PTGS2, which further supplemented the PTGS2 protein level by aloe emodin treatment that was not detected in differential omics (Figure 3D; Figure S18C, Supporting Information). Thus, PTGS2 was preliminary confirmed to be a key player involved in cardiac injury induced by X‐ray, however, upstream regulatory targets or mediated mechanism of PTGS2 upregulation is still unclear. Numerous studies have regarded both p‐ERK1/2 and p‐GSK3B as important factors in regulating the expression and regulation of PTGS2, and their transcription into the nucleus can effectively bind to the promoter of PTGS2 and further regulate downstream inflammatory responses mediated by PTGS2.^[^ ^25^, ^26^ ^]^ To further test the hypothesis that both p‐ERK1/2 and p‐GSK3B are involved in regulatory expression of PTGS2 induced by aloe emodin, the results from Western blotting revealed that the protein levels of p‐ERK1/2, t‐ERK1/2 and t‐GSK3B were no alteration, but X‐ray‐promoted high expression of p‐GSK3B was significantly reduced by aloe emodin (Figure 3E; Figure S19, Supporting Information). Furthermore, dual treatment with X‐ray and aloe emodin barely changed the fluorescence intensity of p‐GSK3B, but the co‐localization of p‐GSK3B and nucleus was significantly inhibited by aloe emodin (Figure 3F; Figure S20, Supporting Information). These results suggest that aloe emodin downregulates PTGS2 expression through inhibition of p‐GSK3B transcription into the nucleus, which might be involved in X‐ray‐induced inflammatory responses and RIHD.

Receptor tyrosine‐protein kinase erbB‐2 (ERBB2), encoded by the *HER2* oncogene, is a transmembrane tyrosine kinase receptor involved in promoting cell cycle progression, cell proliferation, differentiation and survival, and angiogenesis.^[^ ^27^ ^]^ Research has shown that ERBB2 activation elicits the GSK3B phosphorylation through the MEMO1/RHOA/DIAPH1 signaling pathway.^[^ ^28^ ^]^ Additionally, ERBB2 can assist p‐GSK3B to associate with the promoter of PTGS2 and activate its transcription.^[^ ^29^ ^]^ To further test the possibility of induced expression of PTGS2 by p‐GSK3B, we performed co‐IP after overexpressing Flag‐PTGS2 and HA‐ERBB2 in HEK‐293T cells, which showed that a large amount of Flag tag was pulled down in HA tag in the context of X‐ray, but decreased in aloe emodin treatment, indicating that p‐GSK3B‐induced PTGS2 expression assisted by ERBB2 was inhibited by aloe emodin (Figure S21A,B, Supporting Information). Furthermore, the X‐ray‐provoked interaction of PTGS2 and SH3GLB1 was significantly repressed by aloe emodin, suggesting PTGS2 was involved in SH3GLB1‐mediated mitoROS accumulation (Figure 3H,I). However, the potential relationship between PTGS2 and SH3GLB1 is unclear and the possible regulatory mechanism of PTGS2 in mediating SH3GLB1‐induced mitoROS accumulation should be investigated. Further bioinformatics analysis revealed a possible interaction between PTGS2 and SH3GLB1 via BAX (Figure 3G). Then, we performed co‐IP to pull down PTGS2 or SH3GLB1, and found that the binding of SH3GLB1 to PTGS2 promoted by X‐ray was significantly inhibited by aloe emodin (Figure 3H,I). To further validate the role of PTGS2 as a key mediator of the NDP52‐induced mitophagy, we knocked down PTGS2 expression and performed co‐IP. The results indicated that PTGS2 deficiency suppressed X‐ray‐induced mitophagy through inhibition of LC3 and NDP52, additionally, as expected, dual treatment with aloe emodin and PTGS2 deletion significantly inhibited interaction between NDP52 and LC3 (Figure 3J,K). These results preliminarily indicated that aloe emodin reduced NDP52‐induced mitophagy by inhibition of binding SH3GLB1 to PTGS2, in which the reduction in the interaction of SH3GLB1 and PTGS2 relied on the repression of p‐GSK3B transcription into the nucleus by aloe emodin.

Under stress conditions, the conformation of BAX undergoes changes, leading to the release of cytochrome *C* from mitochondria, which is involved in inflammatory response‐mediated mitophagy and cell apoptosis.^[^ ^30^ ^]^ Because non‐active BAX mainly exists in the cytoplasm and has no effect on mitochondrial dysfunction and mitophagy activation, whereas active BAX, which is concentrated in outer mitochondrial membrane, participates in the fusion of autophagic vesicles with damaged mitochondrial to form autophagosomes, we pre‐treated H9C2 cells with BAI1, a selective and allosteric inhibitor of BAX, and autophagic inducer earle's balanced salt solution (EBSS), respectively.^[^ ^31^ ^]^ From the results by laser‐confocal microscopy, we found that EBSS promoted the co‐localization of PTGS2 to mitoTracker, but inhibition of BAX by BAI1 significantly reduced the binding of PTGS2 and mitochondria (Figure S22, Supporting Information). In agreement with the confocal result, the interaction between SH3GLB1 and PTGS2 induced by EBSS was also inhibited by BAI1, suggesting PTGS2 was involved in mitophagy activation through SH3GLB1/NDP52 signaling (Figure 3L,M).

Collectively, PTGS2 is confirmed to be a critical mediator in inducing mitophagy through the regulation of inflammatory response. We further indicate that aloe emodin inhibits the interaction of PTGS2 with SH3GLB1 by repressing transcription of p‐GSK3B into the nucleus, and decreases mitoROS accumulation, thus, resulting in the reduction of NDP52‐induced mitophagy.

### 2.4 SH3 Domain Is Required For Interaction of PTGS2 with SH3GLB1 and Plays a Key Role in Mitophagy

**Figure 4:**
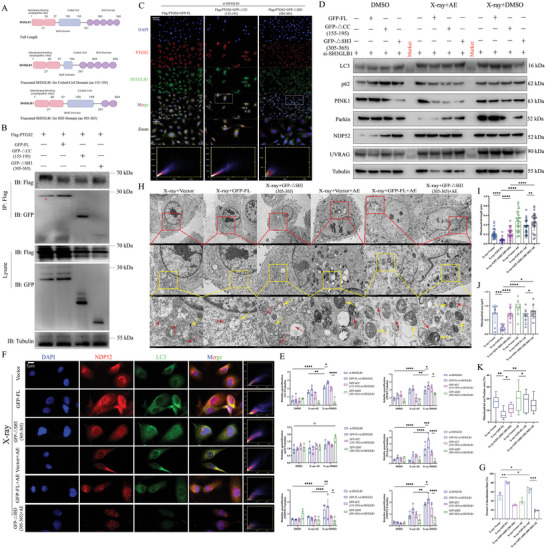
SH3 domain is required for the interaction of PTGS2 with SH3GLB1, playing a key role in mitophagy. A) Pattern diagram for constructing plasmids, including GFP‐SH3GLB1‐full length, GFP‐SH3GLB1‐△CC (aa155‐195) and GFP‐SH3GLB1‐△SH3 (aa305‐365). B) Co‐immunoprecipitation assay of interaction between PTGS2 and SH3GLB1 in HEK‐293T cells after transfection of GFP‐FL, GFP‐△CC, and GFP‐△SH3. Red marker (*) in the left lane indicates inevitable non‐specific binding. C) Co‐localization of PTGS2 and SH3GLB1 after transfection of GFP‐FL, GFP‐△CC, and GFP‐△SH3 in the context of endogenous SH3GLB1 deletion in H9C2 cells. Scale bar, 10µm. D,E) Protein levels of NDP52, UVRAG, LC3, p62, PINK1, and Parkin were determined using Western blotting. F,G) Significant inhibition of co‐localization between LC3 and NDP52 in H9C2 cells with treatment of aloe emodin and GFP‐△SH3. Scale bar, 5 µm. H–K) TEM analysis of H9C2 cells after treatment of aloe emodin and GFP‐△SH3, yellow arrow for mitochondria, red arrow for autophagic vesicle or autophagosome. Scale bar, 5 µm, 2 µm, and 500nm. Quantification data are shown as mean ± SEM. All data were obtained from three independent experiments. *p*‐Values were calculated by unpaired two‐tailed Student's *t*‐test or one‐way ANOVA. *p < 0.05; **p < 0.01; ***p < 0.001; ****p < 0.0001; ns: not significant.

To further investigate the potential mechanism of PTGS2 interacting with SH3GLB1, we have analyzed the structural domains listed in GO analysis. However, certain domains that can effectively combine PTGS2 do not exist in the present omics data, but it suggests that SH3GLB1 may be a novel target for binding to PTGS2, and the structural domain within SH3GLB1 needs further investigation (Figure S23, Supporting Information). SH3 domain, a crucial structure in SH3GLB1, is a small protein interaction module composed of a β‐sandwich consisting of five strands connected by three loops and a short 3_10_ helix.^[^ ^32^ ^]^ According to the structural characteristics of the SH3 domain and its specific position on the SH3GLB1 protein, therefore, we constructed a full‐length expression plasmid of SH3GLB1 (GFP‐SH3GLB1‐FL) and two truncated expression plasmids of SH3GLB1, including deletion of aa155‐195 (GFP‐SH3GLB1‐△CC) and deletion of aa305‐365 (GFP‐SH3GLB1‐△SH3) (**Figure** **4**A). To clarify the specific binding area of PTGS2 and SH3GLB1, we performed co‐IP after overexpressing Flag‐PTGS2 and GFP‐SH3GLB1 in HEK‐293T cells. We found that the interaction between PTGS2 and SH3GLB1 was significantly inhibited under the condition of GFP‐△SH3 deletion, but no change either in GFP‐FL nor GFP‐△CC (Figure 4B). Furthermore, we knocked down SH3GLB1 expression by small interfering RNA, and laser confocal microscopy showed that PTGS2 significantly bond to both GFP‐FL and GFP‐△CC respectively, whereas inhibition in GFP‐△SH3 condition (Figure 4C; Figure S24, Supporting Information).

To further test the role of the SH3 domain in inducing mitophagy, we performed overexpression of GFP‐FL, GFP‐△CC, or GFP‐△SH3 after deletion of SH3GLB1 in H9C2 cells. Results from immunofluorescence showed that GFP‐FL promoted mitoROS accumulation, upregulation of actin‐Green, and mitoTracker upregulated by X‐ray, but they were significantly inhibited by aloe emodin. As expected, GFP‐△SH3 not only affected the promoting effect of X‐ray in dysfunctional mitochondria, but also was synergistic with aloe emodin to inhibit the mitoROS accumulation, damage of cardiomyocyte structure, and mitochondrial functions (Figure S25A,B, Supporting Information). Western blotting showed that protein levels of LC3, PINK1, and Parkin upregulated by X‐ray were significantly decreased in the context of dual treatment with X‐ray and GFP‐FL or GFP‐△CC, as well as in NDP52 and UV radiation resistance‐associated gene protein (UVRAG) (Figure 4D,E). However, protein levels of LC3, PINK1, Parkin, NDP52, and UVRAG were downregulated accompanied by the expression of GFP‐△SH3. Aloe emodin accelerated the reduction of their expression, but no change in p62 (Figure 4D,E). These results revealed that the SH3 domain on SH3GLB1 plays a key role in NDP52‐mediated mitophagy activation through binding with PTGS2. Similarly, dual treatment with aloe emodin and GFP‐△SH3 significantly inhibited the binding of LC3 to NDP52 compared to the aloe emodin+GFP‐FL group in the context of X‐ray, and also reduced in co‐localization of LC3 and NDP52 compared to X‐ray+GFP‐△SH3 (Figure 4F,G). To further determine the specific role of the SH3 domain in stabilizing mitochondrial morphology and inducing mitophagy, TEM was used and it was found that GFP‐△SH3 partially restored mitochondrial length and area, inhibited fusion of autophagic vesicles and damaged mitochondria, leading to reduced mitophagy activation. Co‐treatment with aloe emodin and GFP‐△SH3 confronted the accumulation of autophagic vesicles and the formation of autophagosomes induced by X‐ray (Figure 4H–K). These results showed that the interaction of the SH3 domain with PTGS2 induced SH3GLB1‐mediated mitochondrial dysfunction, which was involved in the inhibition of aloe emodin in NDP52‐induced mitophagy.

Collectively, we confirm that aloe emodin inhibits the interaction of PTGS2 and SH3 domain on SH3GLB1 to reduce mitoROS accumulation, and suppresses NDP52‐induced mitophagy activation, which alleviates X‐ray‐provoked RIHD development.

### 2.5 Aloe Emodin Inhibits K311‐Linked Lactylation of P4HB Induced by X‐Ray to Reduce Mitophagy

**Figure 5:**
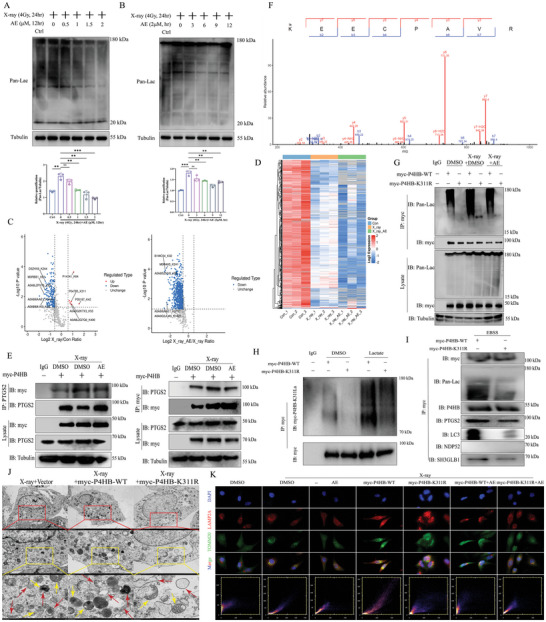
Aloe emodin inhibits K311‐linked lactylation of P4HB induced by X‐ray to reduce mitophagy. A, B) Downregulation of protein levels of Pan‐Lac by aloe emodin in dose‐ and time‐dependent manner, determined using Western blotting. C,D) Lactylation omics of 4D label‐free analysis of upregulation and modification for P4HB (P04785), and expression of P4HB was inhibited by aloe emodin by Heatmap. E) Co‐immunoprecipitation assay of interaction between P4HB and PTGS2. F) LC–MS/MS analysis of lysine 311 (K311) was the key site for interaction and lactylation of P4HB. R) K311R mutation plasmid significantly blocked binding of P4HB to Pan‐Lac detecting by co‐immunoprecipitation. H) K311R mutation plasmid inhibited binding of P4HB and K311La in HEK‐293T cells incubating with lactate. I) K311R mutation repressed interaction of P4HBk with PTGS2, LC3, NDP52, SH3GLB1, and Pan‐Lac under EBSS treatment. J) TEM analysis of H9C2 cells treated with K311R mutation plasmid, yellow arrow for mitochondria, red arrow for autophagic vesicle or autophagosome. Scale bar, 5 µm, 2 µm, and 500 nm. K) Co‐localization of TOMM20 and LAMP2A in the context of aloe emodin treatment with K311R. Scale bar, 5 µm. Quantification data are shown as mean ± SEM. All data were obtained from three independent experiments. *p*‐Values were calculated by unpaired two‐tailed Student's *t*‐test or one‐way ANOVA. *p < 0.05; **p < 0.01; ***p < 0.001; ****p < 0.0001; ns: not significant.

Clinical evidence has demonstrated that high serum levels of lactate are prevalent in heart failure patients and are associated with mobility and mortality of cardiac injury patients.^[^ ^33^ ^]^ Lactylation of substrate targets is normally accompanied by the appearance of the inflammatory response, whether cardiac ROS accumulation induced by X‐ray can promote potential lactylation in proteins needs to be clarified. In the present study, we first found that the protein level of Pan‐Lactate was upregulated in X‐ray treatment in a dose‐ and time‐dependent manner (Figure S26A,B, Supporting Information). To further determine whether aloe emodin could ameliorate the molecular level of lactate, different concentrations of aloe emodin were incubated in H9C2 cells. After X‐ray, the protein level of Pan‐Lactate was gradually downregulated in a dose‐ and time‐dependent manner by aloe emodin (**Figure** **5**A,B).

To confirm the precise targets that can be lactylated, H9C2 cells treated with X‐ray or aloe emodin were divided into three groups, and subjected to lactylation modification omics of 4D label‐free and mass spectrometry analysis by PTM Bio company. As shown in Figure 5C, multiple proteins, such as Ca3 (Uniprot ID: P14141), P4HB (Uniprot ID: P04785), and EeF2 (Uniprot ID: P05197) were upregulated and could be lactylated (Figure 5C). Furthermore, we used molecular docking software to evaluate the affinity of aloe emodin and P4HB. Results from Autodock Vina, aloe emodin was electrostatically bound to P4HB through visible hydrogen bonds. Aloe emodin successfully occupied the hydrophobic pockets of P4HB. Moreover, low binding energy with −7.092 kcal mol^−1^ indicated the highly stable binding of P4HB and aloe emodin (Figure S27A, Supporting Information). However, the possible regulatory target of aloe emodin in inhibiting lactate accumulation and lactylation was unclear. To verify the direct relationship between aloe emodin and P4HB, we synthesized and constructed aloe emodin labeled with biotin using organic chemistry methods, namely AE‐biotin. By using Co‐IP and streptavidin incubation, it showed that X‐ray promoted the interaction between biotin and P4HB, suggesting the direct binding relationship of aloe emodin to P4HB (Figure S27B–D, Supporting Information).

Given the provided gene features, we conducted a cross‐comparison using heat map analysis and KEGG map, and identified P4HB as a potential lactylated protein involved in X‐ray‐induced RIHD (Figure 5D; Figure S28, Supporting Information). In parallel, treatment with X‐ray increased the protein levels of P4HB in a dose‐ and time‐dependent manner, whereas aloe emodin reduced P4HB expression in H9C2 cells (Figure S29A–C, Supporting Information). To further determine whether P4HB could regulate PTGS2‐mediated mitophagy activation through the SH3GLB1/NDP52 axis, we overexpressed P4HB with myc tag within HEK‐293T cells. Co‐IP assay showed that X‐ray promoted interaction between myc and PTGS2, while aloe emodin inhibited the binding of PTGS2 to P4HB (Figure 5E). The hypothesis is that lactate may play a critical role in the interaction of P4HB and PTGS2, thus we further used small interfering RNA to knock down the key rate‐limiting enzyme for lactate production, LDHA, and found that the deletion of LDHA had no effect in the interaction of P4HB with Pan‐Lac induced by X‐ray, suggesting lactylation of P4HB is independent on LDHA expression (Figure S30A,B, Supporting Information). However, we further used an inhibitor of lactate dehydrogenase, LDHi, to incubate H9C2 cells after overexpression myc‐P4HB, and it showed the interaction of P4HB and Pan‐Lac induced by X‐ray was significantly inhibited under dual treatment with LDHi and aloe emodin (Figure S31A,B, Supporting Information). These results indicate that lactylation of P4HB is a crucial pathophysiological process for P4HB binding to PTGS2 to regulate RIHD.

Although P4HB has been defined as a novel target for lactylation, the specific modification site of P4HB is unclear, and whether the interaction of lactate molecule with the modification site of P4HB can regulate mitoROS and mitophagy needs to be studied. To determine the precise modification site of P4HB, we have collaborated with PTM Bio company to comprehensively identify and analyze the potential modification sites of P4HB. Liquid chromatograph mass spectrometer (LC/MS) showed that the relative abundance of amino acid at position 311, a lysine residue (K), was significantly excited, with a score of 99.305, suggesting K311 on P4HB may be the key modification site of lactylation (Figure 5F). Our previous findings showed that the P4HB lactylation was the key process for its binding to PTGS2. To further clarify whether the K311 site determines the interaction of PTGS2 with P4HB, we constructed wild‐type plasmids (myc‐P4HB‐WT) and point mutation plasmids (myc‐P4HB‐K311R), respectively. After overexpression myc‐P4HB‐WT or myc‐P4HB‐K311R in HEK‐293T cells, results from laser‐confocal showed that myc‐P4HB‐WT remarkably promoted the interaction of P4HB and PTGS2 upregulation in the context of X‐ray, while P4HB binding to PTGS2 was almost imperceptible after myc‐P4HB‐K311R overexpression (Figure S32, Supporting Information). Although these results indicated that K311 appeared to be a key site determining the binding state of P4HB and PTGS2, it is unknown whether K311 can undergo lactylation and regulate the interaction of P4HB and PTGS2 after K311 lactylation should be investigated.

Given the unclear role of K311 in regulating mitophagy through P4HB lactylation, we overexpressed myc‐P4HB‐WT or myc‐P4HB‐K311R in HEK‐293T cells treated with X‐ray or aloe emodin. We performed co‐IP to reveal that there was no interaction of P4HB with Pan‐Lac after myc‐P4HB‐K311R treatment, either in the context of X‐ray or aloe emodin treatment (Figure 5G). In order to eliminate endogenous interference, especially the false positive interference of metabolites in glycolysis on lactylation, we incubated H9C2 cells with LDHi and subsequently added exogenous lactate. We used co‐IP to find that the binding of P4HB to Pan‐Lac was upregulated by lactate, while it was inhibited by LDHi, suggesting P4HB is a novel substrate target for lactylation (Figure S33A,B, Supporting Information). Moreover, we overexpressed myc‐P4HB‐WT or myc‐P4HB‐K311R in HEK‐293T cells treated with lactate, and used myc to pull down P4HB and incubated with P4HB K311‐sensitive Pan‐lactylation antibody (P4HB‐K311La). Co‐IP assay showed that lactate promoted the interaction between myc‐P4HB‐WT and K311La, however, it barely detected the binding of myc‐P4HB‐K311R to suggesting K311 was a critical amino acid for P4HB lactylation (Figure 5H). These results suggest that K311 lactylation plays a key role in the interaction of P4HB and PTGS2, which may be involved in PTGS2‐mediated mitoROS accumulation and mitophagy activation.

Next, we investigated whether P4HB lactylation could promote the fusion of autophagic vesicles and damaged mitochondria through K311 for NDP52‐induced mitophagy. After incubation with EBSS, the interaction between P4HB and Pan‐Lac, PTGS2, or SH3GLB1 was lower than the myc‐P4HB‐WT group, and also the binding of P4HB to LC3 or NDP52 was decreased (Figure 5I). Compared to the X‐ray group, we found that myc‐P4HB‐WT further provoked autophagosome accumulation, whereas myc‐P4HB‐K311R partially restored mitochondrial morphology and inhibited mitophagy (Figure 5J; Figure S34, Supporting Information). Whether aloe emodin could coordinate with P4HB mutation to inhibit transport of mitochondrial to the lysosome for degradation. Indeed, laser‐confocal microscopy showed that P4HB K311 mutation attenuated the co‐localization between TOMM20 and LAMP2A inhibited by aloe emodin, revealing that K311 was the key site for repression of aloe emodin in mitophagy activation (Figure 5K; Figure S35, Supporting Information). Together, these results suggest that P4HB is a novel protein target for lactylation under physiological or pathological processes, and K311‐linked lactylation on P4HB plays an important role in regulating SH3GLB1‐mediated mitoROS accumulation through interacting with PTGS2, and is also the key target for alleviation of aloe emodin to RIHD.

### 2.6 Aloe Emodin Ameliorates Imbalance of GOT2‐Mediated Kynurenine Metabolism to Alleviate Cardiac Injury

Kynurenine metabolism plays an important role in cardiovascular diseases, particularly linked with cardiac oxidative stress.^[^ ^34^ ^]^ As a central catabolic route of tryptophan, kynurenine leads to mediation of NAD's biosynthesis.^[^ ^35^ ^]^ Kynurenine metabolites, including quinolinic acid (QA) and kynurenine‐3‐monooxygenase (KMO), result in negative impacts on the inflammatory response of cardiomyocytes, including immune infiltration and selective cell death.^[^ ^36^ ^]^ However, the specific role of kynurenine metabolism in RIHD has not been reported, and the mechanisms of kynurenine metabolism involved in lactylation‐induced mitophagy are unclear.

**Figure 6:**
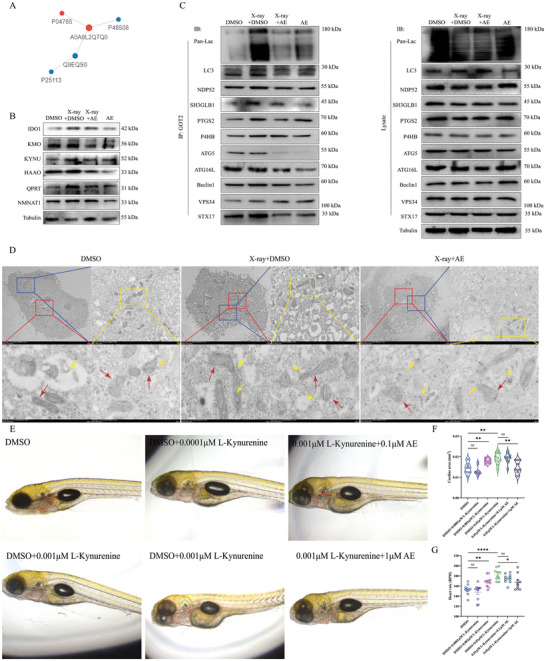
Aloe emodin ameliorates the imbalance of GOT2‐mediated kynurenine metabolism to alleviate cardiac injury. A) Relationship between P4HB and GOT2 (A0A8L2Q7Q0) analyzed by PPI network diagram. B) Alteration of protein levels of kynurenine metabolism under aloe emodin treatment, including IDO1, KMO, KYNU, HAAO, QPRT, and NMNAT1, determined using Western blotting. C) Inhibition of the interaction between GOT2 and P4HB, ATG5, VPS34, or STX17 by aloe emodin treatment, detected by Western blotting. D) Accumulation of GOT2 in damaged mitochondria of H9C2 cells detected by immunocolloidal gold transmission electron microscopy, red arrow for damaged mitochondria and yellow arrow for marking GOT2. Scale bar, 5 µm, 1 µm, and 500 nm. E–G) Morphological detection of zebrafish heart in the context of l‐kynurenine or aloe emodin treatment. Quantification data are shown as mean ± SEM. All data were obtained from three or ten independent experiments. *p*‐Values were calculated by unpaired two‐tailed Student's *t*‐test or one‐way ANOVA. *p < 0.05; **p < 0.01; ***p < 0.001; ****p < 0.0001; ns: not significant.

To investigate the relationship between kynurenine metabolism and the protection of aloe emodin in RIHD, we analyzed the GO data from lactylation modification omics. Our data indicated that 74 potential targets were involved in cellular metabolic processes, and only 1 protein was given into the amino acid transport and metabolism (Figure S36A,B, Supporting Information). We intersected the results from Figure S36 (Supporting Information) for analysis and formed a PPI network diagram, which showed the high potential correlation between P4HB and GOT2 (Uniprot ID: A0A8L2Q7Q0) (**Figure** **6**A). To further clarify the role of aloe emodin in regulating kynurenine metabolism, we detected the key rate‐limiting enzymes in the process of kynurenine metabolism, including IOD1, KYNU, and NMNAT1 (Figure S37, Supporting Information). Results from Western blotting showed that the protein levels of IDO1, KMO, KYNU, HAAO, QPRT, and NMNAT1 were upregulated upon X‐ray treatment, but remained with aloe emodin treatment (Figure 6B; Figure S38, Supporting Information). GOT2 can catalyze L‐kynurenine to kynurenic acid (KA), which is an irreversible transamination.^[^ ^33^ ^]^ As a member of the malate‐aspartate shuttle, GOT2 displays an irreplaceable role in the intracellular NAD(H) redox balance.^[^ ^37^ ^]^ Therefore, we performed IP assay to observe whether aloe emodin could inhibit mitophagy through GOT2 activity, and found that aloe emodin significantly reduced the interaction of GOT2 with lactylation‐related proteins, such as Pan‐Lac and P4HB. Additionally, the binding of GOT2 to mitophagy‐related proteins, including LC3, NDP52, ATG5, and beclin1, was also inhibited by aloe emodin treatment (Figure 6C). These data suggest that GOT2‐mediated kynurenine metabolism may be involved in NDP52‐induced mitophagy.

To further unify the spatial localization and expression changes of GOT2 in the context of X‐ray or aloe emodin treatment, we performed immunocolloidal gold transmission electron microscopy to detect the expression of GOT2 in damaged mitochondria of H9C2 cells. Data showed that X‐ray induced abundant expression of GOT2 in the cytoplasm and promoted accumulation of GOT2 on damaged outer mitochondrial membranes, accompanied by more GOT2 approaching or aggregating towards autolysosomes (Figure 6D). On the contrary, aloe emodin did not significantly reduce the expression of GOT2 in the cytoplasm, but inhibited the localization and aggregation of GOT2 in mitochondrial and autophagosomes (Figure 6D). Furthermore, both SH3GLB1 and lysosomal degradation‐related targets such as ATG16L, VPS34, and STX17 were inhibited to interact with GOT2 by aloe emodin, indicating aloe emodin induced damaged mitochondrial to the lysosome (Figure 6C).

Next, we used ELISA assay or metabonomics entrusted by OEbiotech to detect the serum metabolite levels of kynurenine metabolism in rat and human samples. Ten metabolites from arachidonic acid metabolism, including leukotriene E4, prostaglandin E2, 6‐keto‐prostaglandin F1a, 12‐HETE, 2,3‐dinor prostaglandin E1 and GPCho, prostaglandin D3, 20‐hydroxyeleosatetraenoic acid, 12‐KETE, and 15H‐11,12‐EETA were all downregulated to varying degrees by aloe emodin in rat (Figure S39, Supporting Information). Three metabolites from glycerophospholipid metabolism, including phosphatidylcholine, lyso‐phosphatidylcholine, and 2‐lyso‐phosphatidylcholine, were elevated by aloe emodin treatment, but no changes in 1‐palmitoylphosphatidylcholilne. Conversely, the serum level of l‐serine upregulated by X‐ray was reduced under aloe emodin treatment in rats (Figure S40, Supporting Information). Moreover, three metabolites from tryptophan metabolism, including l‐tryptophan, indoleacetaldehyde, and 5‐hydroxyindoleacetate, were restored by aloe emodin, but significantly decreased in 5‐hydroxykynurenine and N’‐formylkynurenine, compared to 20 Gy X‐ray group (Figure S41, Supporting Information). Almost identical data was observed in human serum samples, 9 metabolites from arachidonic acid metabolism were upregulated in radiotherapy patients for thoracic tumors, but no changes in 12‐HETE. Furthermore, three metabolites, including 12‐HETE, 12‐KETE, and 15H‐11,12‐EETA, had no statistically significant changes (Figure S42, Supporting Information). Additionally, glycerophospholipid metabolism and tryptophan metabolism from human serum samples showed almost identical results to rat serum samples, except for no changes in levels of lyso‐phosphatidylcholine and N’‐formylkynurenine detected in humans (Figures S43 and S44, Supporting Information). These results demonstrate that kynurenine metabolism plays a key role in the inhibition of RIHD by aloe emodin.

Eventually, the kynurenine supplementation experiment further elucidates the protective role of aloe emodin on the cardiac injury. We added different concentrations of l‐kynurenine to the zebrafish culture system after pretreated with 0.1 µm or 1 µm aloe emodin (Figure 6E). Results showed that the cardiac morphology and function of zebrafish were significantly damaged in 0.01 µm l‐kynurenine, while 1 µm aloe emodin partially restored the cardiac function and decreased damaging area (Figure 6F,G). Together, X‐ray disrupts the balance of kynurenine metabolism and activates NDP52‐induced mitophagy, whereas aloe emodin alleviates RIDH development by inhibiting the accumulation of GOT2 on damaged mitochondria and stabilizing kynurenine metabolism.

## 3 Discussion

**Figure 7:**
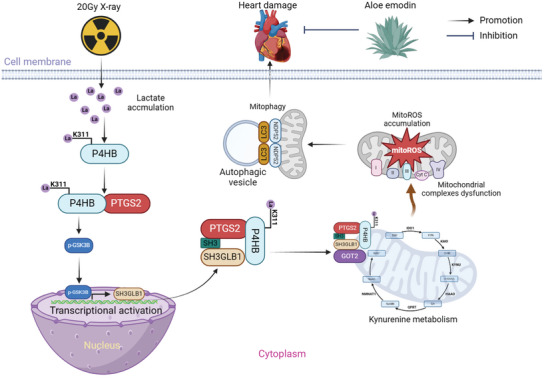
Schematic diagram of regulatory mechanisms of aloe emodin in preventing RIHD through P4HB/PTGS2/SH3GLB1/NDP52 axis.

RIHD is a significant concern in radiotherapy for thoracic tumors. Previous studies have indicated mitochondrial dysfunction and inflammatory response as key contributors to RIHD.^[^ ^11^, ^19^ ^]^ However, the potential mechanisms by which inflammatory response is cascaded are still unclear for clarifying the regulatory frameworks to understand the role of mitophagy activation in provoking cardiac injury. Our previous research has indicated that NDP52‐induced mitophagy responds to inflammation accumulation within cardiomyocytes through the SH3GLB1/PINK1 pathway, thereby exacerbating cardiac injury. Given that, targeting the regulation of NDP52‐induced mitophagy against potential targets of inflammatory response is urgently needed to develop new and effective pharmacological targets for alleviating RIHD. In the present study, we first revealed that aloe emodin treatment in X‐ray‐induced cardiac injury rats could significantly reduce the severe inflammatory response, restore cardiac functions, and ameliorate cardiac fibrosis via inhibiting mitophagy activation. Furthermore, we demonstrated that aloe emodin inhibits the interaction of PTGS2 with the SH3 domain on SH3GLB1 by repressing the transcription of p‐GSK3B into the nucleus, which reduces mitoROS accumulation. The mechanism involves X‐ray‐induced high abundance of lactate in cardiomyocytes and lactylation of P4HB at K311 promotes mitoROS accumulation and mitophagy activation through the interaction of P4HB and PTGS2, which contributes to the RIHD development. Both clinical data and animal detection showed that imbalanced kynurenine metabolism mediated by GOT2 impeded cardiac functions, and aloe emodin promoted damaged mitochondrial for lysosomal degradation by the interaction of GOT2 and LAMP2A to alleviate cardiac dysfunctions. Collectively, our findings indicated that aloe emodin may be a potential novel agent for the treatment of RIHD (**Figure** **7**).

Although mitophagy plays a double‐edged sword role in multiple cardiovascular diseases, our previous study demonstrated that mitophagy, especially mediation by NDP52, aggravates cardiac dysfunction, and damages cardiac structure, which accelerates RIHD.^[^ ^11^, ^38^ ^]^ Therefore, targeting initiation and activation of mitophagy to explore the RIHD pathogenesis and study potential therapeutic drugs has become an important direction in the field of radiation injury. Aloe emodin is a natural anthraquinone derivative and is mainly extracted from aloe vera.^[^ ^12^ ^]^ Multiple pharmacological effects of aloe emodin were demonstrated by emerging evidence, including anti‐inflammatory, cardioprotective, and neuroprotective activities.^[^ ^14^ ^]^ However, whether aloe emodin can alleviate cardiac injury induced by radiation through inhibition of inflammatory response and mitophagy inactivation remains unclear. Our clinical data by LabExLuminex assay showed that high serum levels of pro‐inflammatory factors were significantly upregulated in radiotherapy patients for thoracic tumors, but low in non‐radiotherapy patients. Mitochondrial morphology was observed by TEM and showed multiple autophagic vesicles fusing with damaged mitochondria were inhibited by aloe emodin. IHC staining and multiple fluorescence labeling experiments displayed downregulation of NDP52, SH3GLB1, PTGS2, and P4HB under aloe emodin treatment. These results preliminarily indicated aloe emodin alleviated RIHD development through inhibition of inflammatory cascade and mitophagy activation potentially by the P4HB/PTGS2/SH3GLB1/NDP52 axis.

Accumulated findings suggest that inflammatory response and mitochondrial dysfunction are not only the primary pathophysiological features of cardiac injury, but also the critical targets to clarify the pathogenesis of RIHD and elucidate the prevention and treatment drugs of RIHD.^[^ ^3^, ^4^ ^]^ SH3GLB1, also known as endophilin B1 or Bif‐1, is a member of the endophilin family proteins, and is identified to be involved in mitochondrial fission through interacting with Beclin1 via UVRAG.^[^ ^39^ ^]^ Emerging evidence showed that SH3GLB1 deficiency in melanoma cells exhibited sensitivity to irradiation‐induced cell death, and had foundational autophagic activities.^[^ ^40^ ^]^ Therefore, we first found that the protective role of aloe emodin in inhibiting cardiac injury through SH3GLB1. Then, the interaction of NDP52 with ULK1 or FIP200 was reduced in the context of SH3GLB1 deletion with aloe emodin treatment. Furthermore, aloe emodin combined with SH3GLB1 deficiency repressed the binding of NDP52 to ubiquitin, indicating that SH3GLB1 is a key target for aloe emodin inactivating NDP52‐mediated mitophagy. Research has demonstrated that SH3GLB1 could regulate the activation of PIK3C3 through binding to Beclin1 via UVRAG, which was involved in the formation of autophagosomes.^[^ ^41^ ^]^ Additionally, OCR detection exhibited that SH3GLB1 deletion was synergistic with aloe emodin to restore the low utilization rate of oxygen in damaged mitochondria. However, the specific mechanism of aloe emodin in regulating the activation of mitochondria oxidative respiratory chain was unclear. The potential mechanism may be involved in aloe emodin transferring and supplementing electrons into the mitochondrial complexes by promoting the interaction between SH3GLB1 and the mitochondrial complex I. In addition, DNA double‐strand breaks (DSB) were demonstrated to be involved in triggering cellular DNA damage response, which was the most lethal lesion. Whether the DBS mechanism involved in p‐GSK3B‐mediated transcriptional expression of SH3GLB1, and the relationship between DBS and lactylation should be clarified.

PTGS2 is a member of cyclooxygenases that catalyzes the first rate‐limiting step in prostaglandins synthesis from arachidonic acid. PTGS2 is expressed in multiple cancer cells for resistance to radiotherapy.^[^ ^42^ ^]^ Kellogg et al. have reported that the significant interaction of PTGS2 with the promoter region of NF‐κB upregulated inflammatory factors expression, which promoted the development of diabetic peripheral neuropathy.^[^ ^43^ ^]^ Furthermore, other studies revealed that high protein levels of PTGS2 in monocytes accelerated advanced glycated end‐product deposition, and exacerbated proinflammatory signaling driven by hyperglycemia, which promoted the process of type 1 diabetes.^[^ ^44^, ^45^ ^]^ Both differential proteomic analysis and Western blotting showed that PTGS2 was enriched in cardiovascular diseases, regulation of protein activation cascade, positive regulation of fibroblast, and acute inflammatory response. Previous findings indicated that ERBB2, a transmembrane tyrosine kinase receptor, elicits the p‐GSK3B expression to associate with the promoter of PTGS2 and activates its transcription.^[^ ^29^ ^]^ We found that the interaction of PTGS2 with ERBB2 was decreased under aloe emodin treatment, resulting in the inhibition of SH3GLB1‐mediated mitoROS accumulation. These results indicate that aloe emodin inhibits the interaction of PTGS2 with SH3GLB1 by repressing transcription of p‐GSK3B into the nucleus, and decreases mitoROS accumulation, thereby resulting in the reduction of NDP52‐induced mitophagy. However, the regulatory mechanism of binding PTGS2 to SH3GLB1 is still unclear, the specific role of aloe emodin in mediating the interaction of PTGS2 and SH3GLB1 should be investigated.

By analyzing the GO data in differential proteomics, we propose a hypothesis that the SH3 domain on SH3GLB1 may play an important role in SH3GLB1 binding to PTGS2. To further investigate the role of the SH3 domain in the regulation of mitophagy through PTGS2/SH3GLB1 pathway, we constructed three plasmids, including GFP‐SH3GLB1‐FL, GFP‐SH3GLB1‐△CC, and GFP‐SH3GLB1‐△SH3. After overexpression, we performed co‐IP and found that the interaction of PTGS2 to SH3GLB1 was significantly inhibited in the context of GFP‐△SH3 condition. Also, results showed that GFP‐△SH3 after deletion of SH3GLB1 decreased mitophagy‐related proteins and repressed fusion of damaged mitochondrial and autophagic vesicles. These results suggest that the interaction of PTGS2 and SH3 domain was a key pathophysiological process for aloe emodin alleviating X‐ray‐provoked RIHD development.

Lactylation as a novel posttranslational medication plays a critical regulatory role in cardiovascular diseases.^[^ ^16^, ^46^ ^]^ As first reported by Zhao, lactylation provides a prospective perspective on the pathological mechanisms and drug targets of numerous diseases.^[^ ^15^ ^]^ Lactylation omics showed that P4HB was a potential lactylated protein involved in X‐ray‐induced RIHD. Aloe emodin repressed the binding of Pan‐Lac to P4HB to inhibit lactate dehydrogenase LDHi but not in LDHA deletion. However, the specific interaction site of P4HB for lactylation is unclarified. LC/MS identified lysine residue at position 311 might be a crucial target for P4HB lactylation. Yet, although the K311 site can be recognized by lactate and promote the lactylation of P4HB, the involvement of K311 in SH3GLB1‐mediated mitoROS and NDP52‐induced mitophagy still needs further investigation. Thereby, we constructed two plasmids, myc‐P4HB‐WT and myc‐P4HB‐K311R, and found that P4HB binding to PTGS2 was almost imperceptible after myc‐P4HB‐K311R. Also, myc‐P4HB‐K311R with aloe emodin inhibited interaction between P4HB and K311La, attenuated the co‐localization between mitochondria and lysosomes, and suppressed fusion of damaged mitochondria with autophagic vesicles. However, the regulatory mechanism of P4HB lactylation induced by X‐ray is unknown. A possible regulatory strategy involving in relationship between P4HB lactylation and X‐ray may be the lipid metabolism disorders, especially the abnormal expression of Wnt/β‐catenin pathways.^[^ ^47^, ^48^ ^]^

Endothelial cell‐specific molecule 1 (ESM1) is an acysteine‐rich proteoglycan and is recently associated with inflammatory diseases.^[^ ^49^ ^]^ ESM1 was reported to directly regulate Wnt/β‐catenin signaling, which promoted the development of prostate cancer.^[^ ^50^ ^]^ Our previous study has indicated that binding of ESM1 to angiopoietin‐like 4 (Angptl4) promoted the interaction between Angptl4 and lipoprotein lipase (LPL), leading to the disordered lipid metabolism in ovarian cancer cells.^[^ ^51^ ^]^ These evidence suggests the potential role of ESM1 in regulating P4HB lactylation through disrupting lipid metabolism and accumulation of lactate, may be further involved in the development of RIHD. Future investigations should focus on the specific types in lipid metabolism, such as lipolysis or fatty acid synthesis, that ESM1 can regulate, and also elucidate the mechanisms of ESM1 in the induction of P4HB lactylation by manipulating lipid metabolism.

Our study revealed that aloe emodin alleviates RIHD through the inactivation of NDP52‐induced mitophagy through the PTGS2/SH3GLB1 axis via P4HB lactylation. However, the precise regulatory mechanism of aloe emodin in mitochondrial dysfunction is unknown. We further investigated the metabolic homeostasis disrupted by X‐rays that ultimately led to miROS accumulation and mitophagy activation. The PPI network diagram showed the potential relationship between P4HB and GOT2, suggesting downstream mechanisms induced by GOT2 may be involved in the prevention of aloe emodin in RIHD. GOT2 is a member of the malate‐aspartate shuttle that can catalyze an irreversible transamination, l‐kynurenine to kynurenic acid.^[^ ^33^ ^]^ Kynurenine mainly comes from tryptophan, which depends on IFN‐ γ triggering indoleamine 2,3‐dioxygenase 1 (IDO1) and interleukin‐4 induced 1 (IL4I1), accompanied by indole‐3‐pyruvate production.^[^ ^52^ ^]^ Zhang et al. have reported that serum level of kynurenine was positively correlated with cardiovascular diseases.^[^ ^53^ ^]^ Both data from radiotherapy patients for thoracic tumors and rats treated with aloe emodin showed that kynurenine metabolism was significantly associated with RIHD. However, the regulatory target in kynurenine metabolism for aloe emodin is unknown, and whether this regulatory target was involved in mitoROS and mitophagy needs to be investigated. IP assay showed that aloe emodin inhibited the interaction of GOT2 with Pan‐Lac and P4HB, LC3, NDP52, and ATG16L, respectively, suggesting the provoking role of kynurenine in mitophagy activation. The association between kynurenine and aloe emodin warrants further exploration, as understanding the involvement of P4HB lactylation in imbalanced kynurenine metabolism and mitophagy activation may provide notable insights into the underpinnings of prevention of RIHD by aloe emodin and open new avenues for therapeutic strategies.

In summary, we demonstrated that aloe emodin alleviated RIHD by inhibiting NDP52‐induced mitophagy via repression of SH3GLB1‐mediated mitoROS accumulation. We confirmed that inhibition of p‐GSK3B transcription into the nucleus was the key pathophysiological process of aloe emodin in repressing the interaction of PTGS2 with the SH3 domain of SH3GLB1. Furthermore, we identified P4HB as a novel target for lactylation, and inhibition of K311 site‐linked lactylation was the critical target for alleviation of RIHD by aloe emodin through reducing NDP52‐induced mitophagy. Additionally, aloe emodin stabilized disrupted kynurenine metabolism by inhibiting the accumulation of GOT2 on damaged mitochondria, suggesting that aloe emodin could be a novel potential option for RIHD prevention. Taken together, our results provide a new therapeutic strategy that aloe emodin stabilizes GOT2‐mediated kynurenine metabolism to reduce inflammatory cascade through PTGS2/SH3GLB1/NDP52 axis via regulating K311‐linked P4HB lactylation, which ameliorates RIHD development. However, the precise regulatory mechanism of aloe emodin in stabilizing kynurenine metabolism needs to be further investigated, and translating aloe emodin into clinical use requires further optimization and evaluation.

## 4 Experimental Section

### Antibodies and Reagents

Primary antibodies included anti‐LC3 (Proteintech, 14600‐1‐AP), anti‐p62 (GeneTex, GTX100685), anti‐PINK1 (Proteintech, 23274‐1‐AP), anti‐Parkin (Proteintech, 14060‐1‐AP), anti‐NDP52 (Proteintech, 12229‐1‐AP), anti‐SH3GLB1 (Proteintech, 15422‐1‐AP), anti‐BNP (GeneTex, GTX100538), anti‐cTnT (Proteintech, 26592‐1‐AP), anti‐FIP200 (Proteintech, 17250‐1‐AP), anti‐ULK1 (Proteintech, 29005‐1‐AP), anti‐ATG13 (GeneTex, GTX85183), anti‐Ubiquitin (Proteintech, 10201‐2‐AP), anti‐NDUFA8 (Invitrogen, 43–8800), anti‐SDHA (GeneTex, GTX636335), anti‐NDUFAF1 (Abcam, ab79826), anti‐UQCRH (Proteintech, 12364‐1‐AP), anti‐COX5A (Proteintech, 11448‐1‐AP), anti‐ATP5A (Proteintech, 14676‐1‐AP), anti‐VDAC1 (Proteintech, 55259‐1‐AP), anti‐PTGS2 (Proteintech, 66351‐1‐Ig), anti‐p‐GSK3B (Proteintech, 14850‐1‐AP), anti‐GSK3B (Proteintech, 22104‐1‐AP), anti‐p‐ERK1/2 (Proteintech, 28733‐1‐AP), anti‐ERK1/2 (Proteintech, 11257‐1‐AP), anti‐DDDDK tag (Abcam, ab205606), anti‐HA tag (Abcam, ab236632), anti‐UVRAG (Proteintech, 29190‐1‐AP), anti‐GFP (Abcam, ab6556), anti‐PDI (Proteintech, 66422‐1‐Ig), anti‐Pan‐Lac (PTM BIO, PTM‐1401), anti‐myc (Abcam, ab9106), anti‐ATG5 (Genetex, GTX113309), anti‐ATG16L (Genetex, GTX110619), anti‐Beclin1(Proteintech, 11306‐1‐AP), anti‐VPS34 (Genetex, GTX638372), anti‐STX17 (Genetex, GTX130212), anti‐Tubulin (Proteintech, 11224‐1‐AP), anti‐IDO1 (Proteintech, 13268‐1‐AP), anti‐KMO (Abcam, ab233529), anti‐KYNU (ABclonal, A23962), anti‐HAAO (Genetex, GTX85015), anti‐QPRT (ABclonal, A14349), anti‐NMNAT1 (ABclonal, A6672), and OxPhos rodent WB antibody cocktail (Invitrogen, 45‐8099). HRP‐conjugated goat anti‐rabbit and goat anti‐mouse recombinant secondary antibodies (H+L) were purchased from Proteintech (RGAR001 and RGAM001, respectively). Goat anti‐mouse IgG H+L (FITC) and goat anti‐rabbit IgG H+L (FITC) were purchased from Abcam (ab6785 and ab6717). Goat anti‐mouse IgG H+L (Cy5) and goat anti‐rabbit IgG H+L (Cy3) were purchased from Abcam (ab6563 and ab6939). Aloe emodin, BAI1, and l‐kynurenine were purchased from MedChemExpress (HY‐N0189, HY‐103269, and HY‐1040260). EBSS was purchased from Beyotime (C0213‐500ml). Lactate was purchased from Selleck (E4473). Streptavidin (HRP‐labeled Streptavidin) was purchased from Beyotime (A0303).

### Animal Studies

All animal experiments were conducted with the approval of the Academic and Ethics Committee (AEC) of the University of South China (NO. 2023281). Six‐week‐old male Sprague–Dawley (SD) rats with body weights of 180–200 g were purchased from Hunan SJA Laboratory Animal Company. All SD rats were randomly assigned to three groups of equal size (*n* = 12), including PEG400, 20 Gy X‐ray+PEG400, and 20 Gy X‐ray+Aloe emodin. Aloe emodin was dissolved completely in a 20% PEG400+80% PBS mixture at a dosage of 10 mg kg^−1^ d^−1^, with a final volume of 2000 µL, and poured into Alzet Osmotic Pumps (ALZET 2ML2) that was purchased from YMAIBIO company, which was for pretreatment of 2 weeks. After the pretreatment of aloe emodin, rats were first anesthetized with ketamine (80 mg kg^−1^) and xylazine (10 mg kg^−1^). The rats were laid flat on a supine position and placed on a transparent insulation board on the chest after thoroughly depilating the anterior chest area, and then taken into the working unit of the bio‐irradiator. The radiation probe targeted the precordial area of rats and emitted X‐rays at a dose of 1 Gy per second, each release cooled down for 5 s until the radiation dose accumulated to 20 Gy.^[^ ^11^ ^]^ The rats were raised quietly at SPF for 28 days, and then sacrificed and sampled with the consent and supervision of the AEC. Wild‐type zebrafish lines were purchased from EzeRinka (Nanjing, China), and raised at 28.5 °C in a light/dark cycle according to standard zebrafish husbandry protocols.^[^ ^54^ ^]^ 0.1 or 1 µm aloe emodin was added to the zebrafish culture system for 24 h before treatment with different concentrations of l‐kynurenine. After using 0.04% tricaine methanesulfonate (MS‐222, MCE, HY‐W011777) to anesthetize zebrafish, cardiac morphology of zebrafish was detected by using a tip microscope.

### Echocardiography and Hemodynamic Parameters

All rats were performed by ultrasonic cardiogram at 0 days and 28 days. After anesthetizing with ketamine (80 mg kg^−1^) and xylazine (10 mg kg^−1^) and thoroughly depilating the anterior chest area, echocardiography was performed using an ultra‐phonic machine with a 24‐MHz transducer (MYLAB X8 VET, Yuyan). Left ventricular internal diameter at end‐systole (LVIDs), left ventricular internal diameter at end‐diastole (LVIDd), ejection fraction (EF%), and ratio of LVDd and LVDs (FS%) were monitored and measured by an echocardiographer.

### Collection and Management of Human Serum Samples

Blood samples from different patients were obtained from the Oncology Department, Respiratory Department, Physical Examination Center, and Intensive Care Center of the Second Affiliated Hospital of South China University (Hengyang, China), including normal patients without thoracic tumor, non‐radiotherapy patients for thoracic tumor and radiotherapy patients for thoracic tumor. Approximately 3 mL of venous blood was drawn from the patients. And blood was naturally coagulated at room temperature for 1 h. Then, the blood was added to a tube containing heparin, and immediately centrifuged at 4 °C for 15 min at a speed of 3000 rpm. After centrifugation, the blood was divided into three layers with the middle layer being serum. The serum was stored at −80 °C. The experiment was approved by the AEC of the University of South China (license number: LSK2024040) and conducted in accordance with the Helsinki Declaration.

### Morphological Examination

Before isolating the heart from the thoracic cavity of rats, a mixture solution of PBS and sodium citrate was perfused through the aorta for 15 min to completely remove red blood cells from the cardiomyocyte tissue. Samples for transmission electron microscope (TEM) were immersed in 4% electron microscopy fixative at 4 °C overnight. Samples for morphological staining were immersed in 4% PFA at 4 °C overnight and embedded in OCT compound to prepare frozen sections (10µm). For paraffin sections (5µm), samples were dehydrated using degraded ethanol and xylene.^[^ ^55^ ^]^ For Masson staining and Siruis red staining, paraffin sections were dewaxed using xylene and degraded ethanol, and rinsed in ddH_2_O, and then the prepared sections were stained and incubated according to the manufacturer's protocol and standard procedures. For immunohistochemistry (IHC), prepared sections were detected by IHC staining kit purchased from Beyotime (P0628). Sections were treated with sodium citrate buffer at 95 °C for 10 min, then interacted with 3% hydrogen peroxide for 10 min. Subsequently, primary and secondary antibodies were performed to incubate the sections. DAB staining kit was used to detect the positive signals (Beyotime, P0203). For immunofluorescence (IF), sections were incubated with different primary antibodies from anti‐rabbit or anti‐mouse overnight. Then, the correspondent host/isotype of second antibodies, FITC, Cy3, or Cy5, were conjugated with the primary antibodies for 1 h at 37 °C. After washing with PBS three times, antifade mounting medium with DAPI was used to marker nuclei, and a fluorescence microscope (ZEISS) for immunofluorescence or a laser confocal microscope (Nikon) was used to capture fluorescent images with the appropriate fluorescence excitation wavelength, and the results were analyzed with NIS‐Viewer, Gen‐Viewer and GraphPad Prism 9.

### Cell Culture and Western Blotting

H9C2 (2‐1) cells and HEK293T cells were purchased from Procell (CL‐0089, CL‐0005) and cultured in Dulbecco's modified Eagle's medium (Gibco, 11965092) with 10% fetal bovine serum (Gibco, 10099141C). All siRNA interference reagents were designed and produced by GenePharma (Suzhou, China). The sequences were as follows: Rattus norvegicus SH3GLB1 (5′‐UAAACAAAUUCUUCUAUCCUG‐3′ and 3′‐GUAUUUGUUAAGAAGAUAGG‐5′), Rattus norvegicus PTGS2 (5′‐AUAUUGGUCAAAUCCUAUGCU‐3′ and 3′‐AAUAUAACCAGUUUAGGAUAC‐5′) and Rattus norvegicus LDHA (5′‐UGAGAAGUGCGUGGUCUCCAU‐3′ and 3′‐CUACUCUUCACGCACCAGAGG‐5′). VectorBuilder (Guangzhou, China) designed and produced all overexpression plasmids, tag plasmids, truncated plasmids, and point mutation plasmid. For transfection, 5 µL siRNA or 2 µg plasmid was added into six‐well plates using DNA/RNA transfer reagent (Invigentech, IV1216050). Twenty‐four hours post‐transfection, experimental treatment commenced as the medium was refreshed. H9C2 cells were treated with aloe emodin at a final concentration of 2 µm in a six‐well plate. The cells were then subjected to bi‐irradiation at doses ranging from 1 Gy to a maximum of 4 Gy, with treatment durations not exceeding 24 h, in accordance with a previously established cell model.^[^ ^11^ ^]^ Protein samples were lysed with a mix of RIPA lysis buffer and cock‐tail, followed by centrifugation at 12 000 × *g* at 4 °C. The protein supernatant was quantified using BCA assay and then denatured with 5X loading buffer at a volume ratio of 1:4. The denatured protein samples were stored at −80 °C. 10% precast page gels with either 10 or 15 wells were obtained from Absin (abs9301, abs9308) for use with proteins of various molecular weights. Following electrophoresis at 150 V for 35 minutes, the proteins were transferred to PVDF membranes with 0.45‐µm pore size. The uncropped PVDF membranes were blocked in 5% milk at room temperature for 1 h and subsequently incubated with primary antibodies overnight at 4 °C. After washing with TBST three times, the PVDF membranes were probed with secondary antibodies and visualized using imaging systems.

### Molecular Docking Analysis

To analyze the binding mode and binding affinities between aloe emodin and P4HB, molecular docking was performed using software (Autodock Vina 1.2.2, http://autodock.scripps.edu). The molecular formula of aloe emodin (C_15_H_10_O_5_, PubChem CID: 10 207) was obtained from PubChem Compound (https://pubchem.ncbi.nlm.nih.gov/), and the 3D coordinates of P4HB (PDB ID: 8EOJ; resolution, 2.5 Å) was obtained from PDB website (http://www.rcsb.org/pdb/home/home.do). All water molecules were removed from aloe emodin and P4HB files before docking. Polar hydrogen atoms were then added. It was centered to cover the domains of each protein and to allow for free molecular movement, with 0.05 nm for grid point distance and 30 Å × 30 Å × 30 Å of grid box.

### Synthetic Route of AE‐Biotin

Biotin (244 mg, 1 mmol) was dissolved in 10 mL of anhydrous DMF, and 1‐(3‐Dimethylaminopropyl)‐3‐ethylcarbodiimide hydrochloride (240 mg, 1.2 mmol) and 4‐Dimethylaminopyridine (12 mg, 0.1 mmol) were added. The reaction mixture was allowed to react for 10 min, after which aloe emodin (300 mg, 1.1 mmol) was added. After overnight reaction at room temperature, 50 mL of dichloromethane was added, and the mixture was washed three times with 80 mL of saturated saline water. The organic phase was collected and dried with anhydrous Na₂SO₄. Purification was performed on a 200–300 mesh silica gel column, yielding a yellow solid (200 mg, 40% yield). The product was characterized by ^1^H NMR (400 MHz, DMSO‐*d*_6_) δ 11.90 (s, 2H), 7.81 (t, *J* = 7.9 Hz, 1H), 7.71 (d, *J* = 7.4 Hz, 1H), 7.66 (s, 1H), 7.38 (d, *J* = 9.0 Hz, 1H), 7.33 (s, 1H), 6.37 (d, *J* = 30.4 Hz, 2H), 5.22 (s, 2H), 4.33 – 4.27 (m, 1H), 4.17 – 4.11 (m, 1H), 3.14 – 3.06 (m, 1H), 2.81 (dd, *J* = 12.4, 5.1 Hz, 1H), 2.57 (d, *J* = 12.4 Hz, 1H), 2.45 (t, *J* = 7.4 Hz, 2H), 1.65 – 1.58 (m, 2H), 1.36 (d, *J* = 17.0 Hz, 2H), 1.24 (d, *J* = 9.1 Hz, 2H). ^13^C NMR (100 MHz, DMSO‐*d*_6_) δ 192.04, 181.69, 173.09, 163.17, 161.83, 147.07, 137.92, 133.95, 133.74, 124.96, 122.59, 119.87, 118.20, 116.41, 115.81, 64.62, 61.51, 59.68, 55.80, 33.68, 28.46, 24.99.

### Co‐Immunoprecipitation

Cells were washed twice with pre‐cooled PBS, and dried the PBS for the last time. Then cells were added with 0.5 mL pre‐cooled RIPA buffer per 5 × 10^6^ cells. A pre‐cooled cell scraper was used to scrape the cells off the culture dish or bottle, the suspension was transferred to a 1.5 mL EP tube, and slowly shaked at 4 °C for 15 min. Centrifuge at 14 000 × *g* for 15 min at 4 °C, and immediately transfer the supernatant to a new EP tube. Protein A agarose was washed twice with PBS and then prepared with PBS at a concentration of 50%. One hundred microliters of Protein A agarose beads (50%) per 1 mL protein lysates were added into supernatant and shaken on a horizontal shaker at 4 °C for 10 min to remove non‐specific impurities and reduce background. The sample was centrifuged at 4 °C and 14 000 × *g* for 15 min. The supernatant was transferred to a new EP tube, and Protein A beads were removed. A protein standard curve was created using the Bradford method to measure protein concentration. The total protein was diluted to ≈1 µg µL^−1^ using PBS to reduce the concentration of the descaling agent in the lysis solution. Antibody (2 µL) was added to 100 µL of total lysates, and the antigen‐antibody mixture was gently shaken overnight at 4 °C. Protein A agarose beads (100 µL) were added to capture antigen‐antibody complexes, and the mixture was again gently shaken overnight at 4 °C. The mixture was centrifuged at 14 000 rpm for 5 s, and the agarose bead antigen‐antibody complex was collected. The supernatant was removed, and the complex was washed three times with pre‐cooled RIPA buffer, followed by three washes with PBS. The agarose bead antigen‐antibody complex was resuspended in 60 µL of 2× loading buffer and gently mixed. The sample was incubated in a metal bath for 5 min to dissociate antigens, antibodies, and beads, then centrifuged, and the supernatant was stored at −20 °C for further electrophoresis.

### Immunocolloidal Gold Transmission Electron Microscopy

Immunocolloidal gold transmission electron microscopy is an immunolabeling method applied at the electron microscopy level, which can be divided into pre‐embedding staining and post‐embedding staining. Due to the poor penetrability of pre‐embedding staining to the cell membrane, it is generally only used for antigen labeling on the cell surface. If it needs to penetrate the cell membrane, TritonX‐100 needs to be added, but it will aggravate the damage to the cell's ultrastructure. Therefore, post‐embedding staining gains much more applications. Ultrathin sections with a tissue thickness of 70 nm were loaded onto nickel mesh with a mesh size of 200–300. 1% H_2_O_2_ solution was dropped onto a wax plate and incubated for 30 min to remove osmic acid and enhance resin penetration. The wax plate was washed with dd H_2_O three times, each time for 10 min. For the first and second times, the wax plate should float on the liquid droplet. For the third time, the nickel mesh surface was rinsed with a syringe containing dd H_2_O. The water flow was under appropriate pressure. Filter paper was used to absorb the water at the edge of the mesh. Normal goat serum (1:50) was dripped at room temperature for 30 min, and the non‐specific binding site was occupied with free aldehyde groups in the saturated fixative. The filter paper was dried, the primary antibody was dropped onto a wax plate, pre‐incubated at room temperature for 1 h, and then at 4 °C for 24 h. Washed with PBS three times, each time for 3 min. PBS containing 1% BSA with a pH of 8.2 was prepared and floated on wax for 5 min. Subsequently, the wax plate was immersed in 1% colloidal gold‐labeled antibody solution for incubation at room temperature for 1 h. Washed with dd H_2_O three times, each time for 3 min. Stained with 5% uranium acetate for 5 min, then the wax was washed with dd H_2_O three times. Stained with lead acetate for 5 min, and the wax was washed with dd H_2_O three times, eventually an electron microscope was used for observation.

### Labexluminex

The technical principle was to use two red‐classified fluorescent dyes with different ratios to dye polystyrene microspheres with a diameter of 5.6 microns into different fluorescent colors, thereby obtaining over 100 fluorescent encoded microspheres. Antibodies or probes targeting different analytes were bound to specific coding microspheres through covalent crosslinking, with each coding microsphere corresponding to a corresponding detection item. First, fluorescent coding microspheres targeting different analytes were mixed, then the analyte or amplified fragment was added to form a complex that then binds to labeled fluorescein. Under the driving force of the flowing sheath, the microspheres passed through red and green lasers in a single row. The red laser was used to determine the fluorescence coding of the microspheres, while the green laser was used to measure the fluorescence intensity of the reporting molecules on the microspheres to achieve the goal of rapid and accurate quantitative detection. Before the experiment, the reagents were moved to room temperature and equilibrated for 30 min. According to the kit protocol, serum samples were added with beads, standard, and quality control in order of 25–50 µL and incubated at room temperature of 800 rpm overnight at 4 ° C, then serum samples were added to 100 µL cleaning solution for washing three times. Subsequently, 25–50 µL antibodies were added to serum samples at room temperature of 800 rpm, incubated for 1 h, and added to 100 µL cleaning solution for washing three times. Fifty microliters of PE streptavidin incubated serum samples at room temperature of 800 rpm for 30 min and 100 µL cleaning solution for washing three times. One hundred microliters of moisturizing solution incubated serum samples at room temperature of 800 rpm for 0.5–2 min. Univ Bio company (Shanghai, China) provided technical support, machine testing, and data acquisition.

### Oxygen Consumption Rate (OCR)

H9C2 cells were inoculated at a predetermined density onto Seahorse XF cell culture plates using appropriate culture media, and a100 µL were added to l‐carnitine. 18.5 mL of culture medium (without glucose, sodium pyruvate, glutamine, or GlutaMAX) was added to a 50 mL sterile conical tube, and supplemented with 0.5 mmol L^−1^ of glucose, 1 mmol L^−1^ of glutamine or GlutaMAX, and 1% FBS. Twenty microliters of prepared l‐carnitine solution was added. One hundred microliters were added to each hole substrate‐restricted growth medium in a 24‐well plate. The Seahorse XF96 was preheated overnight to stabilize the temperature. The sensor probe plate was placed in sterile distilled water and hydrated overnight in a CO_2_‐free incubator at 37 °C. An analysis template file was created in the WAVE desktop software using an advanced experimental template for palmitic acid oxidation pressure testing. Water was removed and an appropriate volume of XF calibration solution was added to each well, placed in a CO_2_‐free incubator, and incubated at 37 °C for 60 min. 2 mmol L^−1^ XF glucose and 0.5 mmol L^−1^ l‐carnitine were added to 75 mL Seahorse XF DMEM and preheated at 37 °C. Substrate restriction growth medium was removed from the cell culture microplate. Washed once with preheated detection solution. Before analysis, the cell plate containing substrate restriction detection solution was placed in a CO_2_‐free incubator and incubated at 37 °C for 45–60 min. Before starting XF analysis, the detection solution was removed from the cell plate again and 415 µL pre‐heated fresh detection solution was added to each well. Finally, 85 µL 1× palmitic acid‐BSA was added to the corresponding wells of the 24‐well plate. Oligomycin (blue cap), FCCP (yellow cap), and rotenone/antifungal A (red cap) were added. The small bottle was gently tapped to ensure that the powder is at the bottom of the bottle, then the cap opener provided by the reagent kit was used to open the bottle cap. The contents of each small bottle were resuspended in an appropriate volume of prepared detection solution. Vortexed for 1 min to ensure sufficient resuspension of the compound. A working solution was prepared using compound stock solution for loading to the dosing hole of the sensor probe plate. The resuspended drug was diluted into the desired concentration of drug‐working solution using the detection solution, with ≈2 mL of each drug‐working solution. Two milliliters of working solution was prepared for each compound using palmitic acid oxidation pressure test solution according to the volume shown in the instruction manual, with 1.5 µm oligomycin and 0.5 µm rotenone/antimycin A. Agilent company (Beijing, China) provided technical support, machine testing, and data acquisition.

### Differential Protein Omics

Cells were sonicated three times on ice using a high‐intensity ultrasonic processor (Scientz) in lysis buffer (8 m urea, 1% protease inhibitor cocktail). (Note: For PTM experiments, inhibitors were also added to the lysis buffer, e.g., 3 µm TSA and 50 mm NAM for acetylation, 1% phosphatase inhibitor for phosphorylation). The remaining debris was removed by centrifugation at 12 000 × *g* at 4 °C for 10 min. Finally, the supernatant was collected and the protein concentration was determined with BCA kit according to the manufacturer's instructions. For digestion, the protein solution was reduced with 5 mm dithiothreitol for 30 min at 56 °C and alkylated with 11 mm iodoacetamide for 15 min at room temperature in darkness. The protein sample was then diluted by adding 100 mm TEAB to urea concentration less than 2 m. Finally, trypsin was added at 1:50 trypsin‐to‐protein mass ratio for the first digestion overnight and 1:100 trypsin‐to‐protein mass ratio for a second 4 h‐digestion. Finally, the peptides were desalted by C18 SPE column. PTM BIO company (Hangzhou, China) provided technical support, machine testing, and data acquisition.

### Lactylation Modification Omics of 4D Label‐Free

The cells were ground with liquid nitrogen into cell powder and then transferred to a 5‐mL centrifuge tube. After that, four volumes of lysis buffer (8 m urea, 1% protease inhibitor cocktail) were added to the cell powder, followed by sonication for 3 min on ice using a high‐intensity ultrasonic processor (Scientz). The remaining debris was removed by centrifugation at 12 000 × *g* at 4 °C for 10 min. Finally, the supernatant was collected and the protein concentration was determined with BCA kit. The cells were slowly added to the final concentration of 20% (m/v) TCA to precipitate protein, then vortexed to mix and incubated for 2 h at 4 °C. The precipitate was collected by centrifugation at 4500 × *g* for 5 min at 4 °C. The precipitated protein was washed with precooled acetone three times and dried for 1 min. The protein sample was then redissolved in 200 mm TEAB and ultrasonically dispersed. Trypsin was added at 1:50 trypsin‐to‐protein mass ratio for the first digestion overnight. The sample was reduced with 5 mm dithiothreitol for 30 min at 56 °C and alkylated with 11 mm iodoacetamide for 15 min at room temperature in darkness. Finally, the peptides were desalted by Strata X SPE column. To enrich modified peptides, tryptic peptides dissolved in NETN buffer (100 mm NaCl, 1 mm EDTA, 50 mm Tris‐HCl, 0.5% NP‐40, pH 8.0) were incubated with pre‐washed antibody beads (Lot number xxx, PTM Bio) at 4 °C overnight with gentle shaking. Then the beads were washed four times with NETN buffer and twice with H_2_O. The bound peptides were eluted from the beads with 0.1% trifluoroacetic acid. Finally, the eluted fractions were combined and vacuum‐dried. For LC–MS/MS analysis, the resulting peptides were desalted with C18 ZipTips (Millipore) according to the manufacturers’ instructions. PTM BIO company (Hangzhou, China) provided technical support, machine testing, and data acquisition.

### LC–MS/MS Analysis

The tryptic peptides were dissolved in solvent A, directly loaded onto a homemade reversed‐phase analytical column (25 cm length, 100 µm i.d.). The mobile phase consisted of solvent A (0.1% formic acid, 2% acetonitrile/in water) and solvent B (0.1% formic acid in acetonitrile). Peptides were separated with following gradient: 0–40 min, 7–24%B; 40–52 min, 24–32%B; 52–56 min, 32–80%B; 56–60 min, 80%B, and all at a constant flow rate of 450 nL min^−1^ on a NanoElute UHPLC system (Bruker Daltonics). The peptides were subjected to a capillary source followed by the timsTOF Pro 2 mass spectrometry. The electrospray voltage applied was 1.5 kV. Precursors and fragments were analyzed at the TOF detector, with an MS/MS scan range from 100 to 1700. The timsTOF Pro was operated in parallel accumulation serial fragmentation (PASEF) mode. Precursors with charge states 0–5 were selected for fragmentation, and 10PASEF‐MS/MS scans were acquired per cycle. The dynamic exclusion was set to 24 s. The resulting MS/MS data were processed using MaxQuant search engine (v.1.6.15.0). Tandem mass spectra were searched against Rattus_norvegicus_10116_PR_20230103.fasta (47945 entries) concatenated with a reverse decoy and contaminants database. Trypsin/P was specified as cleavage enzyme allowing up to two missing cleavages. Minimum peptide length was set as 7 and maximum number of modifications per peptide was set as 5. The mass tolerance for precursor ions was set as 20 ppm in the first search and 20 ppm in the main search, and the mass tolerance for fragment ions was set as 20 ppm. Carbamidomethyl on Cys was specified as a fixed modification. Acetylation on protein N‐terminal, oxidation on Met, and lactylation were specified as variable modifications. False discovery rate (FDR) of protein, peptide, and PSM was adjusted to <1%. PTM BIO company (Hangzhou, China) provided technical support, machine testing, and data acquisition.

### GO Annotation

Gene Ontology analysis (GO analysis) is a bioinformatics analysis method that provides statistical information by organically linking information about genes and gene products (e.g., proteins). In proteomics projects, GO is mainly used for the following purposes: 1) as a database for various information of proteins and genes; 2) to provide various information of proteins and genes, and to classify them according to the information; 3) as a tool to provide the most comprehensive information annotation and classification service for all proteins in the project. GO analysis mainly includes three aspects: 1) Cellular component refers to the specific components of a cell that are considered as constituent parts of larger cellular structures within the GO system. For example, anatomically certain cellular structures (rough endoplasmic reticulum, nucleus, etc.), or a series of gene products such as the foundational structures of some complex components (ribosomes, protein dimers, etc.); 2) Molecular function refers primarily to the chemical activities of molecules that can be manifested at the molecular level, including catalytic activities or binding activities; 3) Biological process is the term used to describe the ordered and specific execution of a particular function within an organism by a group of molecules. The process of GO annotation involved using the eggnog‐mapper software to extract GO IDs from the identified proteins based on the EggNOG database (v5.0.2, http://eggnog5.embl.de/#/app/home), and then performing functional classification annotation analysis on the proteins according to cellular components, molecular functions, and biological processes. PTM BIO company (Hangzhou, China) provided bioinformatics methods and analysis.

### KEGG Pathway Annotation

The Kyoto Encyclopedia of Genes and Genomes (KEGG) integrates currently known protein‐protein interaction network information, such as pathways and related complexes (Pathway database), genes and gene products (Gene database), biological complexes and related reactions (Compound and Reaction databases), and other information. The KEGG pathways mainly include metabolism, genetic information processing, environmental information processing, cellular processes, human diseases and drug development. Protein pathways were annotated based on the KEGG pathway database, including KAAS (2015, http://www.genome.jp/kegg/kaas/), KEGG mapper (v5.0, http://www.kegg.jp/kegg/mapper.html), KOBAS (v3.0, http://kobas.cbi.pku.edu.cn/) and KEGG mapper (v5.0, http://www.kegg.jp/kegg/mapper.html), and proteins were identified through BLAST comparison (blastp, evalue ≤ 1e‐4), for each sequence, the annotation was based on the top‐scoring comparison result. PTM BIO company (Hangzhou, China) provided bioinformatics methods and analysis.

### Domain Annotation

The structural domain of a protein is a specific protein region in a protein that is conserved in sequence and can generally perform a function independently, and is a structural component of molecular function, generally consisting of 25–500 amino acids. These areas are relatively spatially compact, structurally stable, and capable of being independently folded into functional structures. A protein may have multiple domains, and a domain may also exist in multiple proteins. In the project data, protein structural domain annotation was performed on the identified proteins based on the Pfam database (Pfam‐A.hmm‐33.1, https://www.ebi.ac.uk/interpro/entry/pfam/#table). PTM BIO company (Hangzhou, China) provided bioinformatics methods and analysis.

### Protein–Protein Interaction Network

All differentially expressed protein database accession or sequence were searched against the STRING database (v11.5, https://cn.string‐db.org/) for protein–protein interactions. Only interactions between the proteins belonging to the searched data set were selected, thereby excluding external candidates. STRING defines a metric called “confidence score” to define interaction confidence; all interactions that had a confidence score > 0.7 (high confidence) were fetched. Interaction network form STRING was visualized in R package “visNetwork”. PTM BIO company (Hangzhou, China) provided bioinformatics methods and analysis.

### Statistics

All data were present as the mean ± standard error of the mean (SEM). Quantification data for comparisons between groups were evaluated by unpaired two‐tailed Student's *t*‐test or one‐way ANOVA by GraphPad Prism 10. The criterion for statistical significance was *p* < 0.05.

## Conflict of Interest

The authors declare no conflict of interest.

## Author Contributions

A.B.G. and Y.K.L. conceptualized and supervised this study. F.OY., A.B.G., Y.K.L., Y.L.L., K.H., Q.L., and X.Z. performed the experiments. F.OY., A.B.G., Y.K.L., H.M.W., X.Y.L., X.L.T., G.Y.X., J.F.Z., S.M.C., J.R.F., and L.L.Z. analyzed and interpreted the data. F.OY. wrote the draft. A.B.G., Y.K.L., Y.L.L., and H.M.W. polished the paper. F.OY., Y.K.L., and A.B.G. summarized and drew the mechanistic diagram. J.F.Z., G.F.Z., and Q.L. collected and screened human clinical samples. H.Z. and X.Z. performed radiological manipulation and imaging analysis. X.L.T., S.M.C., and J.R.F. performed the bio‐information analysis and software manipulation. Y.L.L, H.M.W., X.Y.L., X.L.T., and G.Y.X raised rats and zebrafish. J.F.Z., G.F.Z., H.Z., J.R.F., X.Z., and L.L.Z. provided partial funds for the experiment. A.B.G. and Y.K.L. provided all the necessary funds for the experiment.

## Supporting information



Supporting Information

## Data Availability

The data that support the findings of this study are available from the corresponding author upon reasonable request.
