# Correction to “[Aloe Emodin Alleviates Radiation‐Induced Heart Disease via Blocking P4HB Lactylation and Mitigating Kynurenine Metabolic Disruption]”

**DOI:** 10.1002/advs.202501837

**Published:** 2025-02-25

Ouyang F, Li Y, Wang H, Liu X, Tan X, Xie G, Zeng J, Zeng G, Luo Q, Zhou H, Chen S, Hou K, Fang J, Zhang X, Zhou L, Li Y, Gao A. Aloe Emodin Alleviates Radiation‐Induced Heart Disease via Blocking P4HB Lactylation and Mitigating Kynurenine Metabolic Disruption. *Adv. Sci*. **2024**, 11(47), e2406026.

https://doi.org/10.1002/advs.202406026

Following the publication of this article, we conducted a comprehensive review and archiving of the raw data, during which we regrettably identified errors in Figure 6, Figures S14 and S25 (Supporting Information). These errors were unintentionally introduced while organizing the figures. It is likely due to the duplicate copying of data in the experimental equipment caused by the relocation of the laboratory from the affiliated hospital to the University of South China. The corrected versions of these figures are provided below. We would like to emphasize that these errors do not affect the results or conclusions of the article. We sincerely apologize for any inconvenience this may have caused.

In Figure 6C, the WB band of NDP52 from lysate appeared incorrectly. We request to correct Figure 6C as follows:


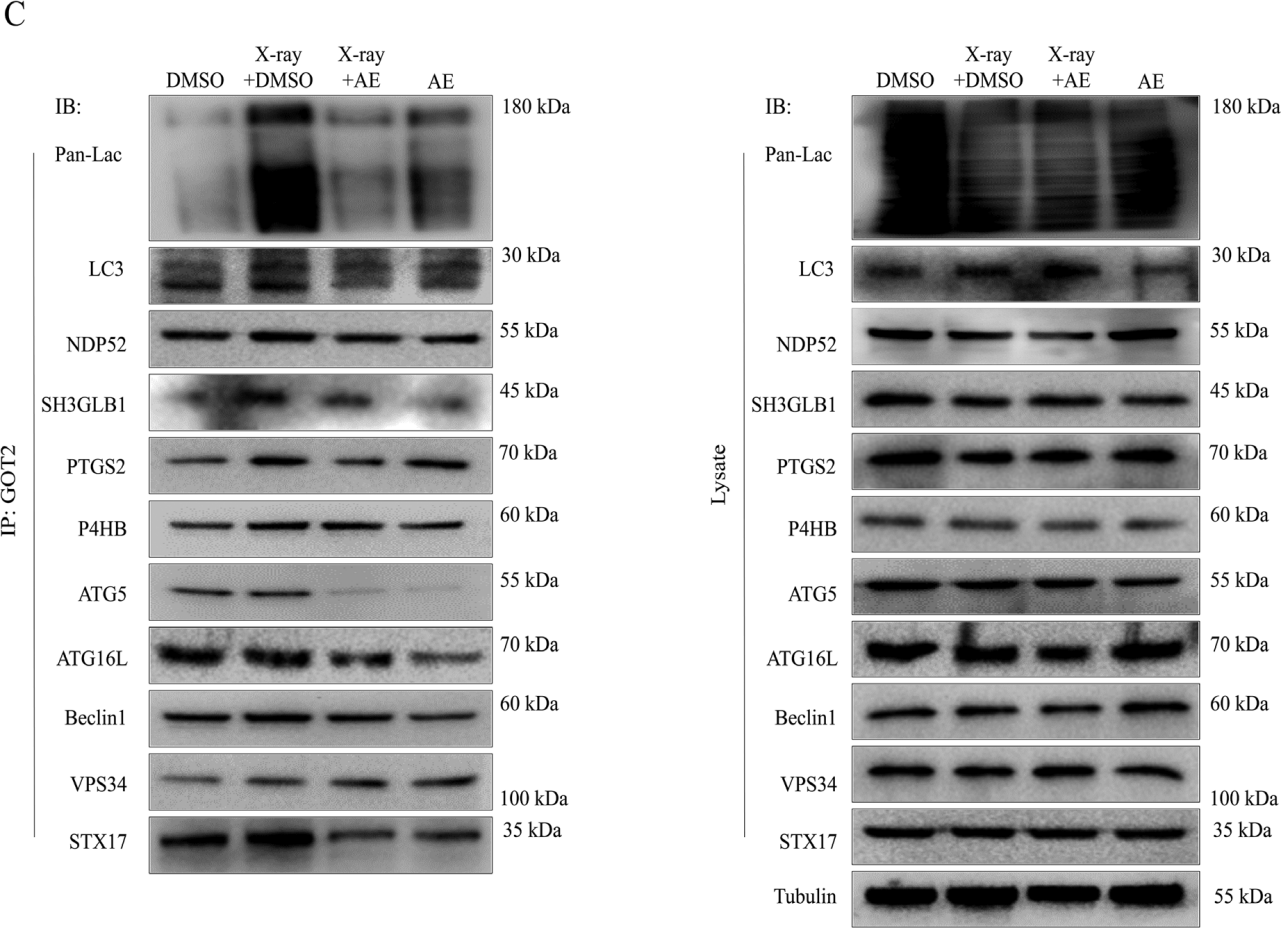


In Figure S14 (Supporting Information), the WB band of Parkin appeared incorrectly. We request to correct Figure S14 (Supporting Information) as follows:


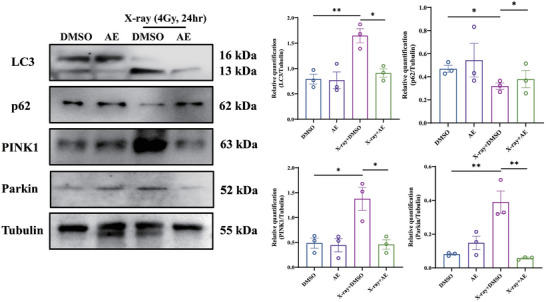


In Figure S25 (Supporting Information), the fluorescence image of mitoROS from the DMSO+siSH3GLB1+GFP‐FL group was incorrectly assembled. We request to correct Figure S25 (Supporting Information) as follows:


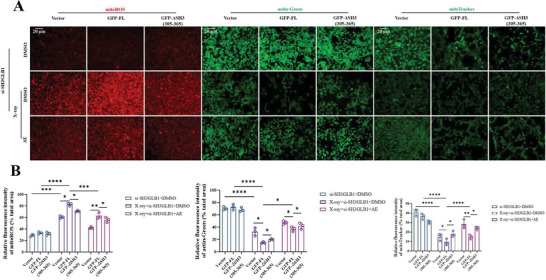


We apologize for this error.

