# Supplementary material for: Aloe Emodin Alleviates Radiation‐Induced Heart Disease via Blocking P4HB Lactylation and Mitigating Kynurenine Metabolic Disruption
Source: Adv Sci (Weinh). 2024 Nov 4;11(47):2406026. doi: 10.1002/advs.202406026 (PMC11653682; doi:10.1002/advs.202406026)
Supplement: Supplementary file 1 — Supporting Information [file ADVS-11-2406026-s001.docx]

**Supplementary Figures**


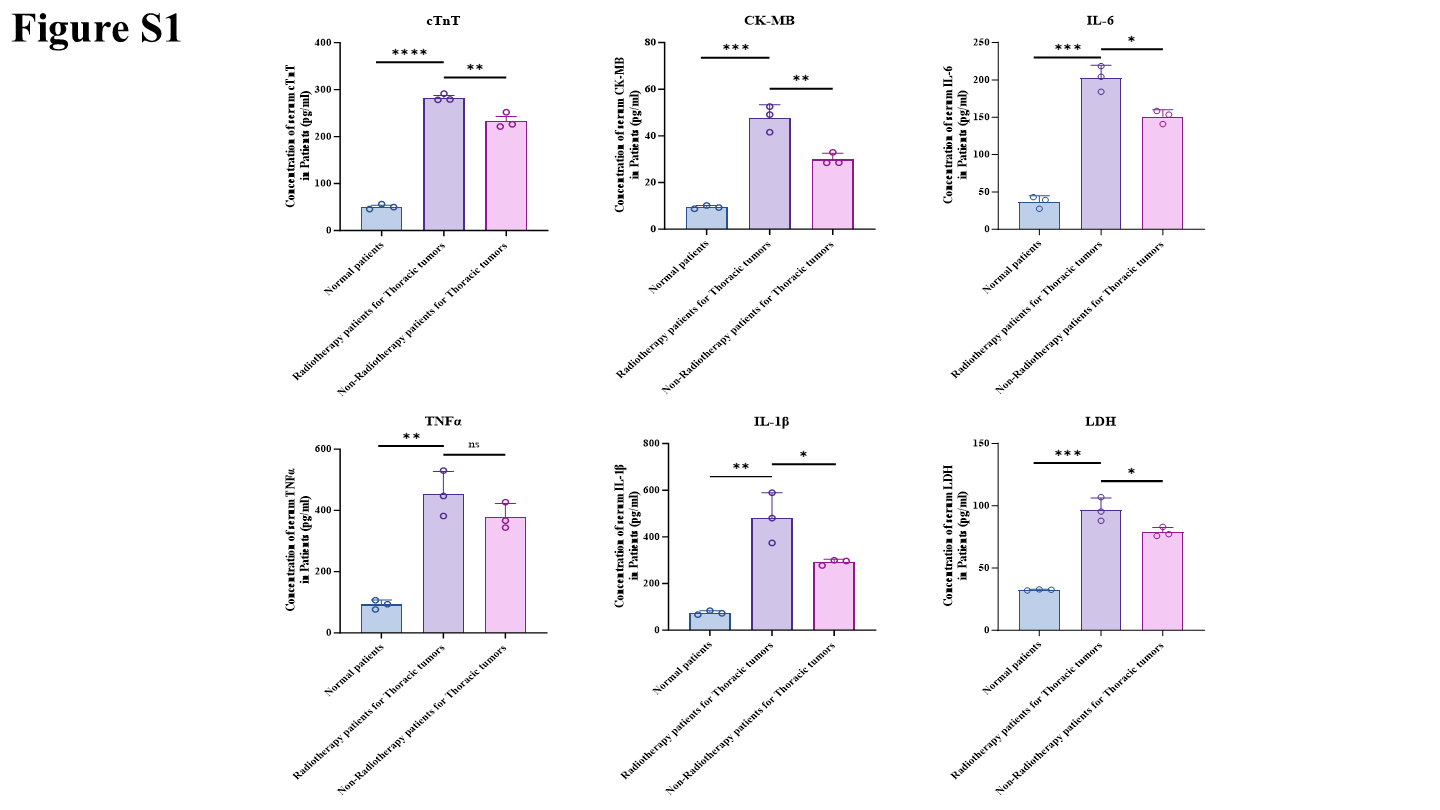


**Figure S1.** Levels of cardiac injury markers. Elisa assay of cTnT, LDH, CK-MB, IL-1β, IL-6 and TNFα in no-treated patients, radiotherapy patients for thoracic tumors or non-radiotherapy patients for thoracic tumors. Quantification data are shown as mean ± SEM. All data were obtained from 3 independent experiments. *P* values were calculated by unpaired two-tail Student’s *t*-test or one-way ANOVA. **P* < 0.05; ***P* < 0.01; ****P* < 0.001; *****P* < 0.0001; ns: not significant.


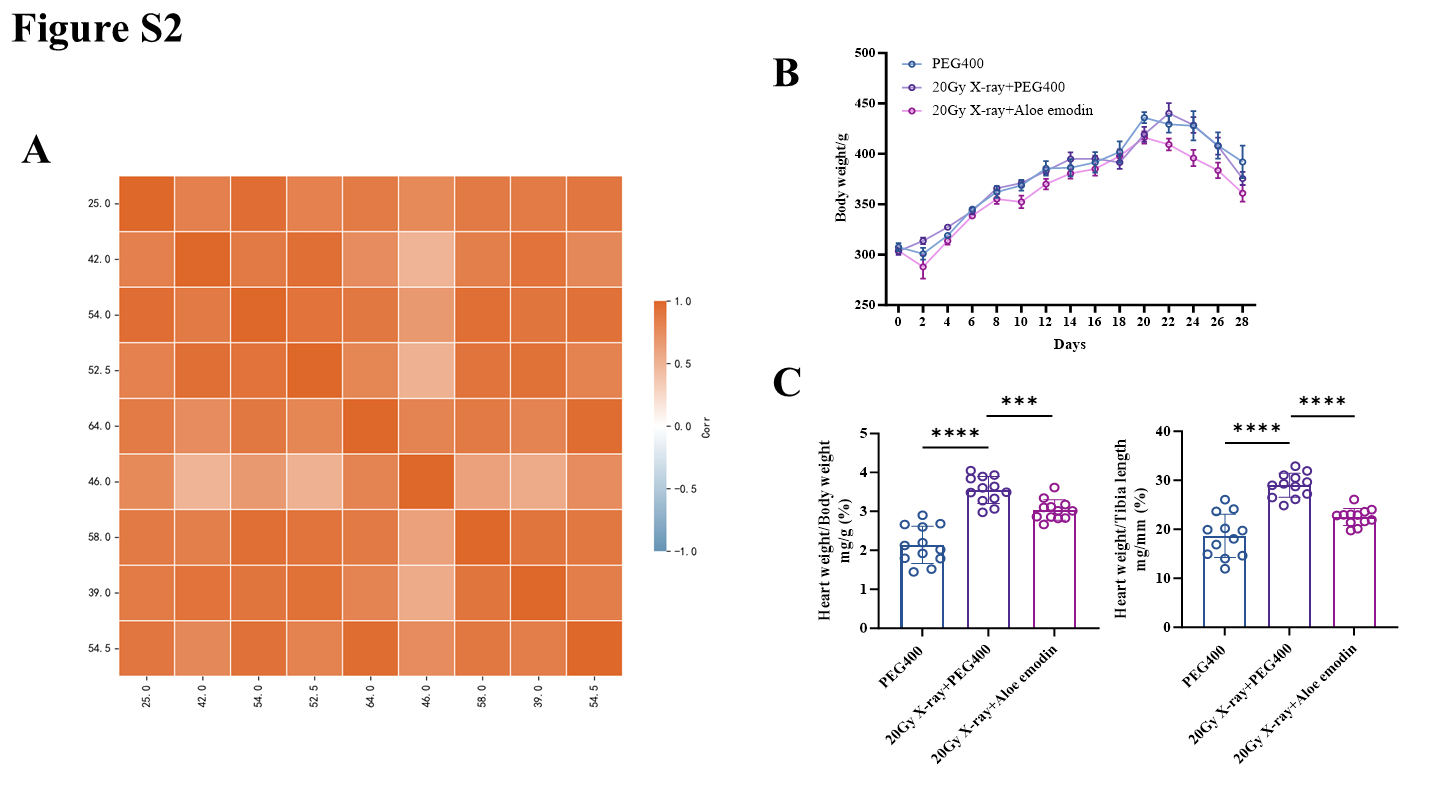


**Figure S2.** A) Correlation analysis among sample in human serum samples. B, C) Alteration of body weights, ratio of heart weight to body weight, and ratio of heart weight to tibia length in rats after X-ray or aloe emodin treatment. Quantification data are shown as mean ± SEM. All data were obtained from 12 independent experiments. *P* values were calculated by unpaired two-tail Student’s *t*-test or one-way ANOVA. ****P* < 0.001; *****P* < 0.0001.


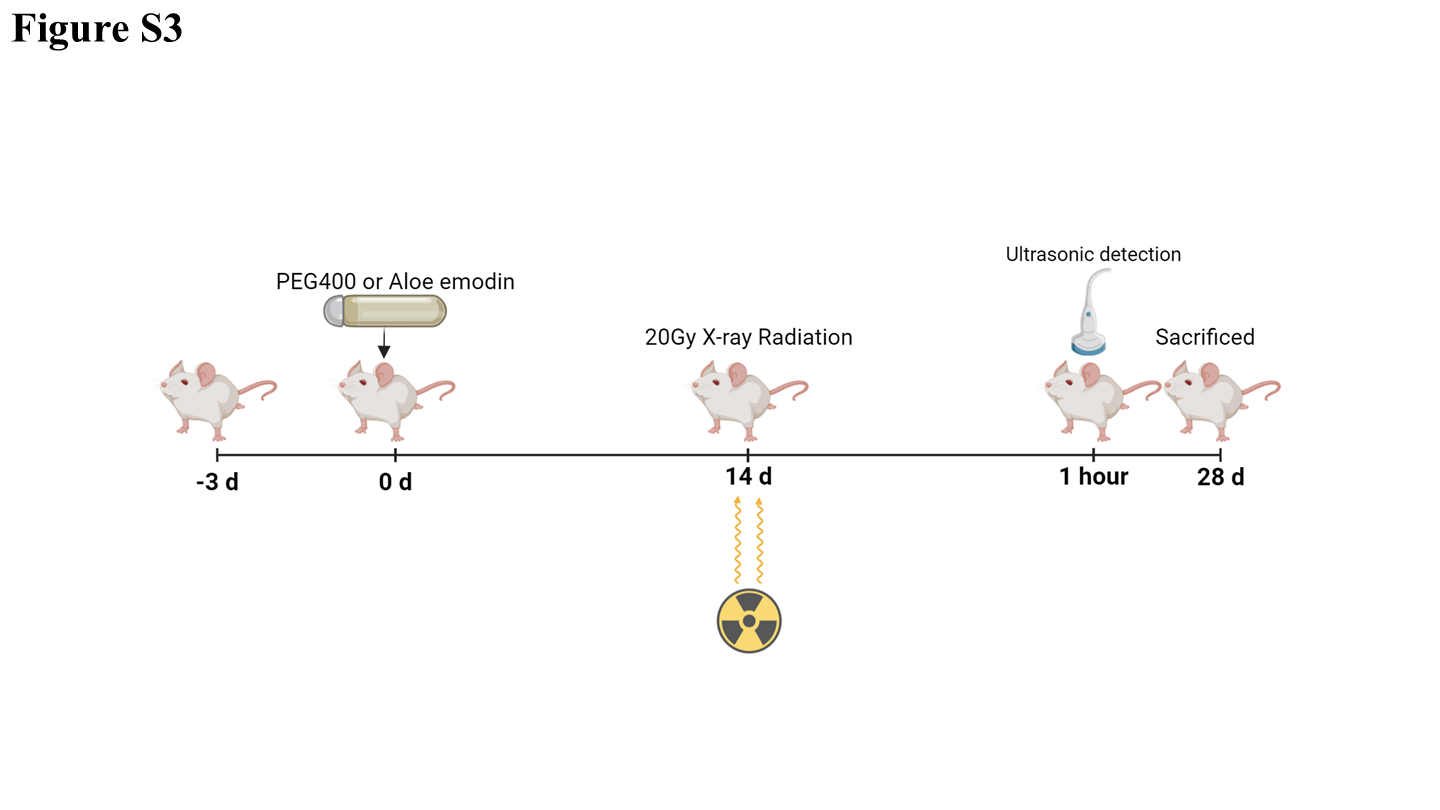


**Figure S3.** Schematic workflow of rat with Alzet Ostomic pumps filled with PEG400 or aloe emodin. After 3 days of adaptive feeding of rats, ostomic pumps were embedded in the abdominal cavity under anesthesia. At 14 days, ostomic pumps were removed from the rat body and subjected to radiation of 20Gy X-ray in the precordial area under anesthesia. One hour before execution, rats were performed echocardiography to obtain imaging data.


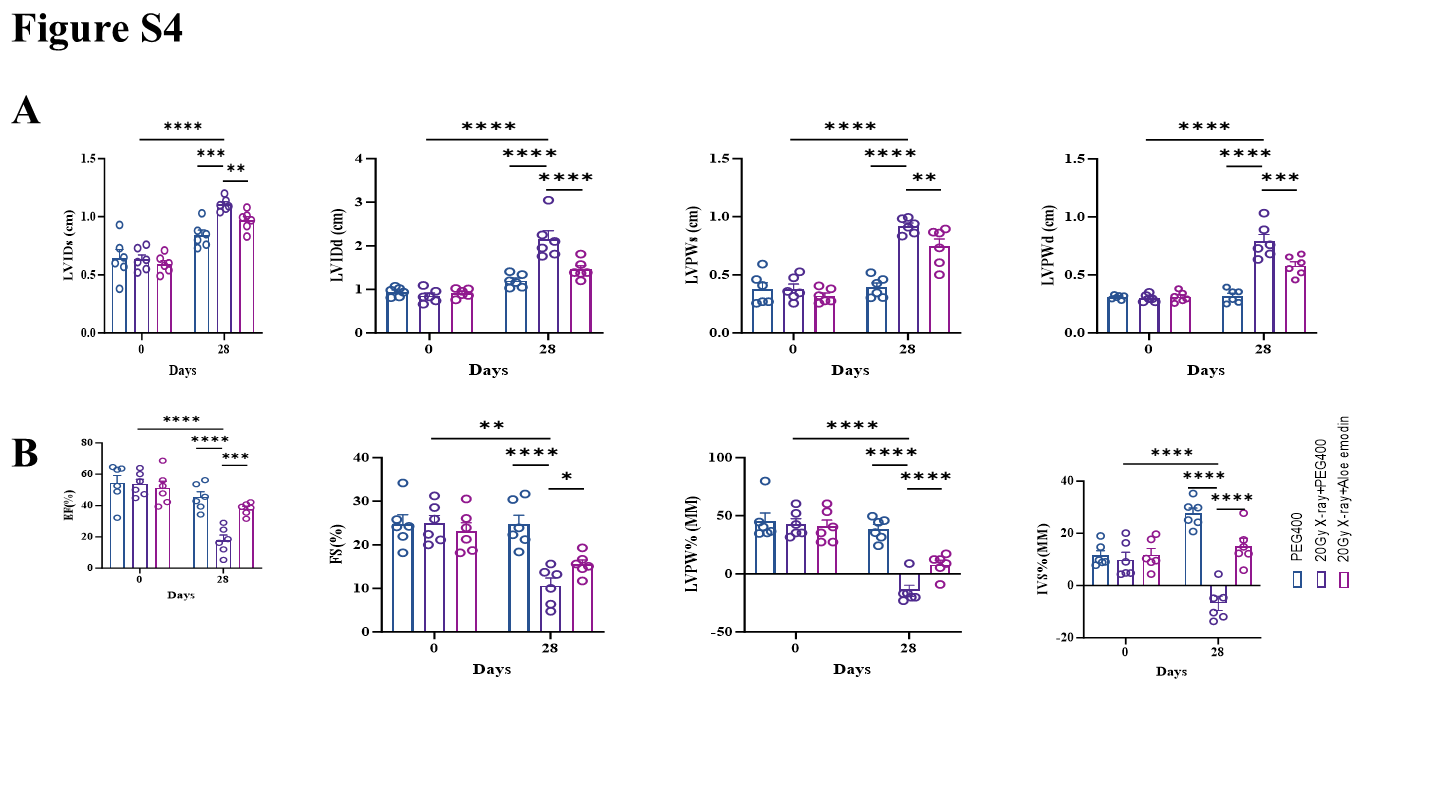


**Figure S4. A, B)** Quantification data for echocardiography detection of rats in 0-day or 28-day after X-ray or aloe emodin treatment. Quantification data are shown as mean ± SEM. All data were obtained from 6 independent experiments. *P* values were calculated by unpaired two-tail Student’s *t*-test or one-way ANOVA. **P* < 0.05; ***P* < 0.01; ****P* < 0.001; *****P* < 0.0001; ns: not significant.

**
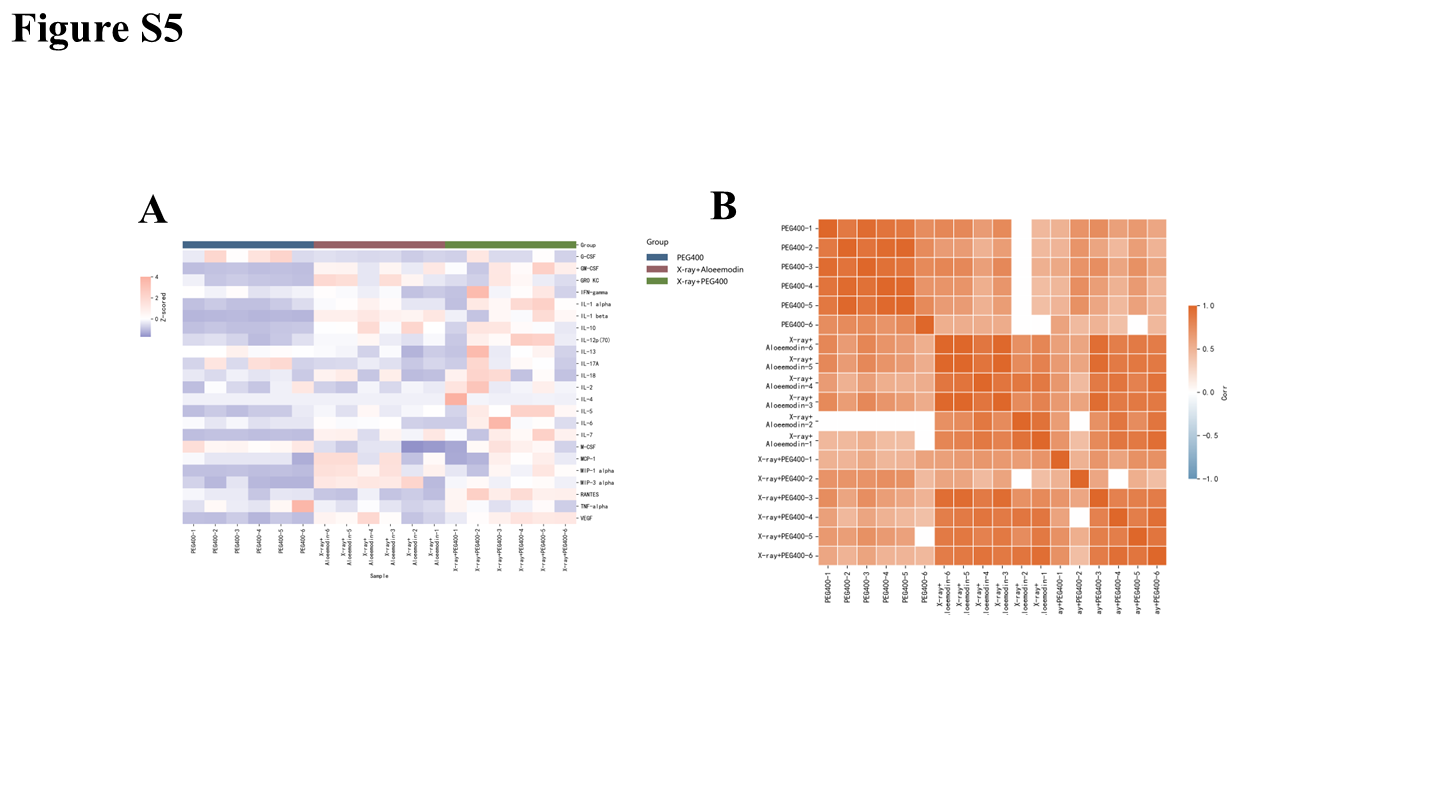
**

**Figure S5.** A) Cluster heatmap assay for 23 inflammatory factors in rat serum samples. B) Correlation analysis among sample in rat serum samples.

**
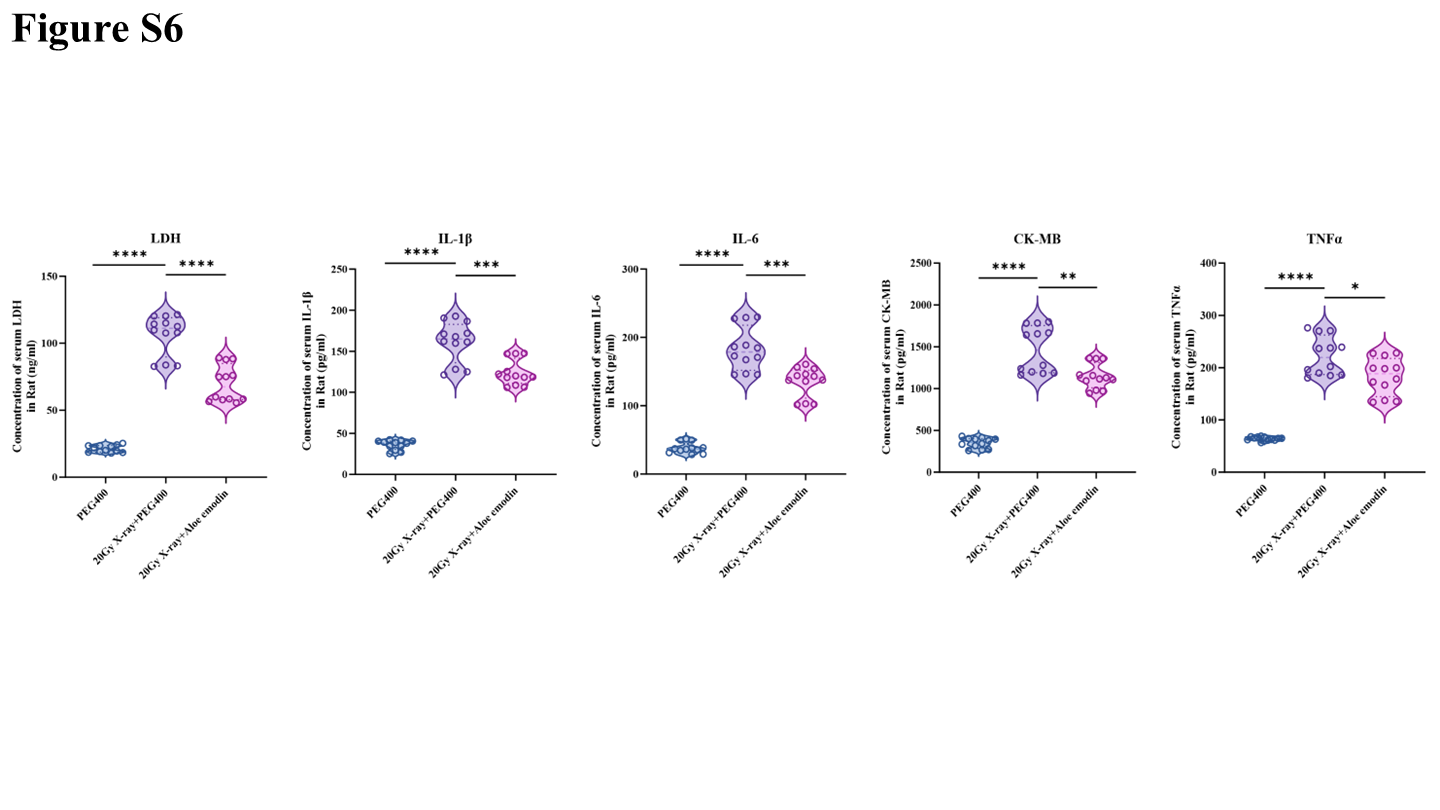
**

**Figure S6.** Aloe emodin alleviate radiation-induced heart damage by inhibiting inflammatory cascade. ELISA assay of concentrations of LDH, CK-MB, IL-1β, IL-6 and TNFα in rat serum samples after aloe emodin treatment. Quantification data are shown as mean ± SEM. All data were obtained from 12 independent experiments. *P* values were calculated by unpaired two-tail Student’s *t*-test or one-way ANOVA. **P* < 0.05; ***P* < 0.01; ****P* < 0.001; *****P* < 0.0001.

**
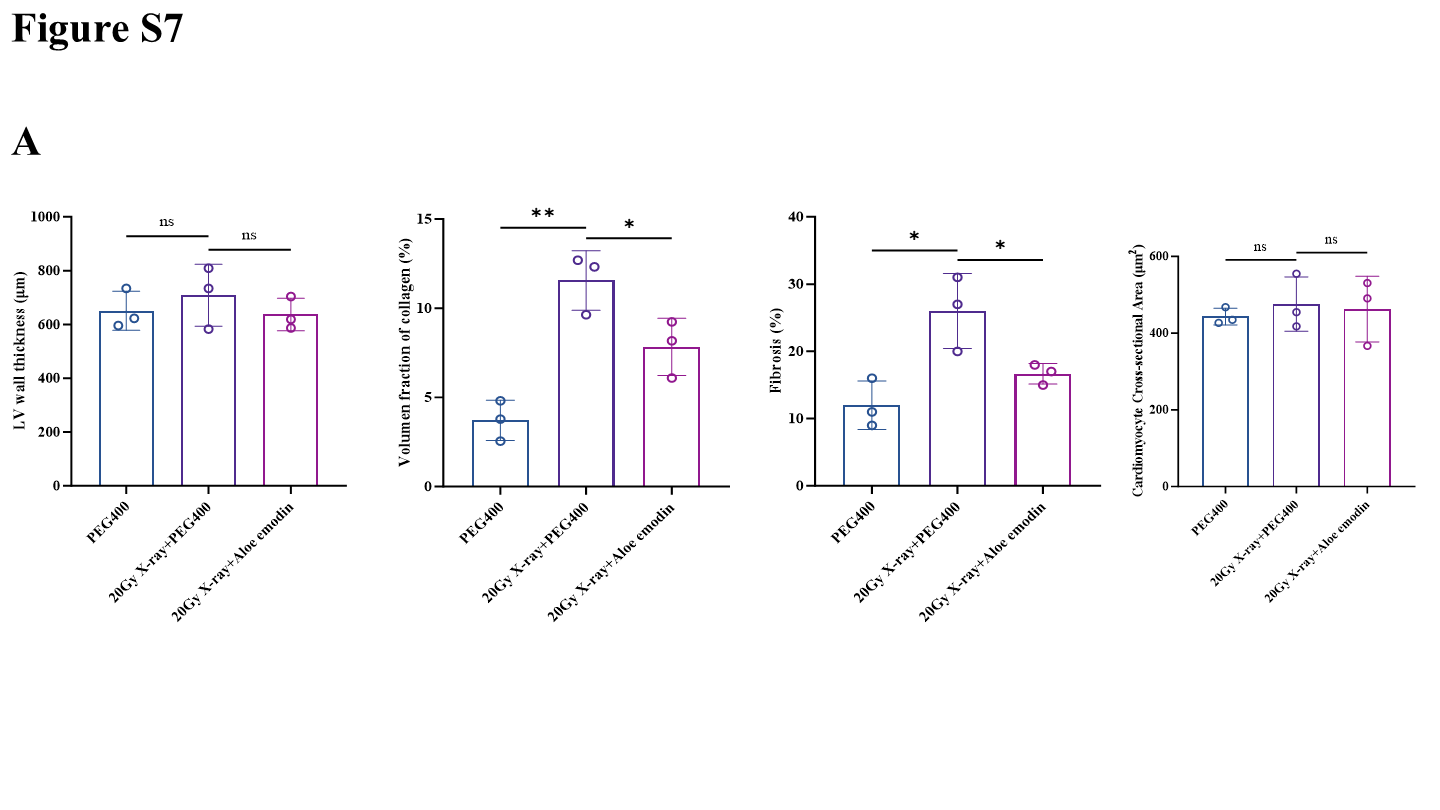
**

**Figure S7.** Quantification data for morphological detection and staining evaluation of rat heart, including LV wall thickness, Volume fraction of collagen (%), Fibrosis (%) and Cardiomyocyte cross-sectional area. Quantification data are shown as mean ± SEM. All data were obtained from 3 independent experiments. *P* values were calculated by unpaired two-tail Student’s *t*-test or one-way ANOVA. **P* < 0.05; ***P* < 0.01; ****P* < 0.001; *****P* < 0.0001; ns: not significant.


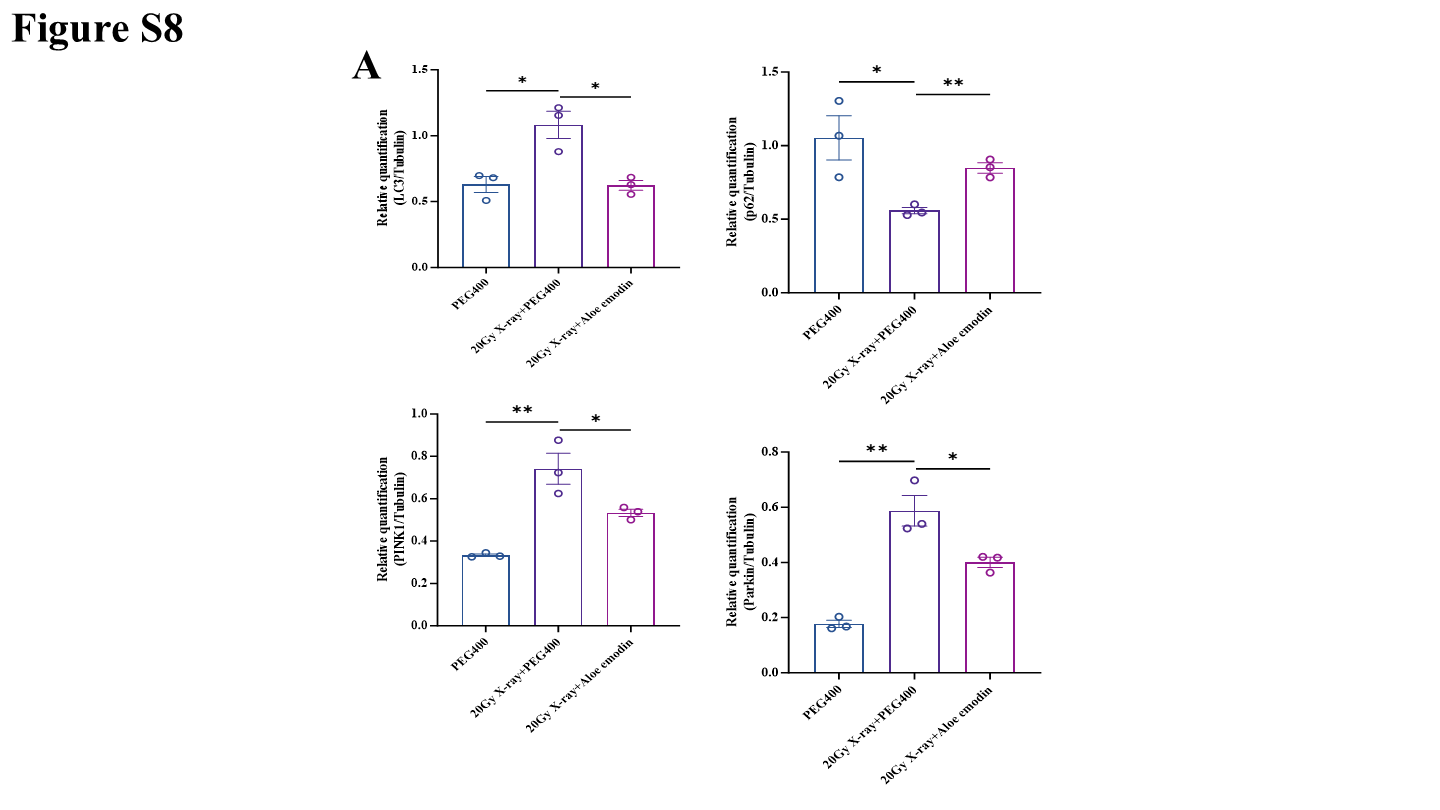


**Figure S8.** Quantification data for mitophagy-related protein levels in rat heart tissues, including LC3, p62, PINK1 and Parkin, were determined using Western blotting. Quantification data are shown as mean ± SEM. All data were obtained from 3 or 6 independent experiments. *P* values were calculated by unpaired two-tail Student’s *t*-test or one-way ANOVA. **P* < 0.05; ***P* < 0.01; ****P* < 0.001; *****P* < 0.0001; ns: not significant.


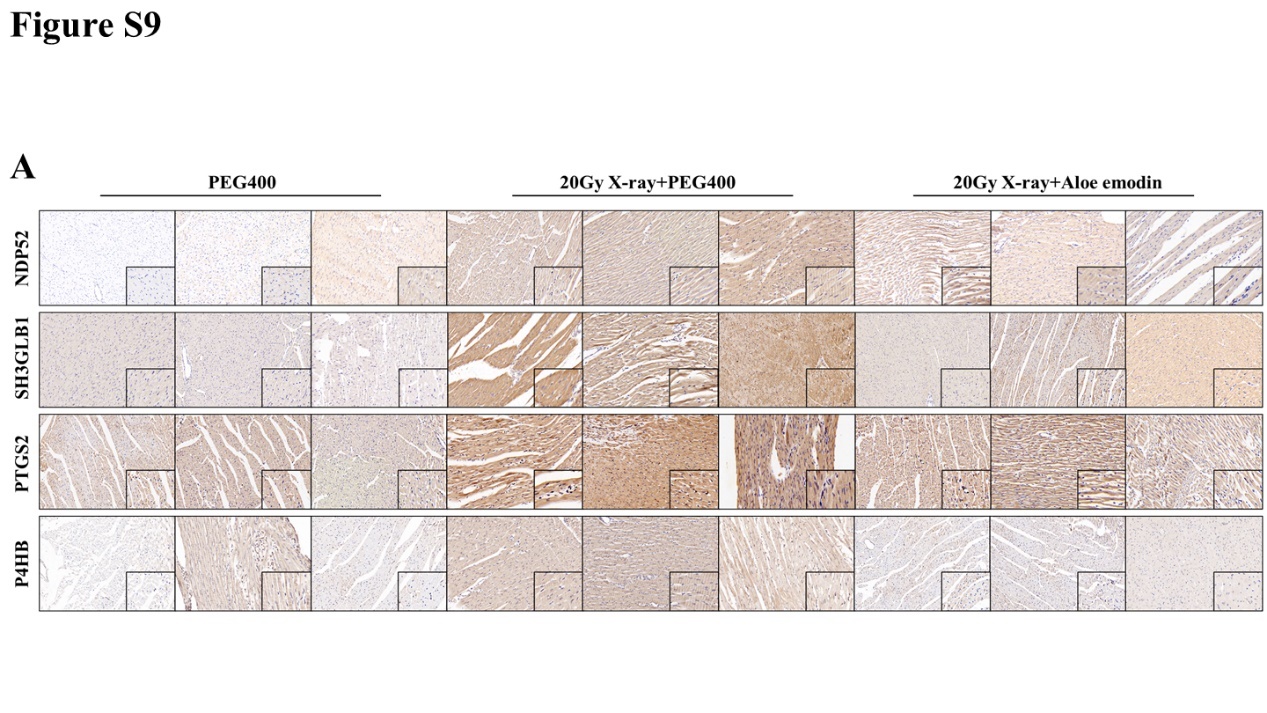


**Figure S9.** IHC staining and detection of NDP52, SH3GLB1, PTGS2 and P4HB in sections of rat heart. Scale bar, 40μm and 20μm.


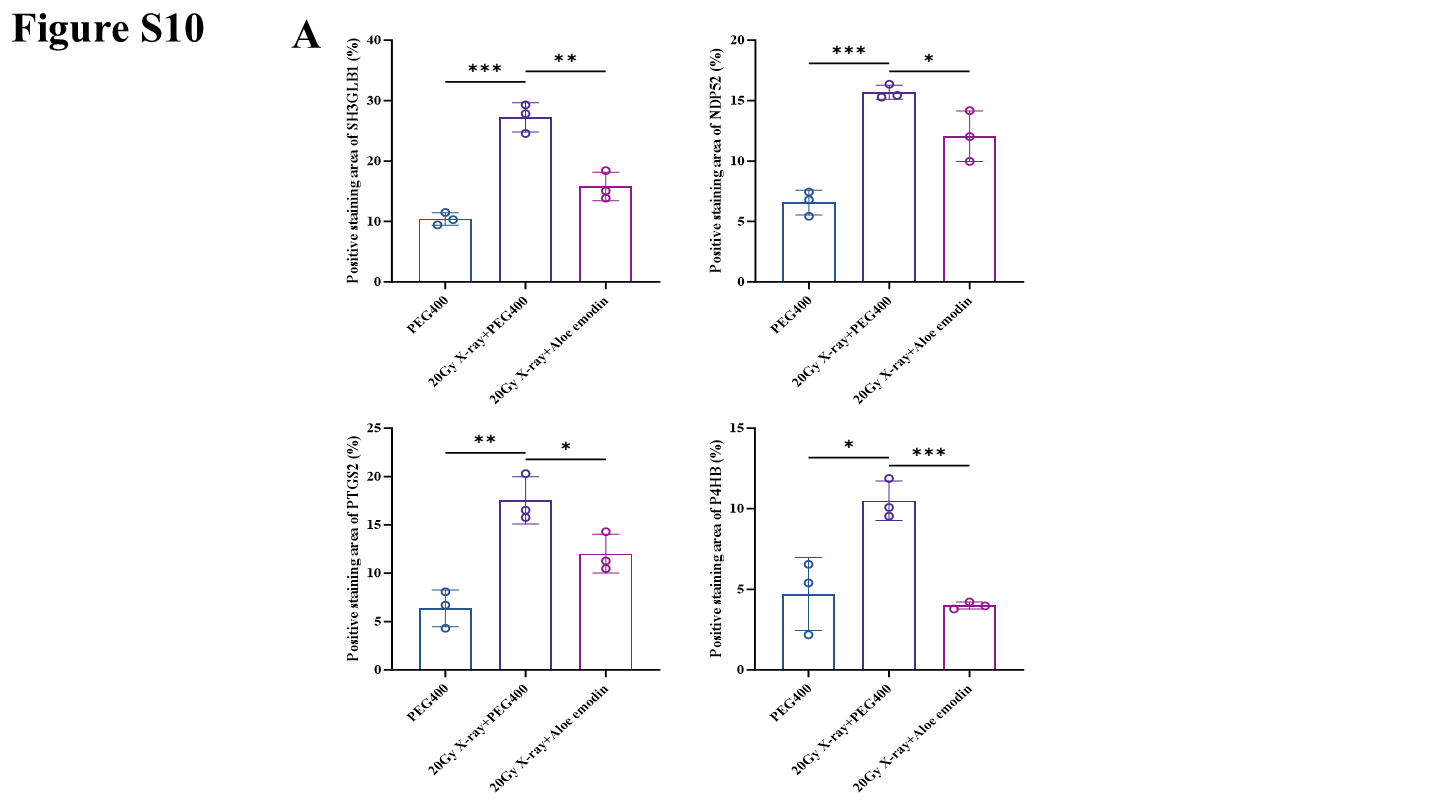


**Figure S10.** Quantification data for IHC staining and detection of NDP52, SH3GLB1, PTGS2 and P4HB in sections of rat heart. Quantification data are shown as mean ± SEM. All data were obtained from 3 independent experiments. *P* values were calculated by unpaired two-tail Student’s *t*-test or one-way ANOVA. **P* < 0.05; ***P* < 0.01; ****P* < 0.001; *****P* < 0.0001; ns: not significant.


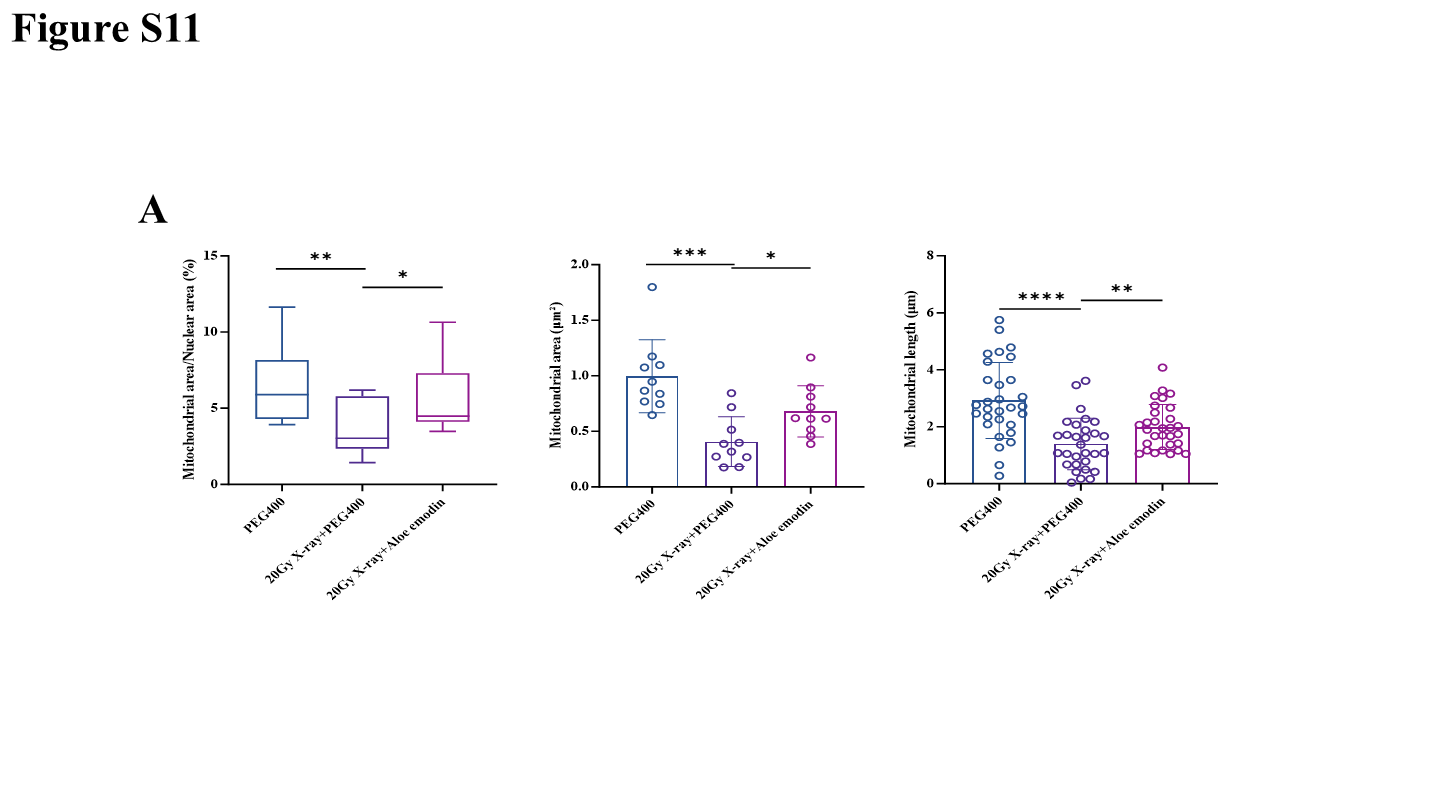


**Figure S11.** Quantification data for TEM analysis of cardiac tissue of rat heart, red arrow for mitochondrial. Quantification data are shown as mean ± SEM. All data were obtained from 3 independent experiments. *P* values were calculated by unpaired two-tail Student’s *t*-test or one-way ANOVA. **P* < 0.05; ***P* < 0.01; ****P* < 0.001; *****P* < 0.0001; ns: not significant.


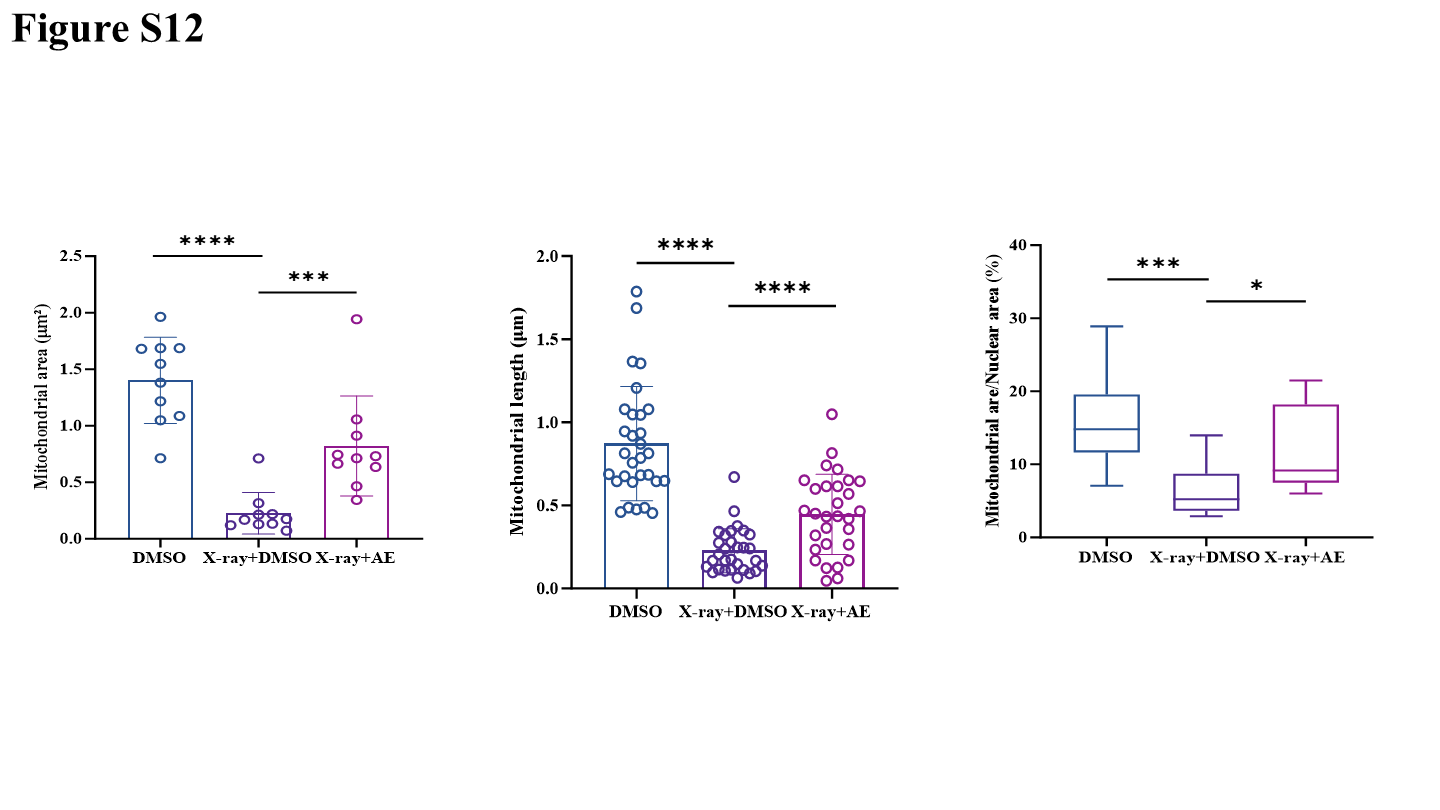


**Figure S12.** Quantification data for TEM analysis of H9C2 cells after X-ray or aloe emodin treatment. Quantification data are shown as mean ± SEM. All data were obtained from 3 independent experiments. *P* values were calculated by unpaired two-tail Student’s *t*-test or one-way ANOVA. **P* < 0.05; ****P* < 0.001; *****P* < 0.0001; ns: not significant.


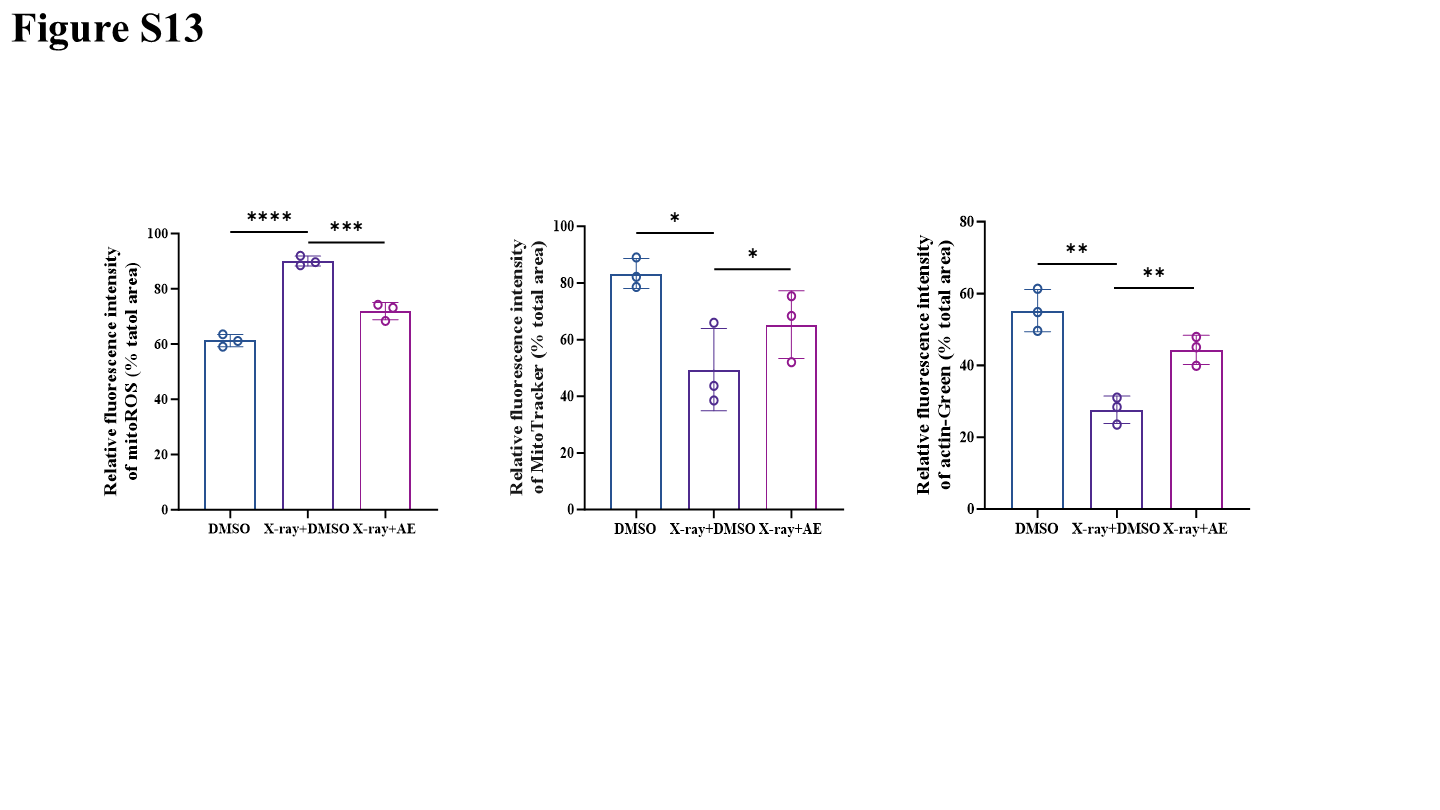


**Figure S13.** Quantification data for mitochondrial ROS, mitochondrial morphology, and cardiac cytoskeleton and morphology were detected by mtSOX, mitoTracker Green and actin-Tracker Green-488, respectively. Quantification data are shown as mean ± SEM. All data were obtained from 3 independent experiments. *P* values were calculated by unpaired two-tail Student’s *t*-test or one-way ANOVA. **P* < 0.05; ***P* < 0.01; ****P* < 0.001; *****P* < 0.0001.


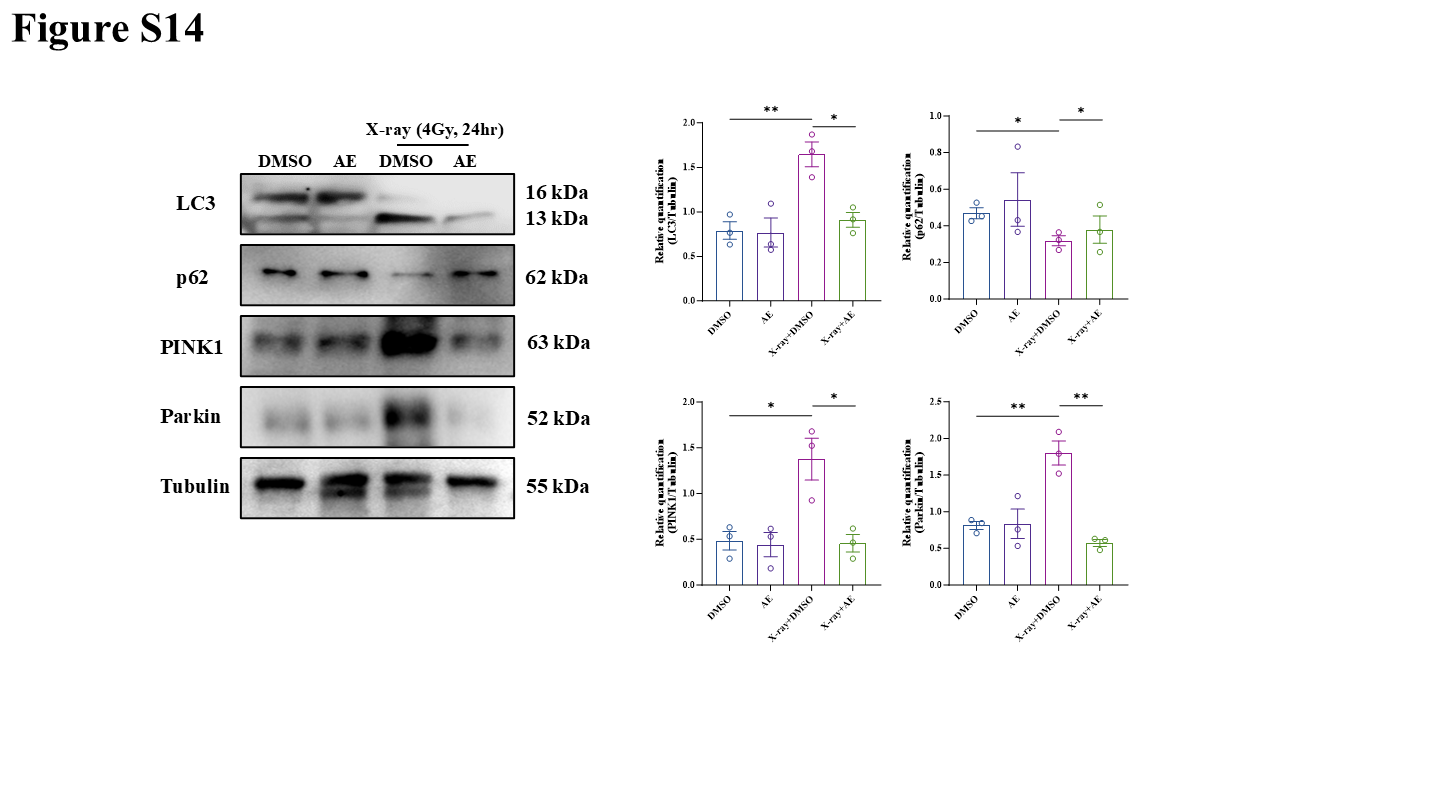


**Figure S14.** Quantification data for mitophagy-related protein levels in H9C2 cells, including LC3, p62, PINK1 and Parkin, were determined using Western blotting. Quantification data are shown as mean ± SEM. All data were obtained from 3 independent experiments. *P* values were calculated by unpaired two-tail Student’s *t*-test or one-way ANOVA. **P* < 0.05; ***P* < 0.01.


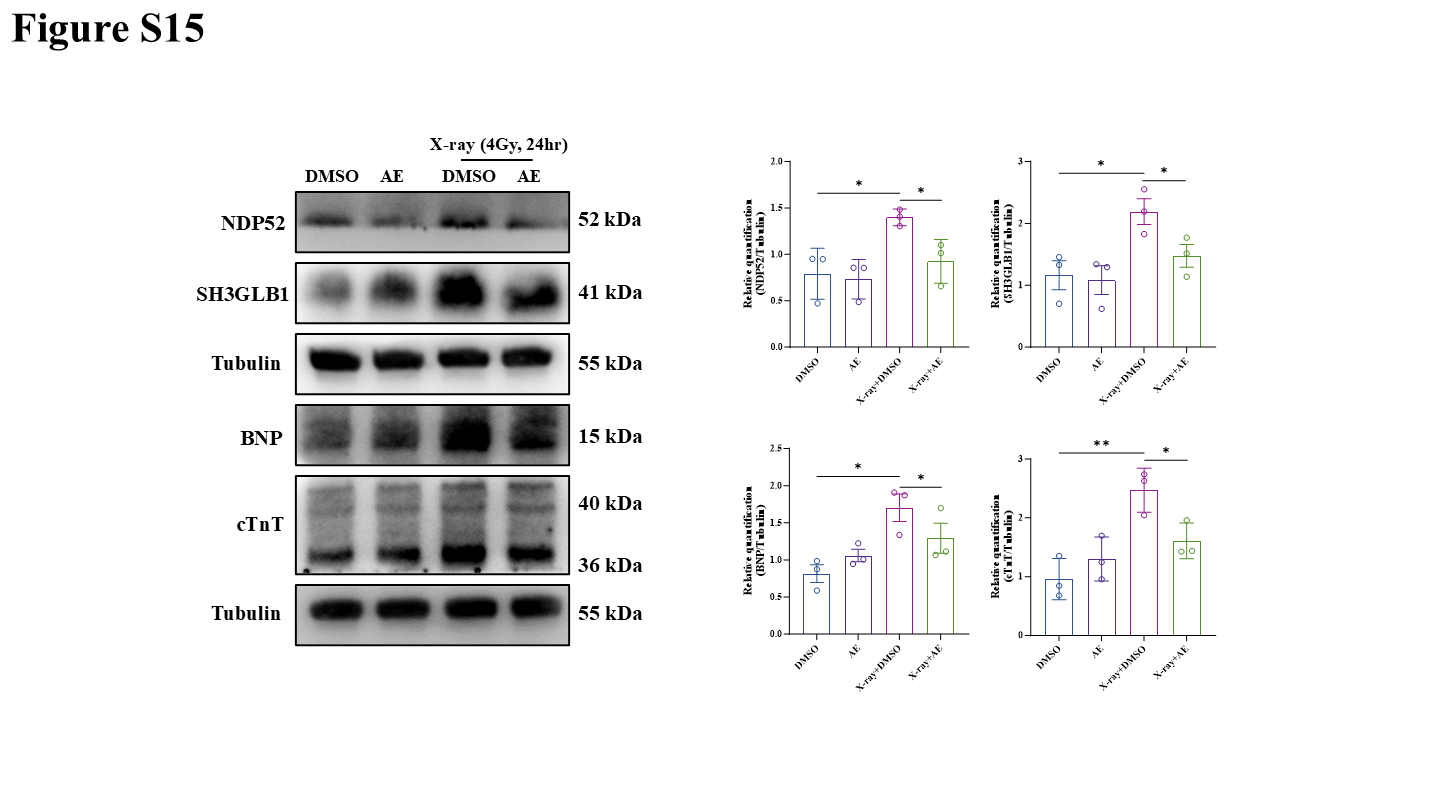


**Figure S15.** Cardiac injury-related protein BNP and cTnT, and protein levels of NDP52 and SH3GLB1, were determined using Western blotting. Quantification data are shown as mean ± SEM. All data were obtained from 3 independent experiments. *P* values were calculated by unpaired two-tail Student’s *t*-test or one-way ANOVA. **P* < 0.05; ***P* < 0.01.


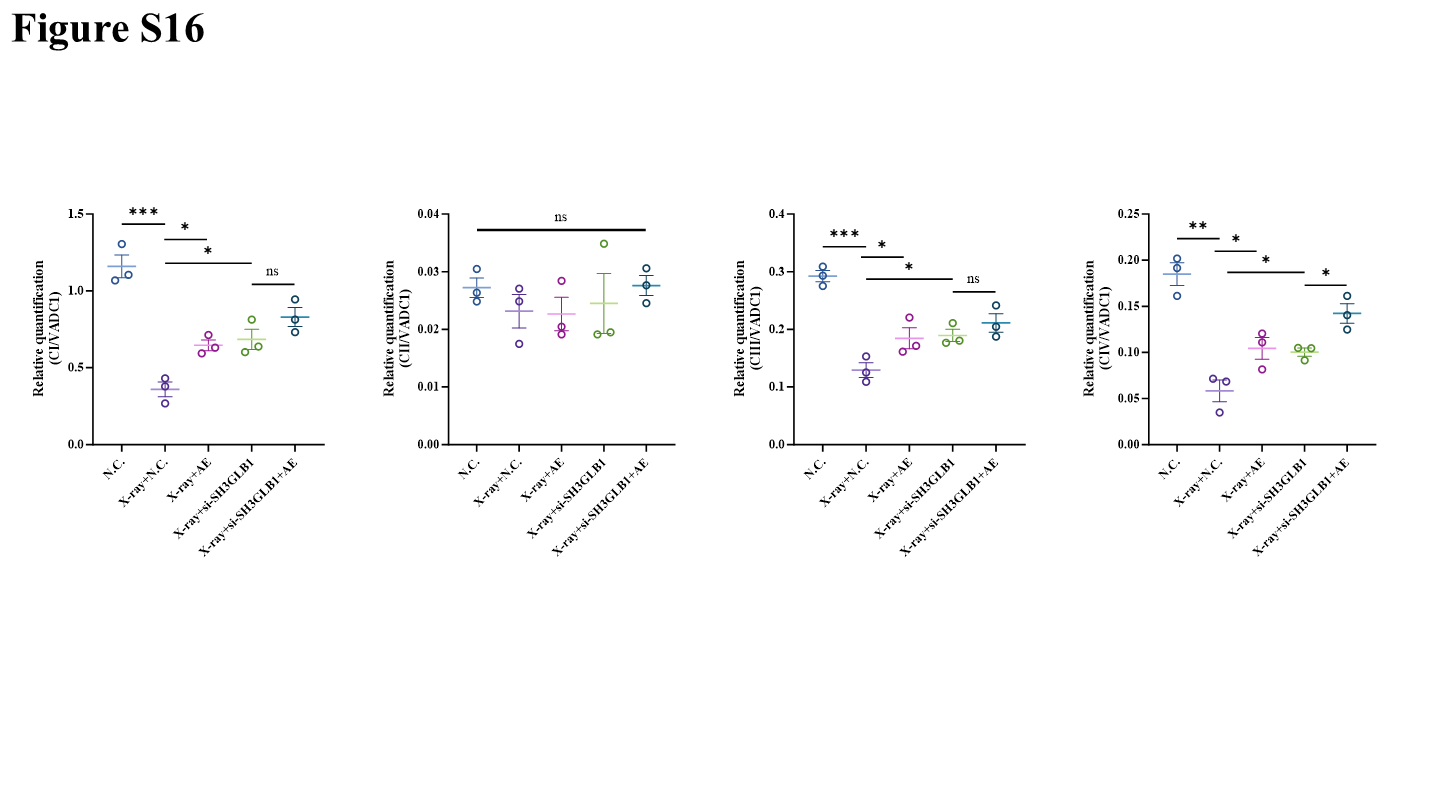


**Figure S16.** Quantification data for Western blotting detection of mitochondrial complexes, including complex I, II, III, IV and V, using mixture of 5 antibodies, after mitochondrial dissociation. Quantification data are shown as mean ± SEM. All data were obtained from 3 independent experiments. *P* values were calculated by unpaired two-tail Student’s *t*-test or one-way ANOVA. **P* < 0.05; ***P* < 0.01.


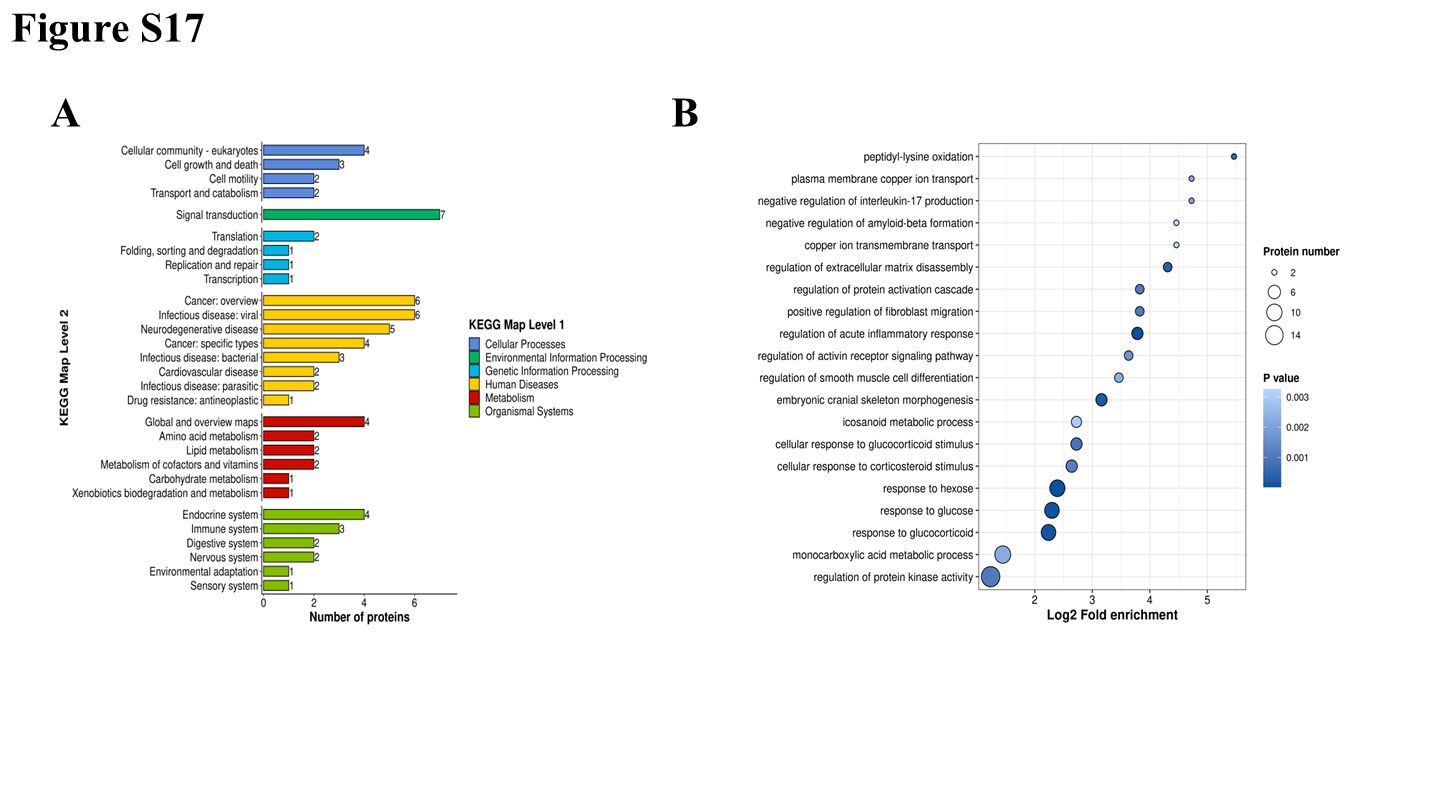


**Figure S17.** Differential protein omics analysis of upregulation of PTGS2 (P35355), and enrichment of PTGS2 in cardiovascular disease and positive regulation of activation cascade by KEGG and GO.

**
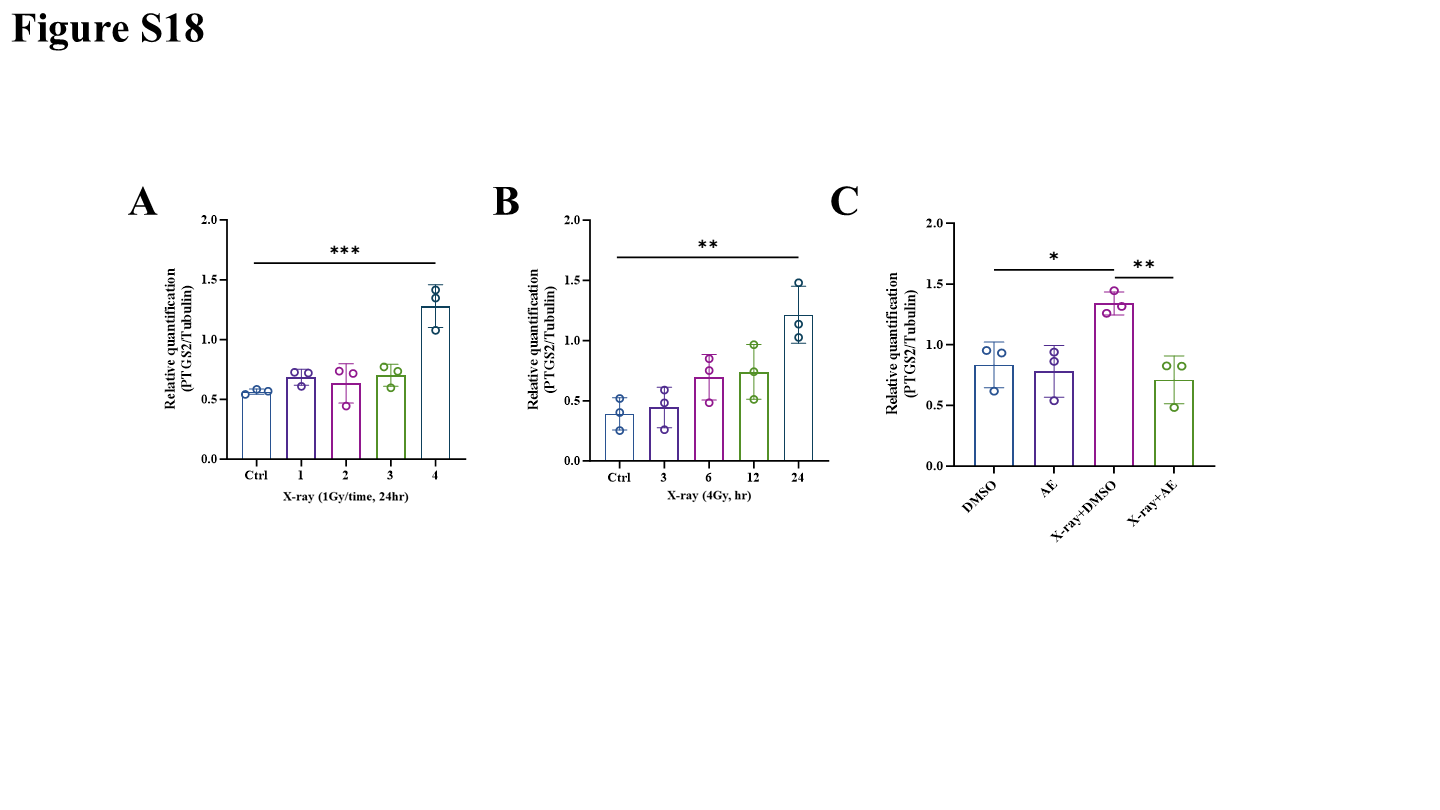
**

**Figure S18. A, B)** Quantification data for Western blotting detection of PTGS2 level by X-ray in a dose- and time-dependent manner. **C)** Quantification data for Western blotting detection of PTGS2 level by aloe emodin treatment. Quantification data are shown as mean ± SEM. All data were obtained from 3 independent experiments. *P* values were calculated by unpaired two-tail Student’s *t*-test or one-way ANOVA. **P* < 0.05; ***P* < 0.01; ****P* < 0.001.


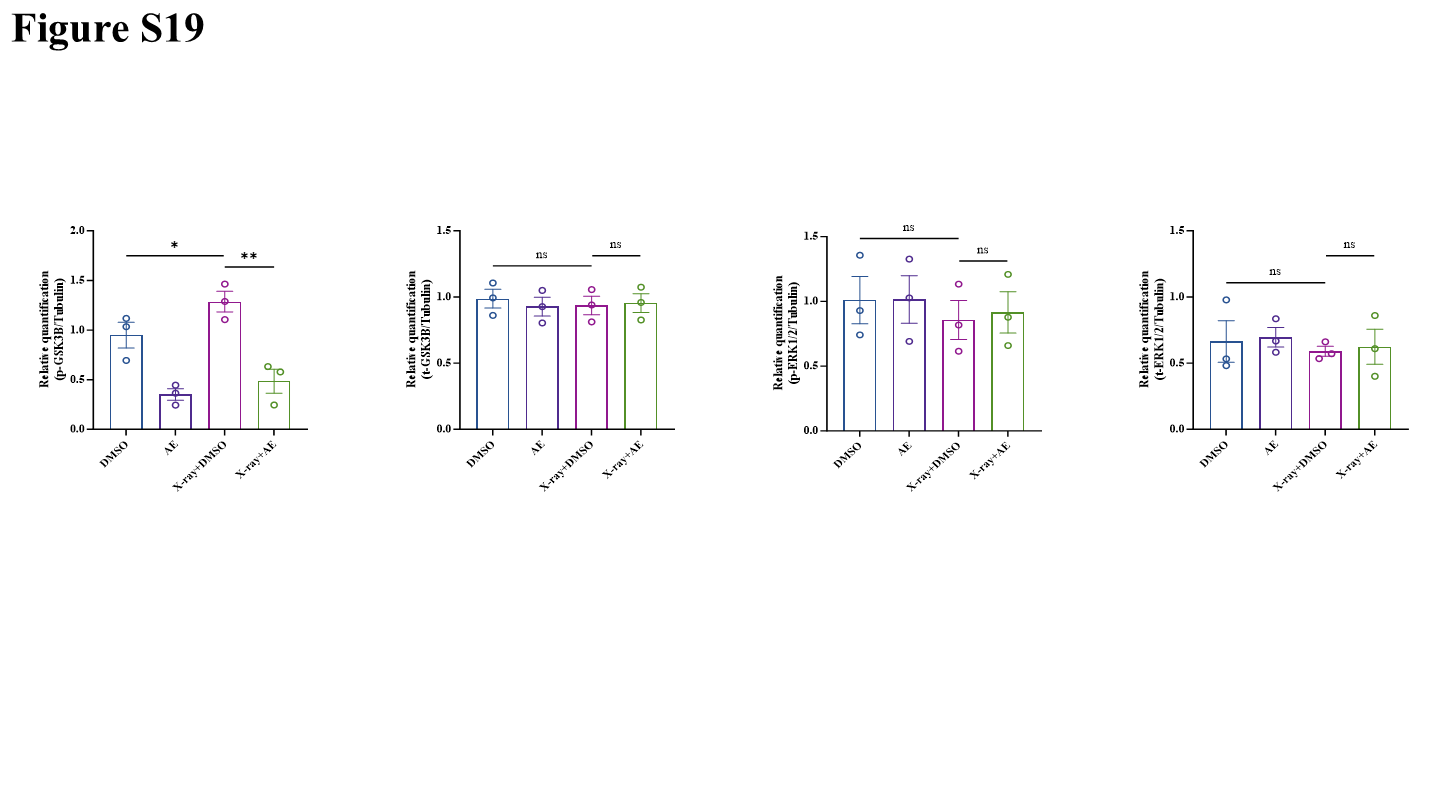


**Figure S19.** Quantification data for Western blotting detection of p-GSK3B, t-GSK3B, p-ERK1/2 and t-ERK1/2 by aloe emodin treatment. Quantification data are shown as mean ± SEM. All data were obtained from 3 independent experiments. *P* values were calculated by unpaired two-tail Student’s *t*-test or one-way ANOVA. **P* < 0.05; ***P* < 0.01; ns: not significant.

**
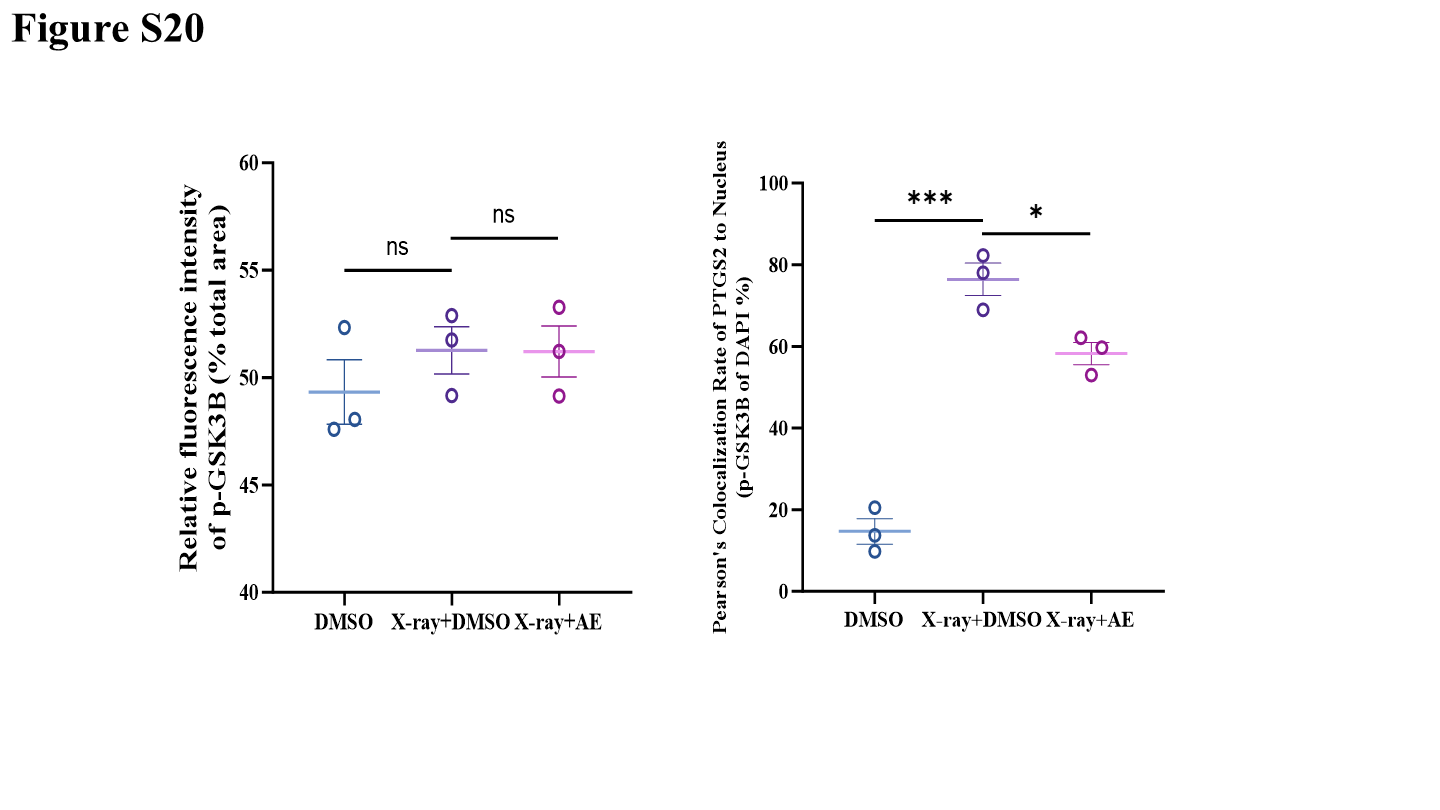
**

**Figure S20.** Relative fluorescence intensity and Pearson’s colocalization rate of PTGS2 to nucleus by aloe emodin treatment in H9C2. Quantification data are shown as mean ± SEM. All data were obtained from 3 independent experiments. *P* values were calculated by unpaired two-tail Student’s *t*-test or one-way ANOVA. **P* < 0.05; ****P* < 0.001; ns: not significant.

**
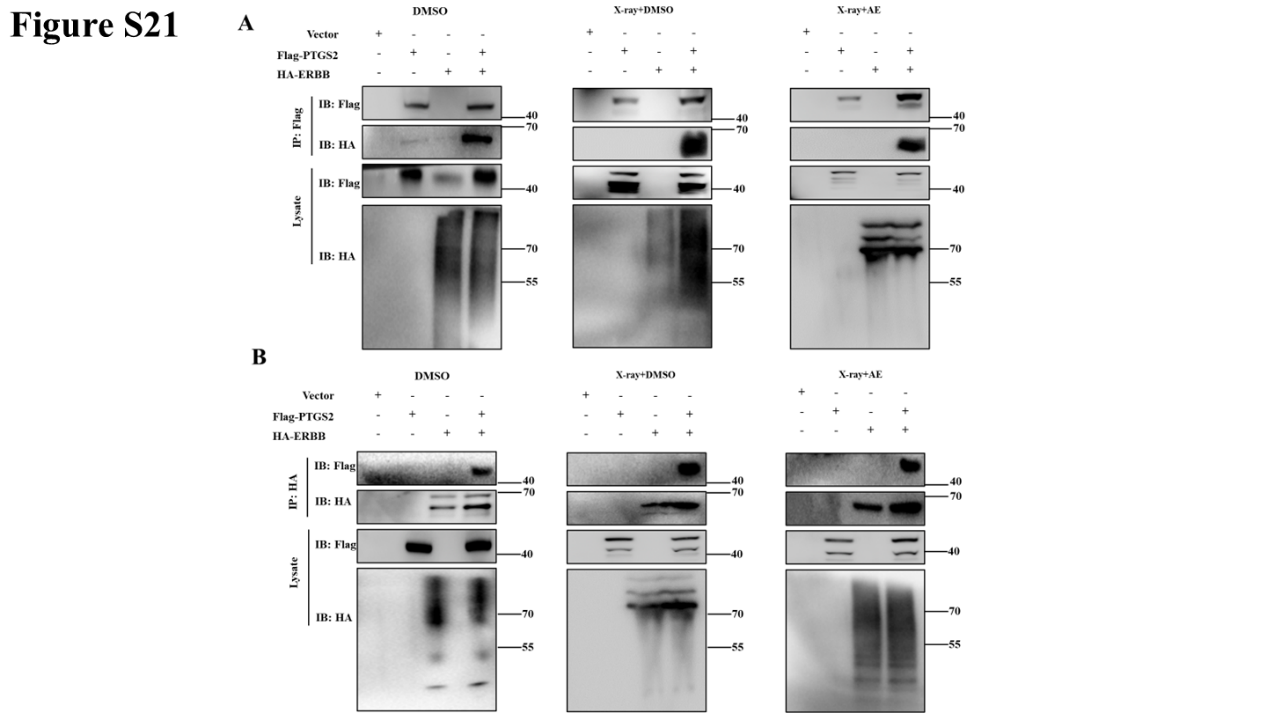
**

**Figure S21.** Inhibition of interaction between PTGS2 and ERBB2 by aloe emodin treatment was detected by co-immunoprecipitation after transfecting Flag-PTGS2 and HA-ERBB into HEK-293T cells.

**
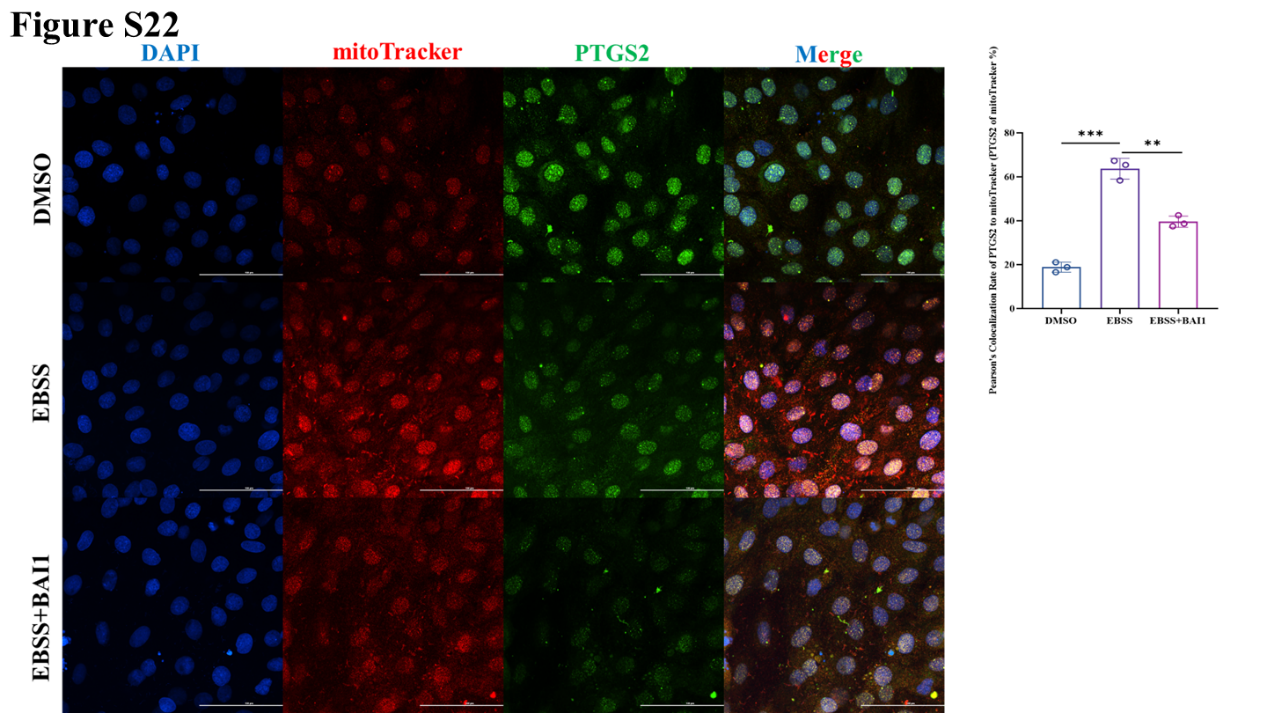
**

**Figure S22.** Co-localization of PTGS2 to mitochondrial under treatment with autophagy inducer EBSS or BAX inhibitor BAI1, detecting by laser-confocal. Scale bar, 100μm. All data were obtained from 3 independent experiments. *P* values were calculated by unpaired two-tail Student’s *t*-test or one-way ANOVA. ***P* < 0.01; ****P* < 0.001.


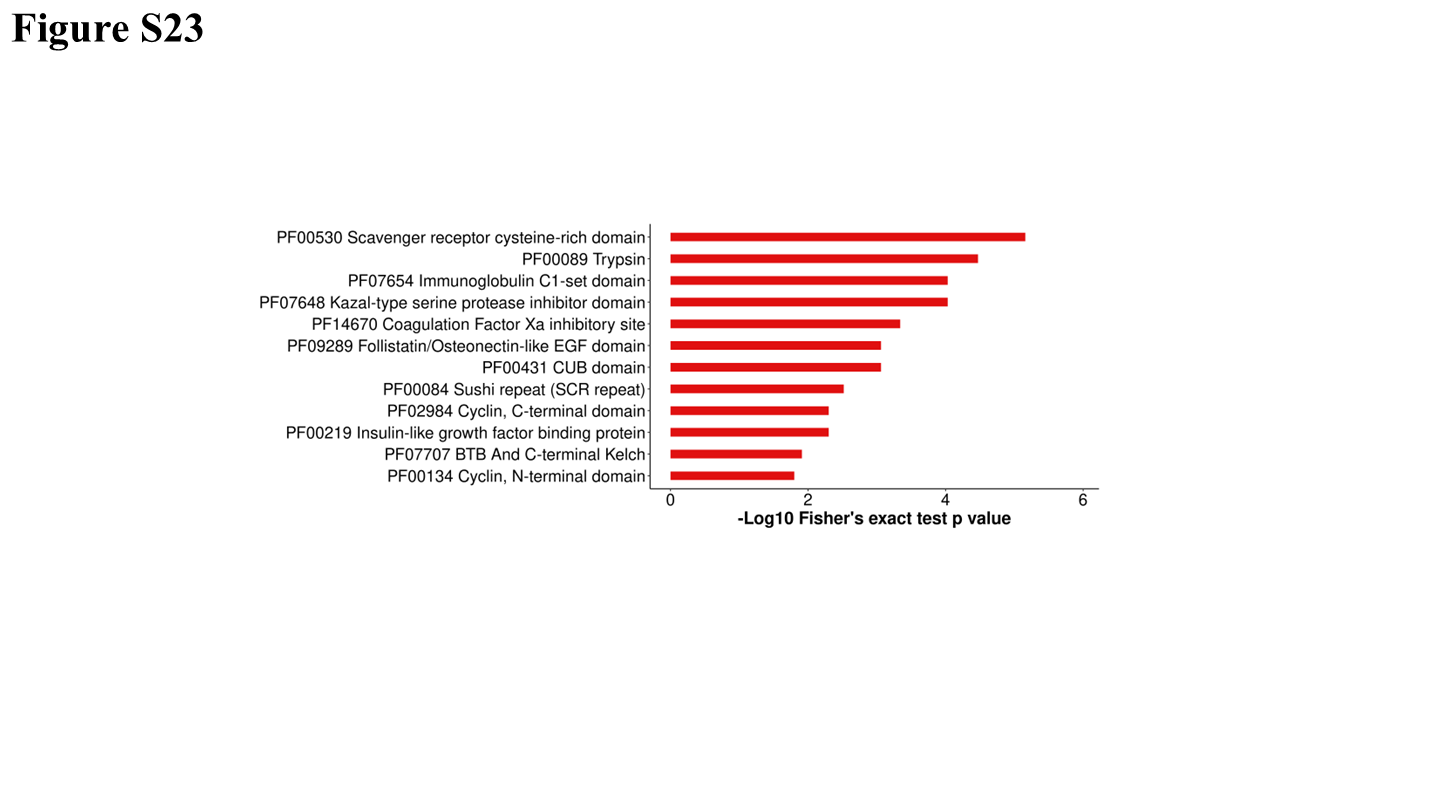


**Figure S23.** Fisher’s exact test *P* value for the structural domain of selected proteins.


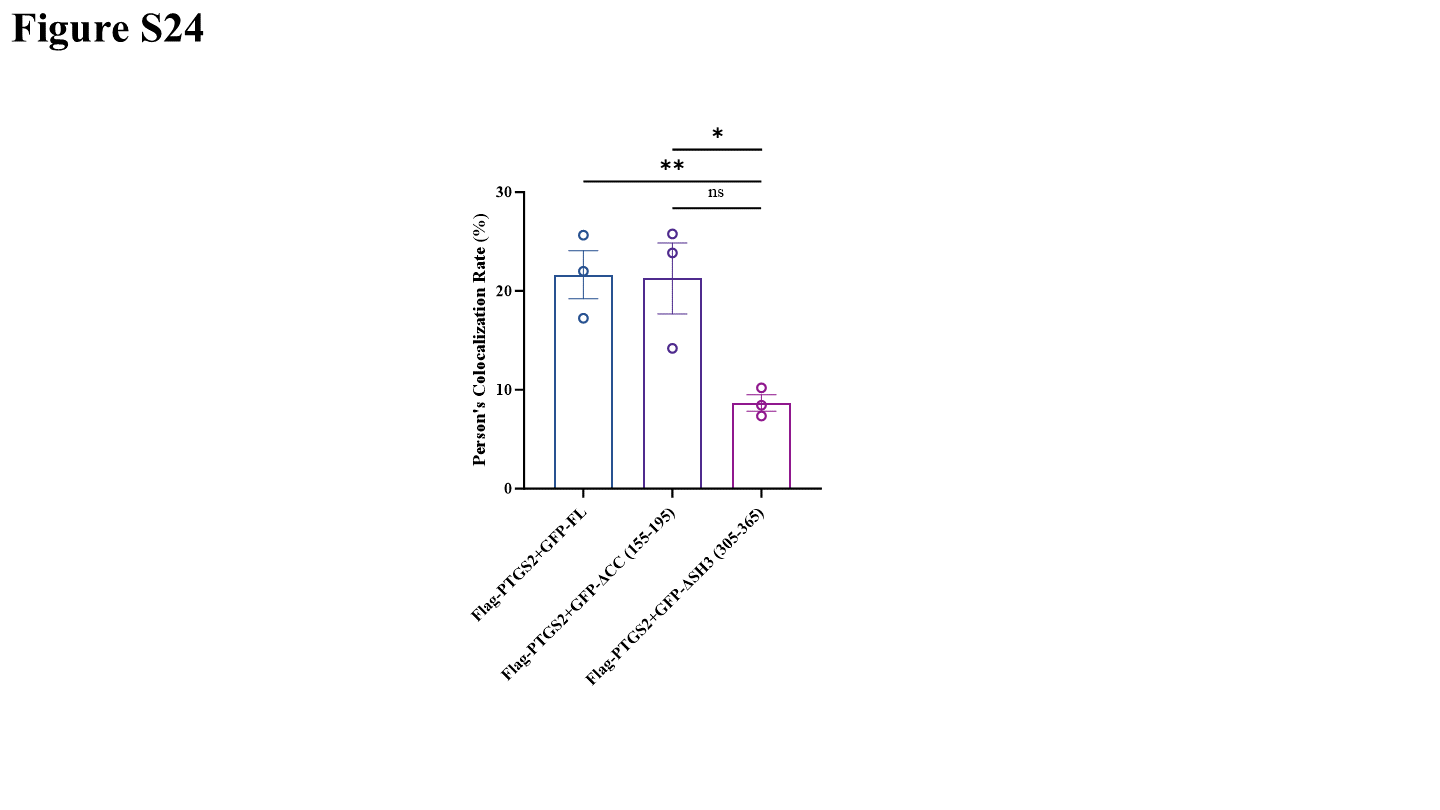


**Figure S24.** Pearson’s colocalization rate of PTGS2 and SH3GLB1 after transfection of GFP-FL, GFP-△CC and GFP-△SH3 in the context of endogenous SH3GLB1 deletion in H9C2 cells. Quantification data are shown as mean ± SEM. All data were obtained from 3 independent experiments. *P* values were calculated by unpaired two-tail Student’s *t*-test or one-way ANOVA. **P* < 0.05; ***P* < 0.01; ns: not significant.


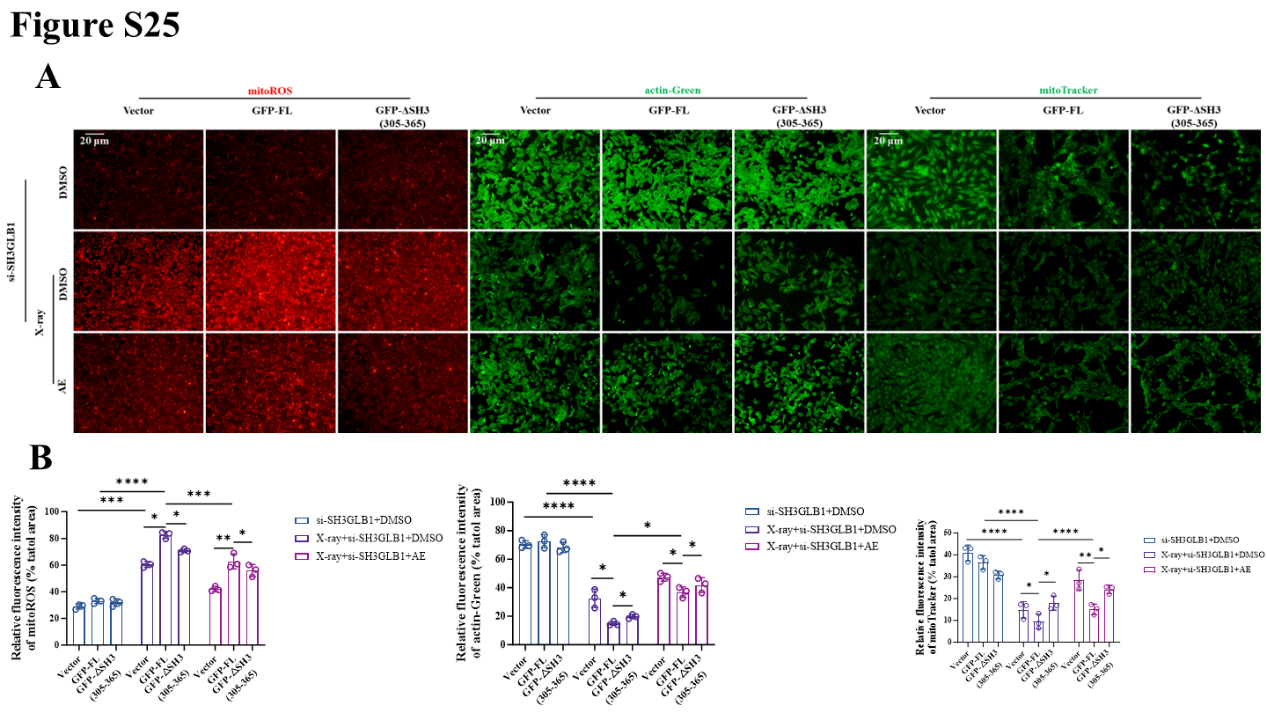


**Figure S25.** Mitochondrial ROS, mitochondrial morphology, and cardiac cytoskeleton and morphology were detected by mtSOX, mitoTracker and actin-Green after transfection of GFP-FL, GFP-△CC and GFP-△SH3 in the context of endogenous SH3GLB1 deletion in H9C2 cells. Scale bar, 20μm. Quantification data are shown as mean ± SEM. All data were obtained from 3 independent experiments. *P* values were calculated by unpaired two-tail Student’s *t*-test or one-way ANOVA. **P* < 0.05; ***P* < 0.01; ****P* < 0.001; *****P* < 0.0001; ns: not significant.


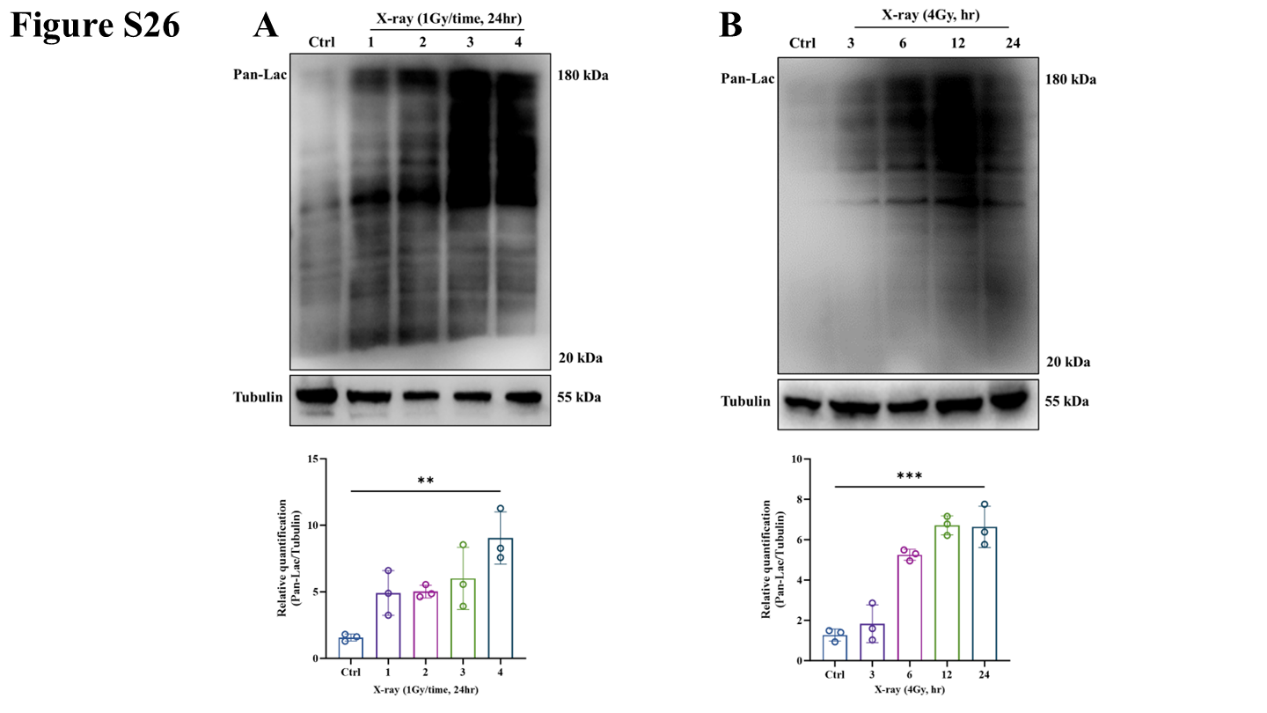


**Figure S26.** A, B) Upregulation of protein levels of Pan-Lac by X-ray in a dose- and time-dependent manner, determined using Western blotting. Quantification data are shown as mean ± SEM. All data were obtained from 3 independent experiments. *P* values were calculated by unpaired two-tail Student’s *t*-test or one-way ANOVA. ***P* < 0.01; ****P* < 0.001.


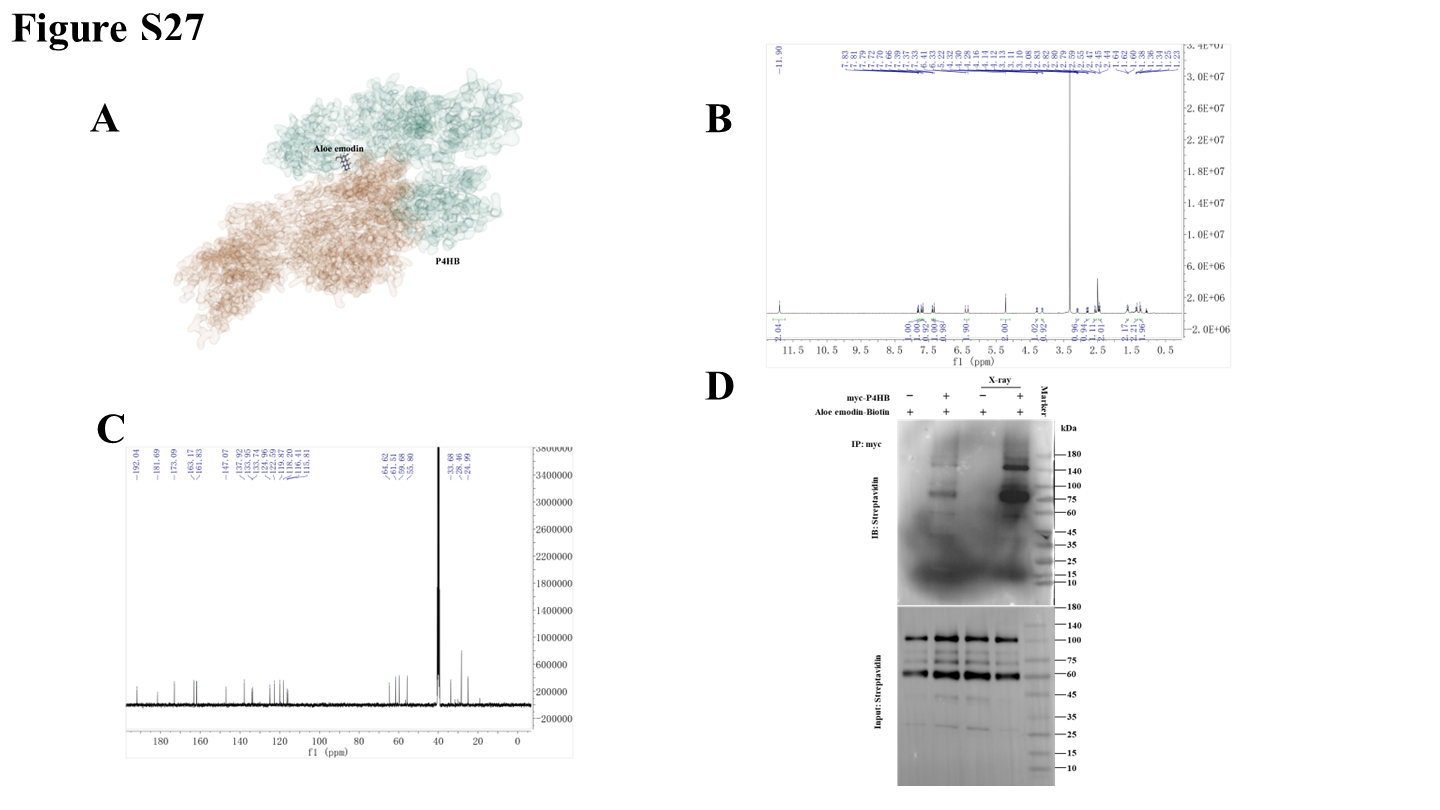


**Figure S27.** A) Binding mode of aloe emodin to P4HB by using Autodock Vina. B, C) ^1^H NMR of AE-Biotin in DMSO-*d6.* D). Co-IP and streptavidin incubation showed upregulated interaction between aloe emodin-biotin and myc-P4HB, determined using Western blotting.


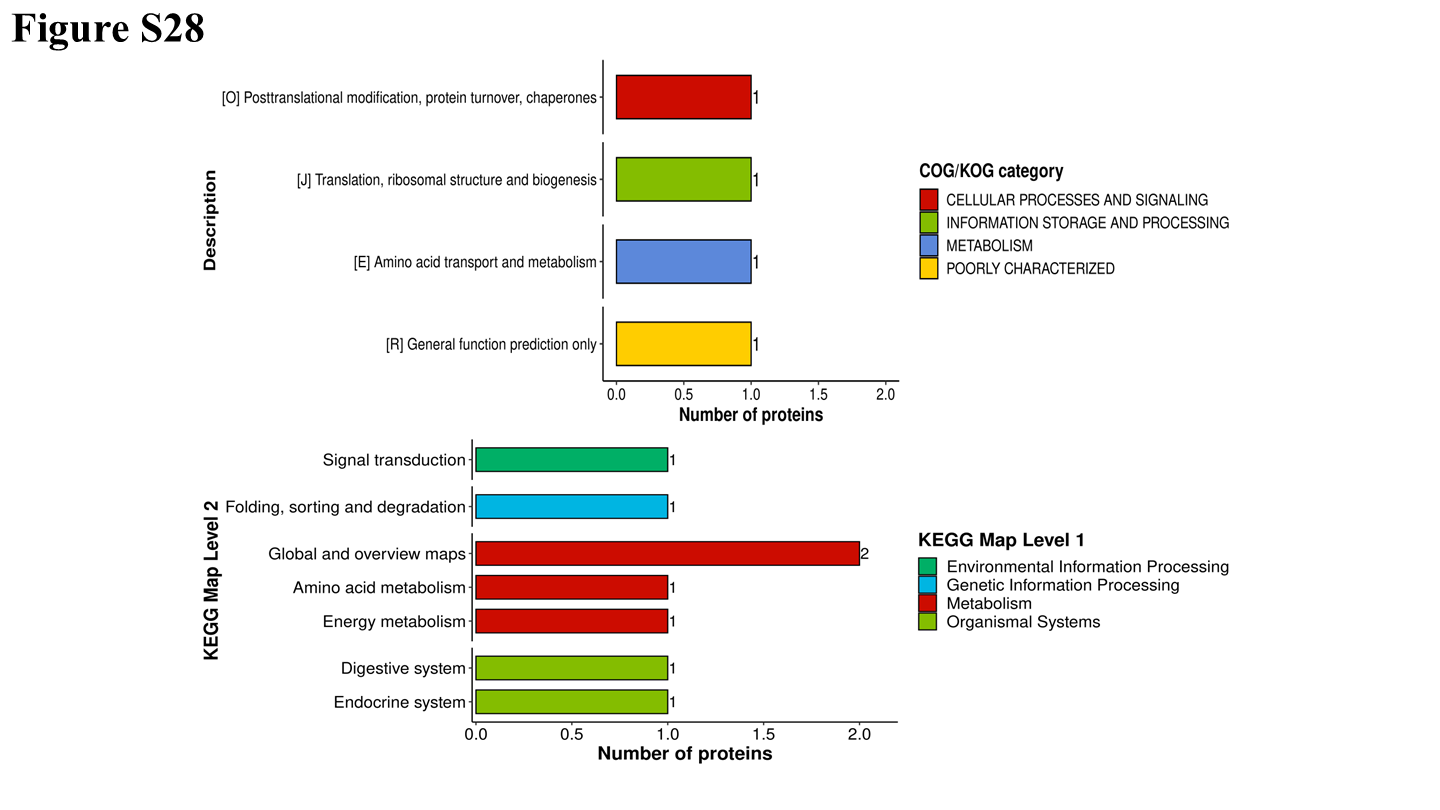


**Figure S28. Analysis of COG or KEGG for** lactylation omics of 4D label free on upregulation and modification for P4HB (P04785).


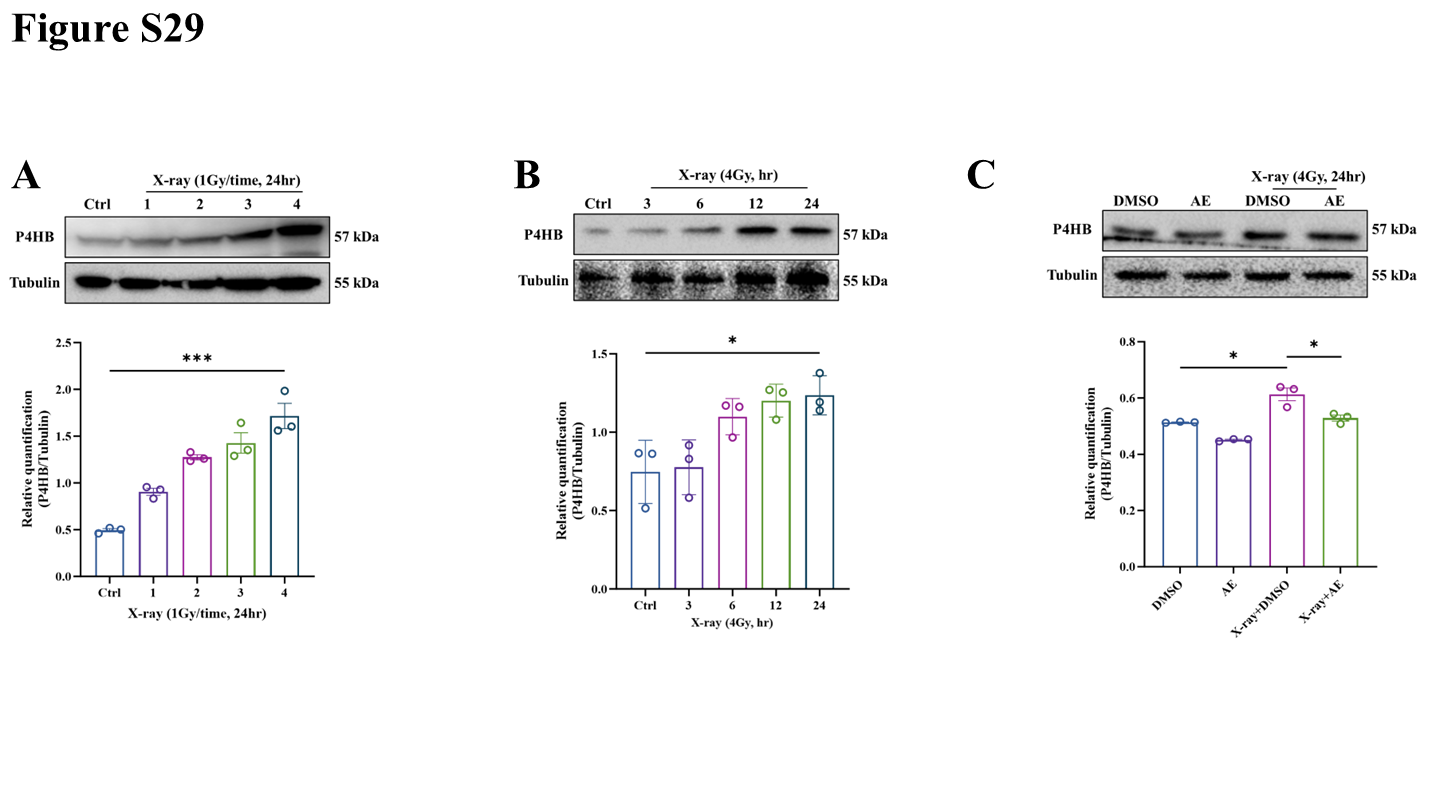


**Figure S29.** A, B) Upregulation of protein levels of P4HB by X-ray in a dose- and time-dependent manner, were determined using Western blotting. C) Downregulation of P4HB level by aloe emodin treatment detecting by Western blotting. Quantification data are shown as mean ± SEM. All data were obtained from 3 independent experiments. *P* values were calculated by unpaired two-tail Student’s *t*-test or one-way ANOVA. **P* < 0.05; ****P* < 0.001.


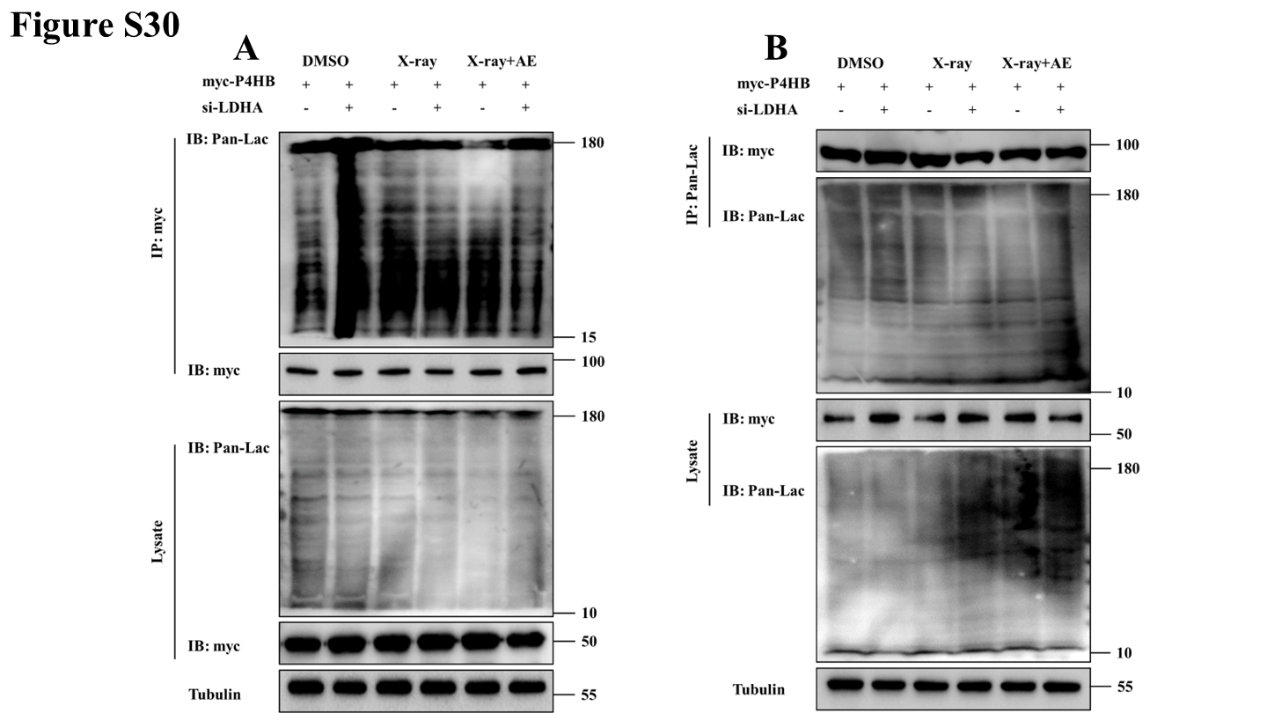


**Figure S30.** A, B) Co-immunoprecipitation assay of interaction between P4HB and Pac-Lac in the context of aloe emodin and si-LDHA.


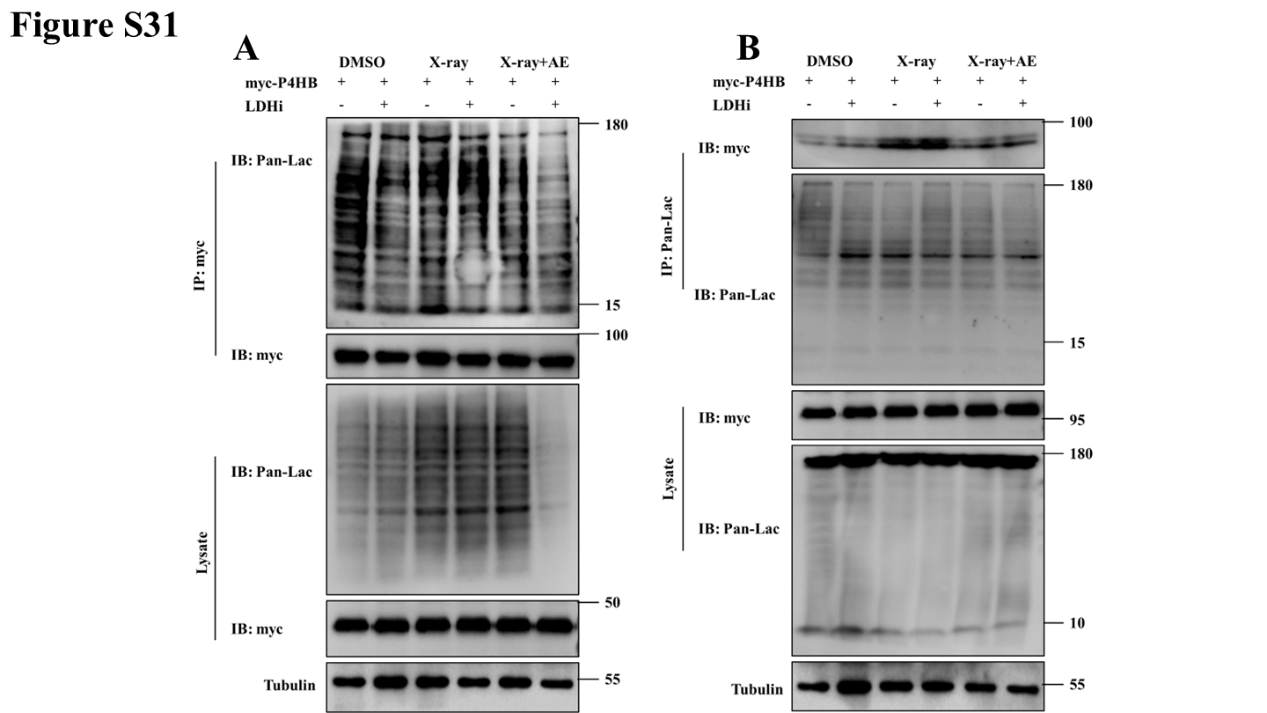


**Figure S31.** A, B) Co-immunoprecipitation assay of interaction between P4HB and Pac-Lac in the context of aloe emodin and LDHi.


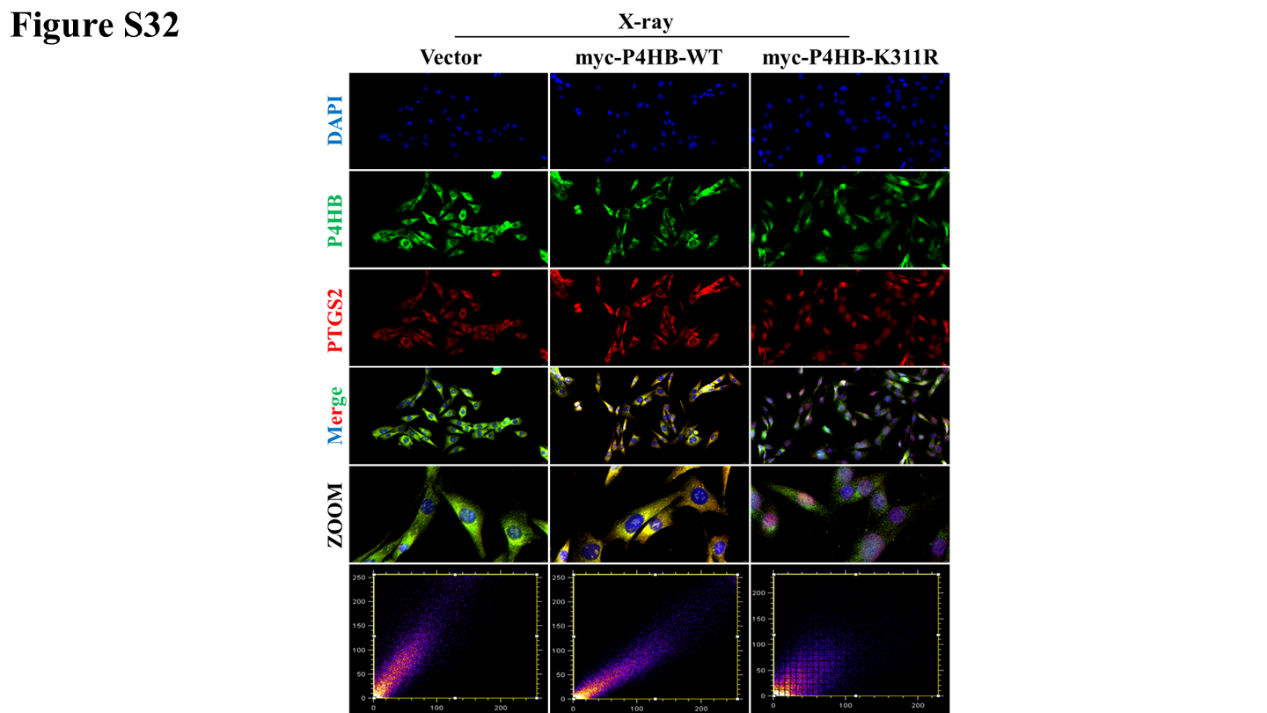


**Figure S32.** Co-localization of P4HB and PTGS2 after transfecting myc-P4HB-K311R. Scale bar, 5μm.


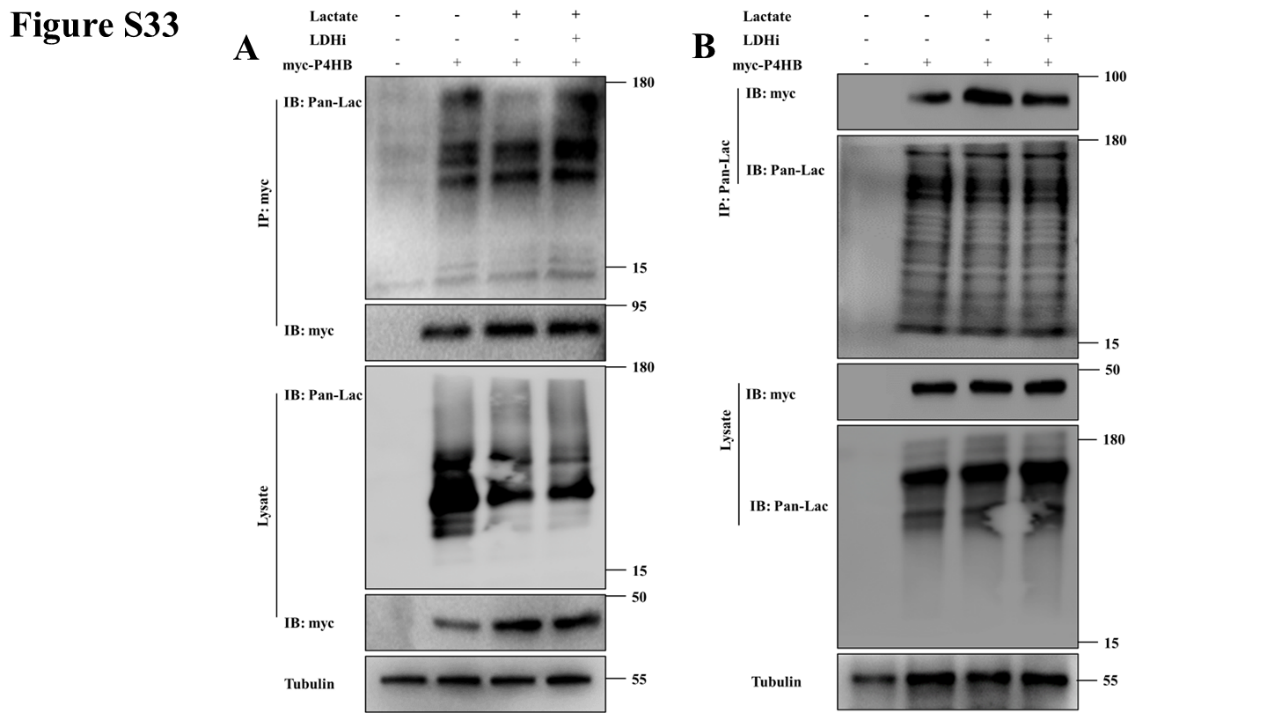


**Figure S33.** Promotion of interaction between Pan-Lac and P4HB by lactate incubation was inhibited by LDHi, using co-immunoprecipitation.


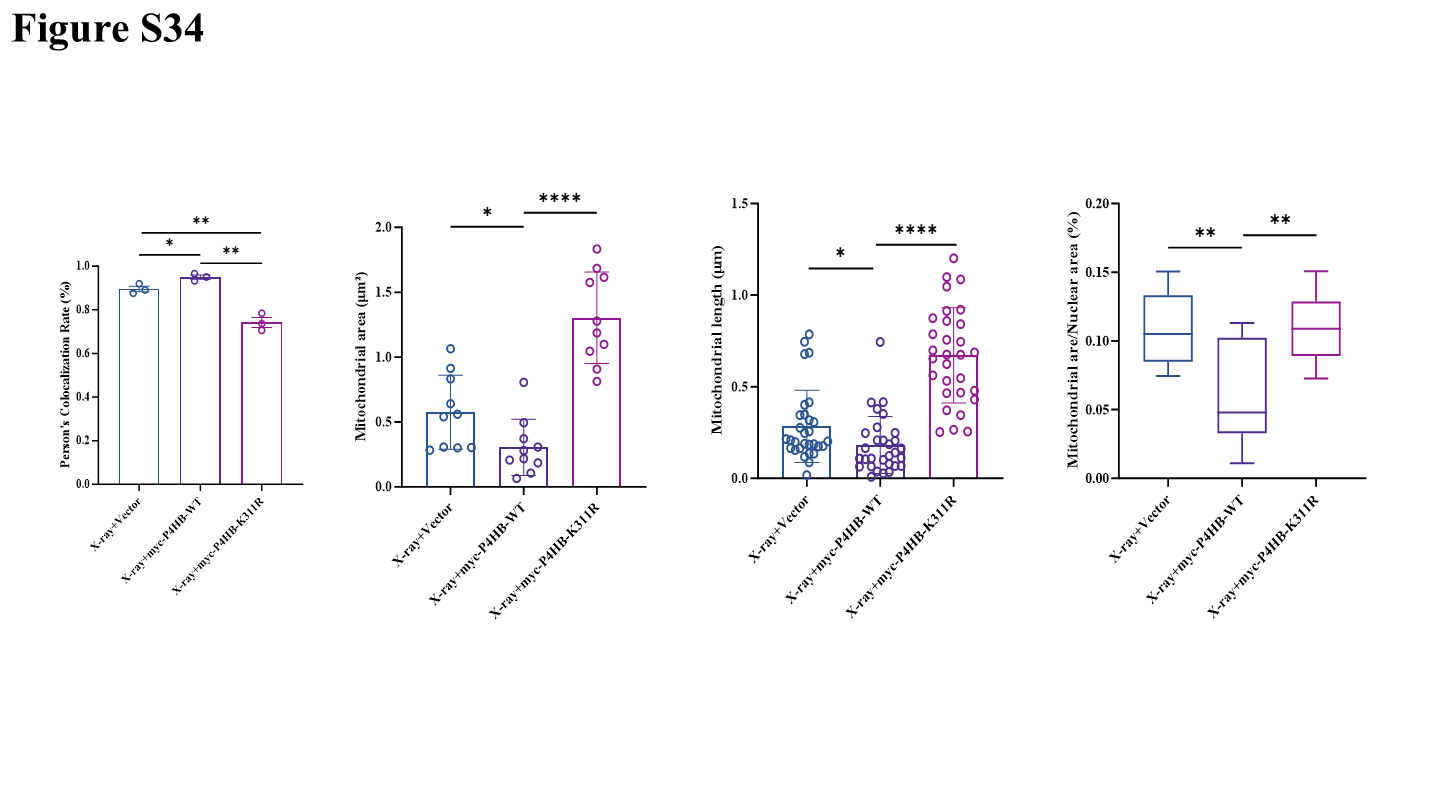


**Figure S34.** Quantification data for TEM analysis of H9C2 cells treated with K311R mutation plasmid. Quantification data are shown as mean ± SEM. All data were obtained from 3 independent experiments. *P* values were calculated by unpaired two-tail Student’s *t*-test or one-way ANOVA. **P* < 0.05; ***P* < 0.01; *****P* < 0.0001.


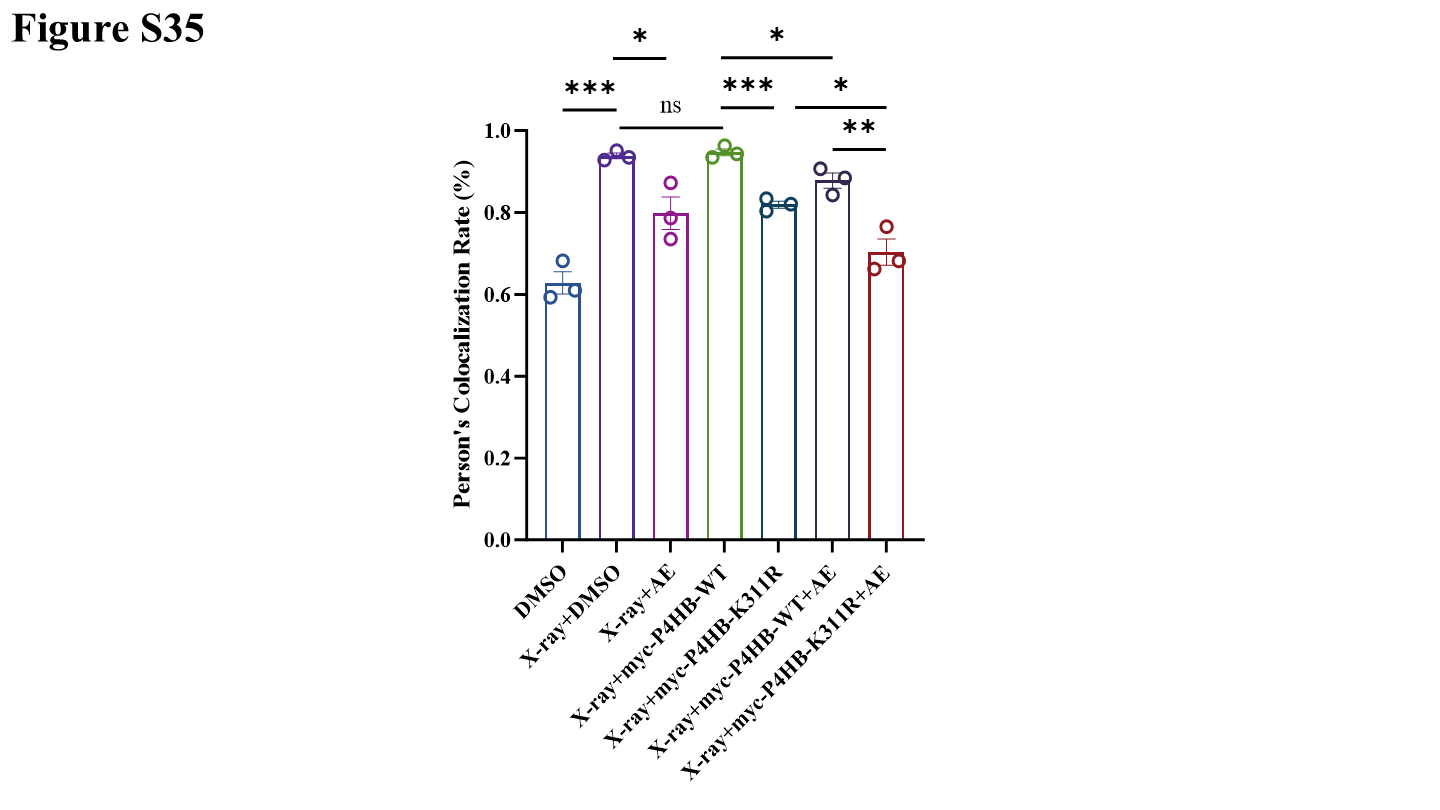


**Figure S35.** Quantification data for co-localization of TOMM20 and LAMP2A in the context of aloe emodin treatment with K311R. Quantification data are shown as mean ± SEM. All data were obtained from 3 independent experiments. *P* values were calculated by unpaired two-tail Student’s *t*-test or one-way ANOVA. **P* < 0.05; ***P* < 0.01; ****P* < 0.001; ns: not significant.


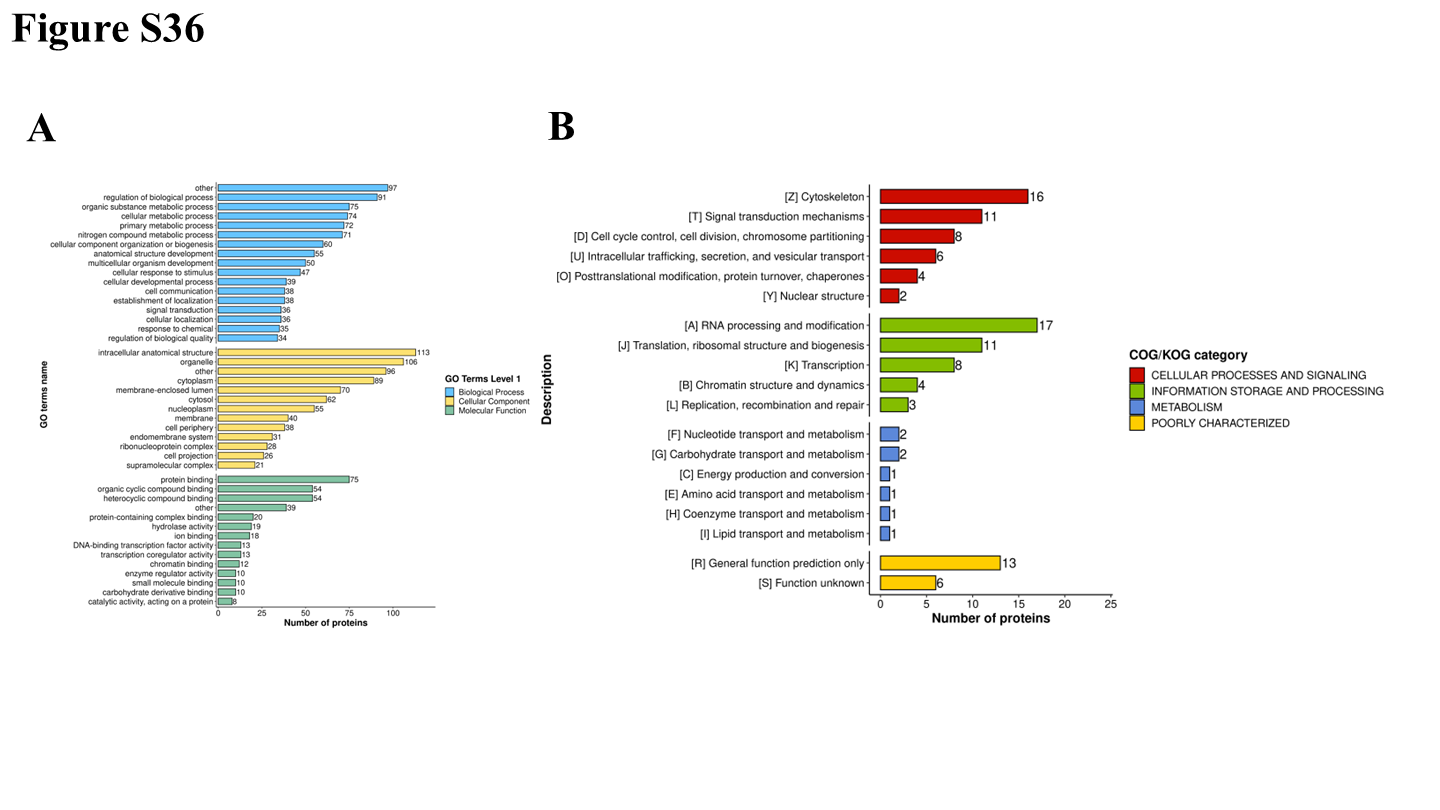


**Figure S36.** A, B) Enrichment analysis of selected proteins from lactylation omics in cellular metabolic processes and amino acid transport and metabolism by KEGG and GO.


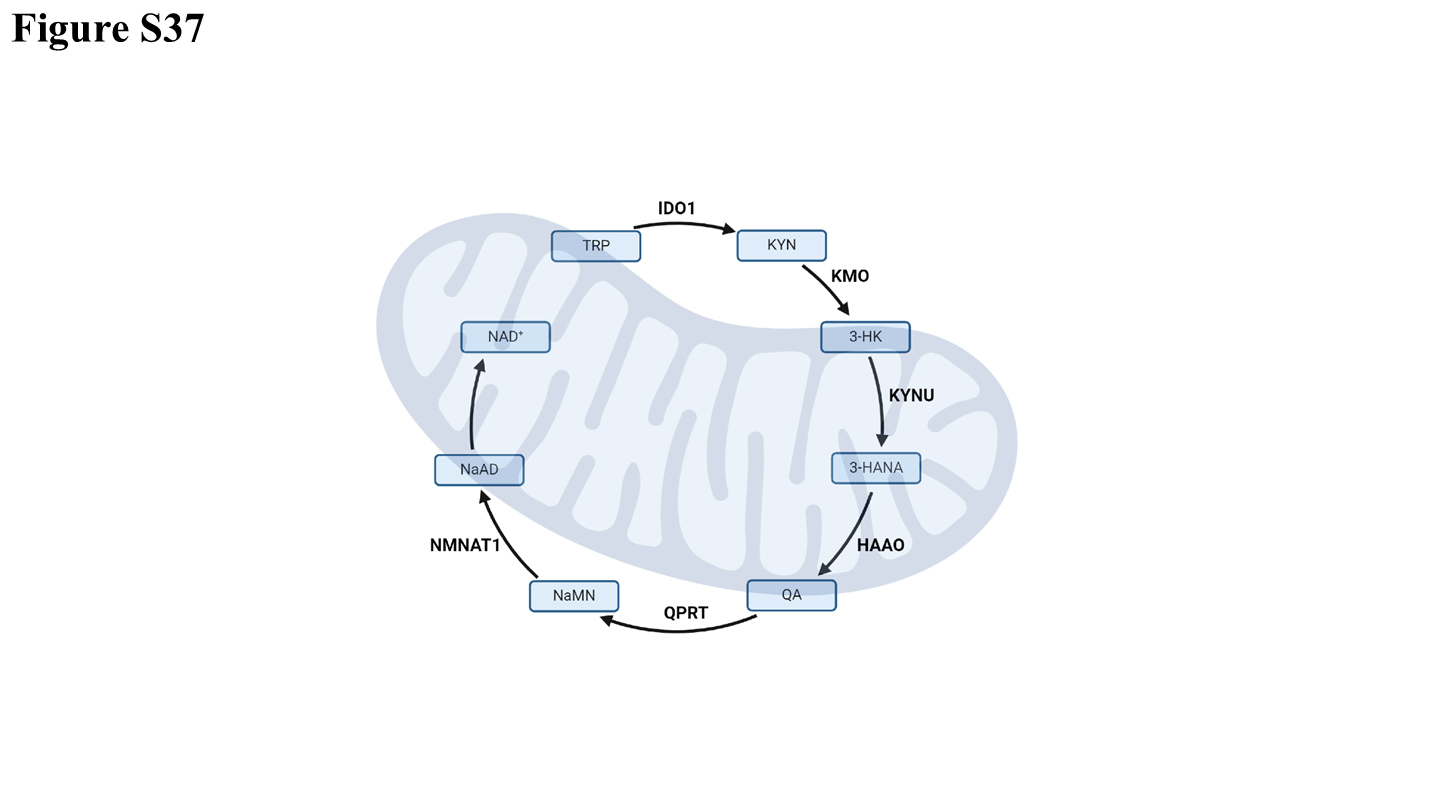


**Figure S37.** Flowchart of mitochondrial kynurenine metabolism. The key rate-limiting enzymes, including IDO1, KMO, KYNU, HAAO, QPRT and NMNAT1, were sequentially regulate metabolism of tryptophan, kynurenine, 3-hydroxy-kynurenine, 3-hydroxyanthranilic acid, quinolinic acid, nicotinic acid mononucleotide and nicotinamide adenine dinucleotide, respectively.


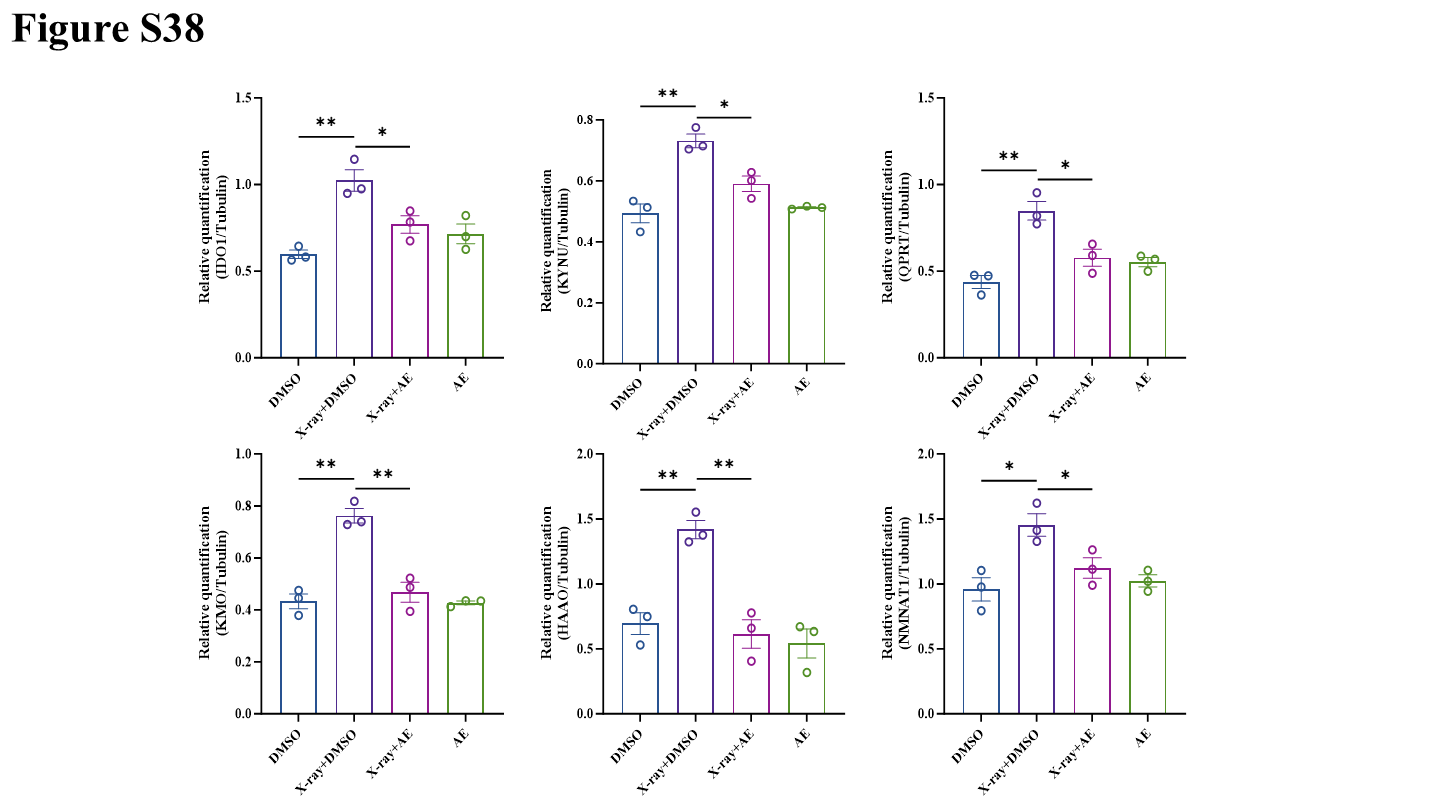


**Figure S38.** Quantification data for protein levels of IDO1, KMO, KYNU, HAAO, QPRT and NMNAT1 by emodin treatment. Quantification data are shown as mean ± SEM. All data were obtained from 3 independent experiments. *P* values were calculated by unpaired two-tail Student’s *t*-test or one-way ANOVA. **P* < 0.05; ***P* < 0.01.


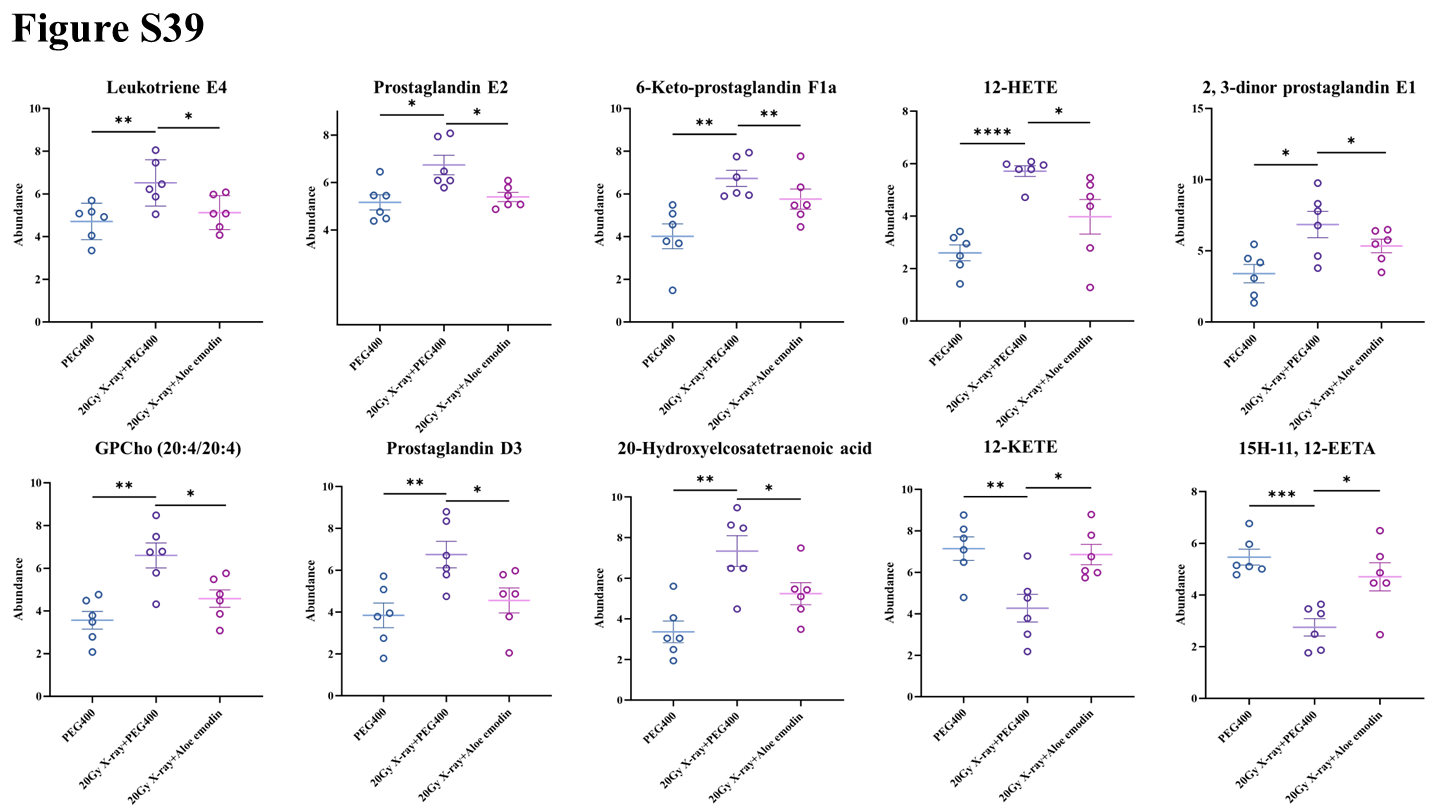


**Figure S39.** Metabonomics combined with ELISA assay for analysis of 10 metabolites from arachidonic acid metabolism in rat serum samples. Quantification data are shown as mean ± SEM. All data were obtained from 6 independent experiments. *P* values were calculated by unpaired two-tail Student’s *t*-test or one-way ANOVA. **P* < 0.05; ***P* < 0.01; ****P* < 0.001; *****P* < 0.0001.


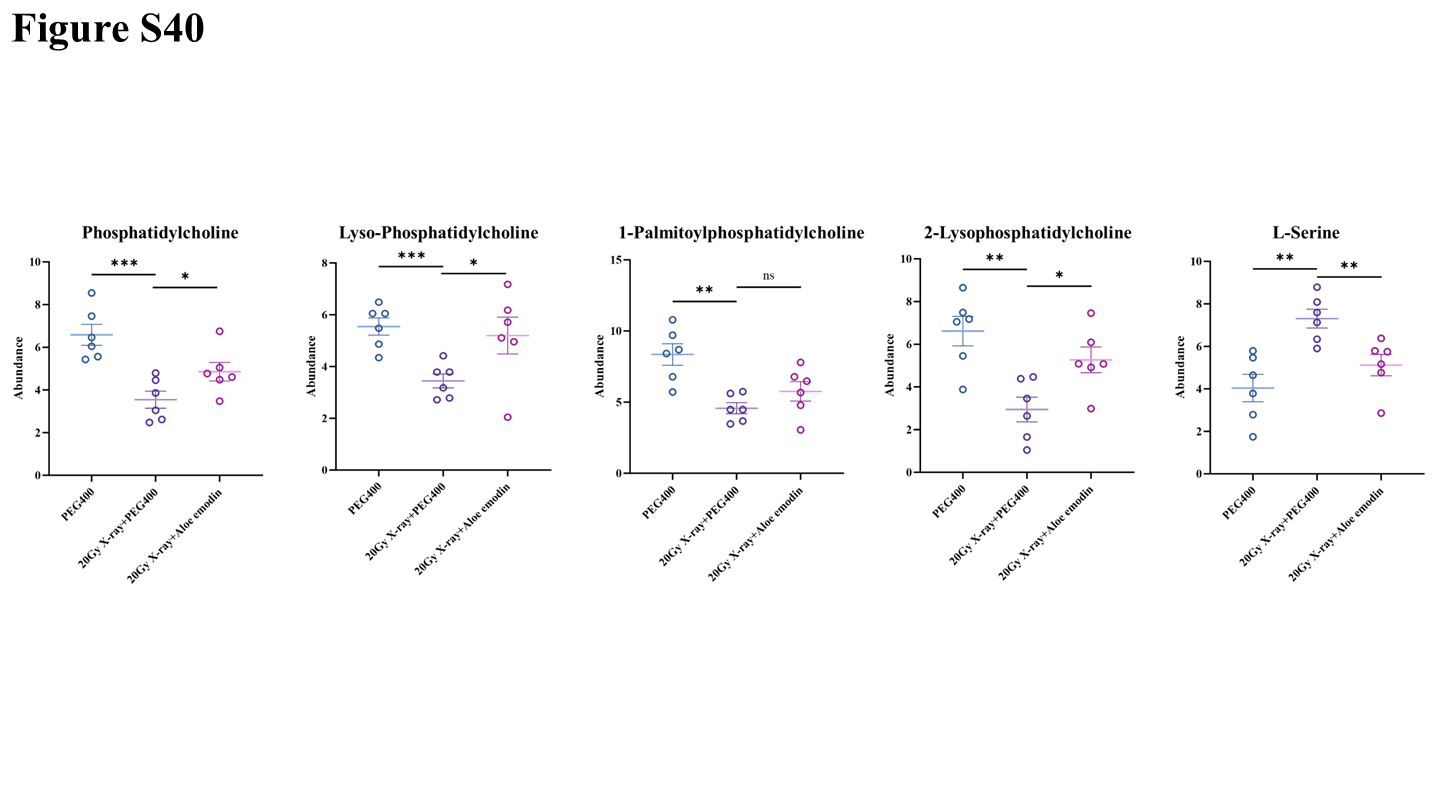


**Figure S40.** Metabonomics combined with ELISA assay analyzing 5 metabolites from glycerophospholipid metabolism in rat serum samples. Quantification data are shown as mean ± SEM. All data were obtained from 6 independent experiments. *P* values were calculated by unpaired two-tail Student’s *t*-test or one-way ANOVA. **P* < 0.05; ***P* < 0.01; ****P* < 0.001; ns: not significant.


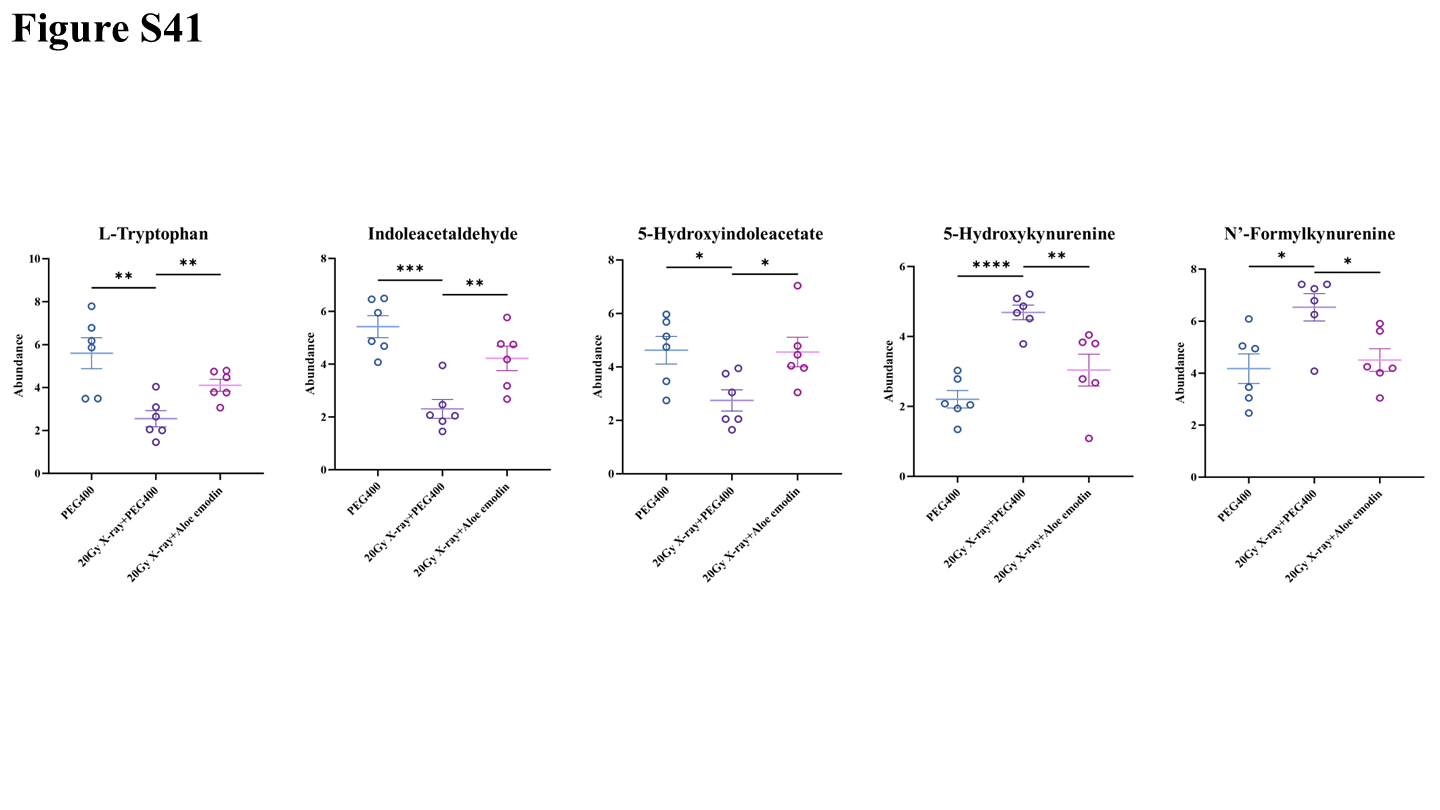


**Figure S41.** Metabonomics combined with ELISA assay analyzing 5 metabolites from tryptophan metabolism in rat serum samples. Quantification data are shown as mean ± SEM. All data were obtained from 6 independent experiments. *P* values were calculated by unpaired two-tail Student’s *t*-test or one-way ANOVA. **P* < 0.05; ***P* < 0.01; ****P* < 0.001; *****P* < 0.0001.


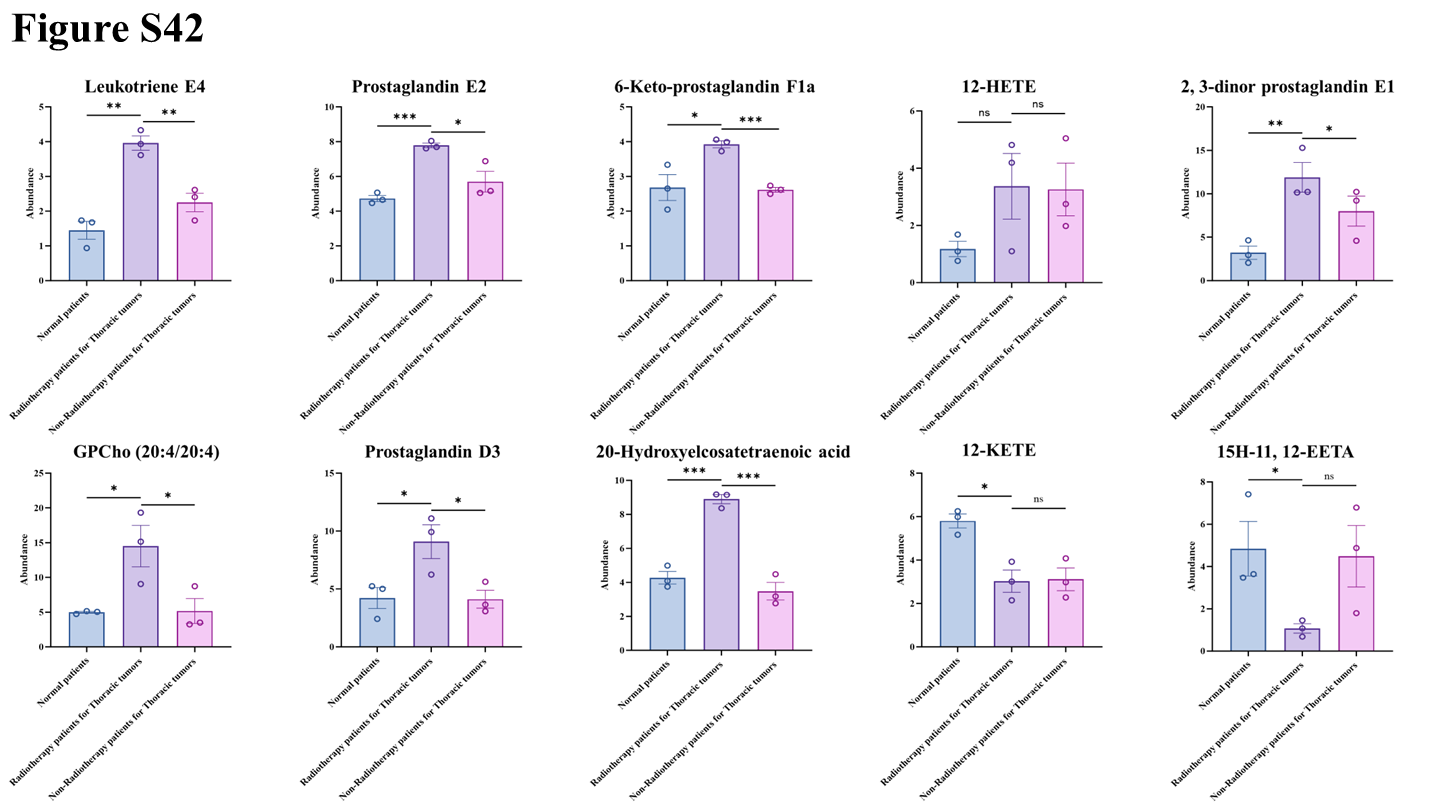


**Figure S42.** Metabonomics combined with ELISA assay analyzing 10 metabolites from arachidonic acid metabolism in human serum samples. Quantification data are shown as mean ± SEM. All data were obtained from 3 independent experiments. *P* values were calculated by unpaired two-tail Student’s *t*-test or one-way ANOVA. **P* < 0.05; ***P* < 0.01; ****P* < 0.001; ns: not significant.


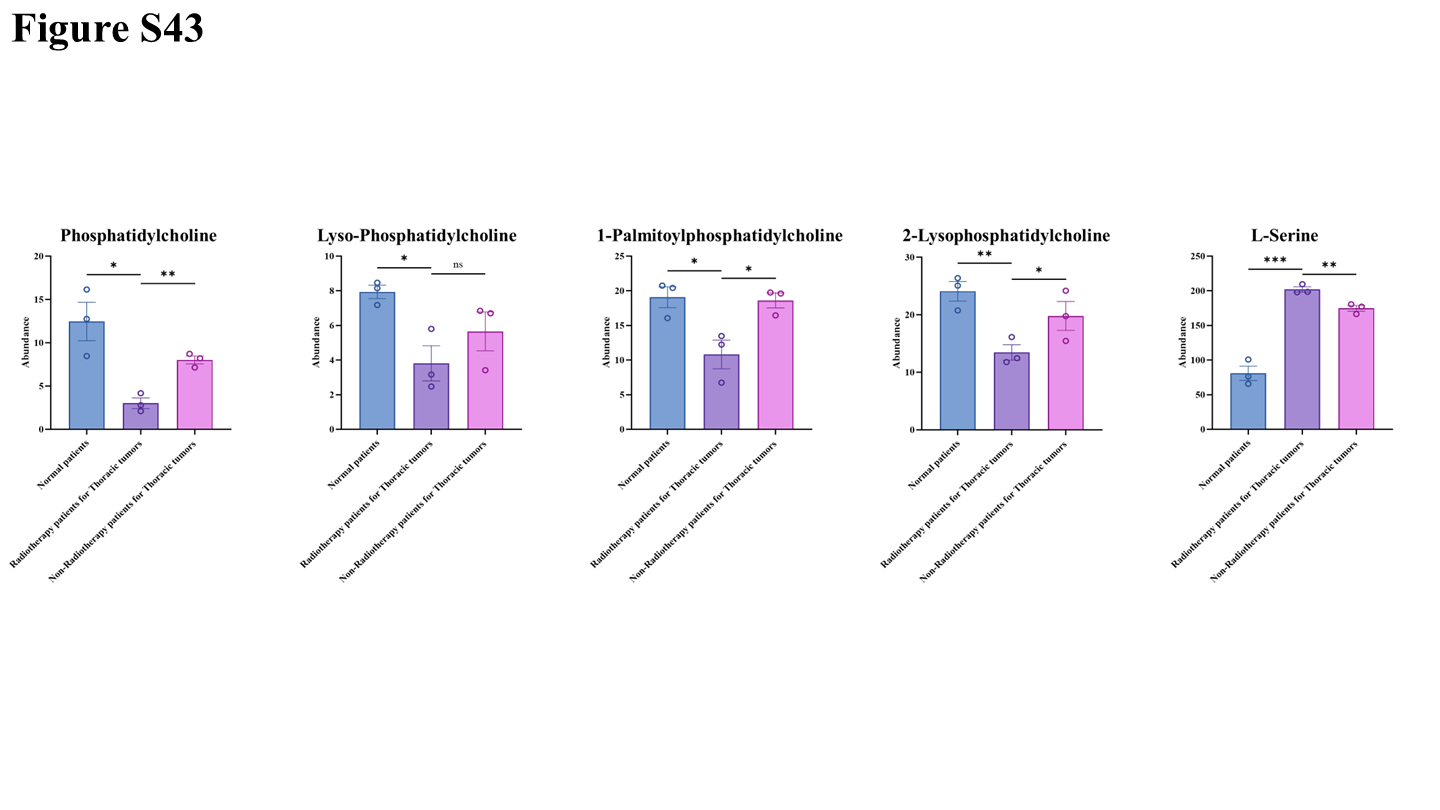


**Figure S43.** Metabonomics combined with ELISA assay analyzing 5 metabolites from glycerophospholipid metabolism in human serum samples. Quantification data are shown as mean ± SEM. All data were obtained from 3 independent experiments. *P* values were calculated by unpaired two-tail Student’s *t*-test or one-way ANOVA. **P* < 0.05; ***P* < 0.01; ****P* < 0.001; ns: not significant.


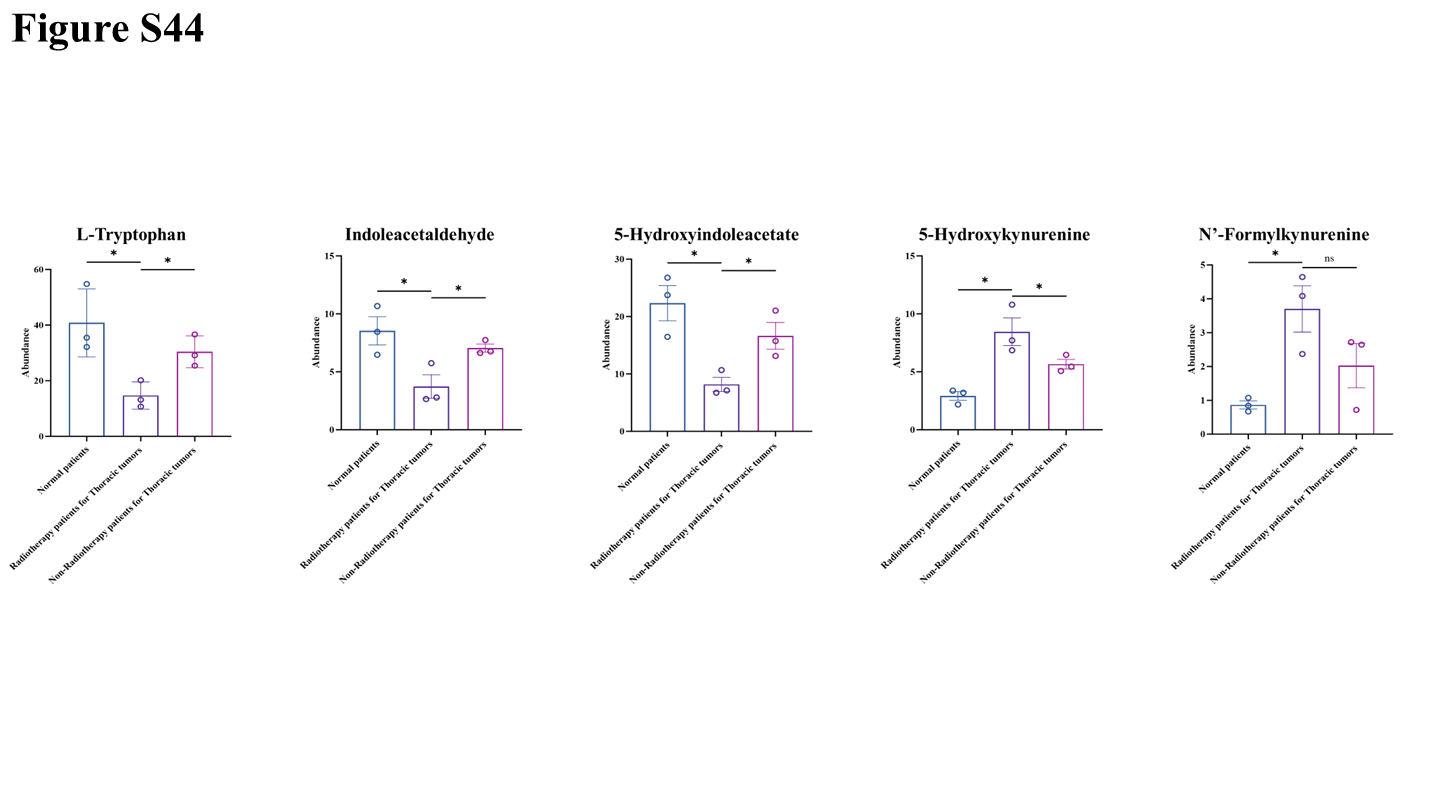


**Figure S44.** Metabonomics combined with ELISA analyzing 5 metabolites from tryptophan metabolism in human serum samples. Quantification data are shown as mean ± SEM. All data were obtained from 3 independent experiments. *P* values were calculated by unpaired two-tail Student’s *t*-test or one-way ANOVA. **P* < 0.05; ns: not significant.

S
